# Flaws in foldamers: conformational uniformity and signal decay in achiral helical peptide oligomers†
†Electronic supplementary information (ESI) available: Synthesis and characterisation of all new compounds. See DOI: 10.1039/c4sc03944k



**DOI:** 10.1039/c4sc03944k

**Published:** 2015-01-21

**Authors:** Bryden A. F. Le Bailly, Liam Byrne, Vincent Diemer, Mohammadali Foroozandeh, Gareth A. Morris, Jonathan Clayden

**Affiliations:** a School of Chemistry , University of Manchester , Oxford Road , Manchester M13 9PL , UK . Email: clayden@man.ac.uk

## Abstract

Although foldamers, by definition, are extended molecular structures with a well-defined conformation, minor conformers must be populated at least to some extent in solution. We present a quantitative analysis of these minor conformers for a series of helical oligomers built from achiral but helicogenic α-amino acids. By measuring the chain length dependence or chain position dependence of NMR or CD quantities that measure screw-sense preference in a helical oligomer, we quantify values for the decay constant of a conformational signal as it passes through the molecular structure. This conformational signal is a perturbation of the racemic mixture of *M* and *P* helices that such oligomers typically adopt by the inclusion of an N or C terminal chiral inducer. We show that decay constants may be very low (<1% signal loss per residue) in non-polar solvents, and we evaluate the increase in decay constant that results in polar solvents, at higher temperatures, and with more conformationally flexible residues such as Gly. Decay constants are independent of whether the signal originates from the N or the C terminus. By interpreting the decay constant in terms of the probability with which conformations containing a screw-sense reversal are populated, we quantify the populations of these alternative minor conformers within the overall ensemble of secondary structures adopted by the foldamer. We deduce helical persistence lengths for Aib polymers that allow us to show that in a non-polar solvent a peptide helix, even in the absence of chiral residues, may continue with the same screw sense for approximately 200 residues.

## Abstract 2

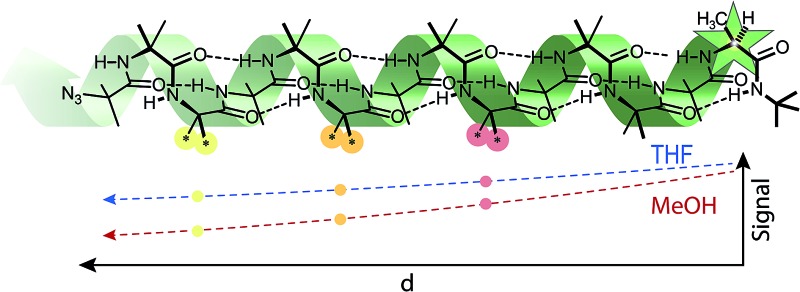
The conformational influence of a single stereogenic centre in an otherwise achiral oligomer behaves as a signal that decays with distance.

## Introduction

A foldamer is ‘a polymer [or oligomer] with a strong tendency to adopt a specific compact conformation’,^1^ and foldamers^2–6^ can typically be viewed as synthetic analogues of biopolymers – peptides, proteins and nucleic acids.^7^ The three-dimensional structure of such biopolymers is in many cases characterised by the existence of one particularly favourable conformation, with this gross conformational uniformity being intimately linked with function. Examples include the helical conformation of DNA under normal conditions^8^ and structural proteins such as collagen.^9^ However, many other biopolymers exhibit more complex conformational behaviour in which alternative conformers may be populated. Classic examples^10^ include allosterically switchable molecules such as haemoglobin^11^ or phosphorylase enzymes,^12^ and conformationally switchable proteins involved in signal transduction.^13,14^ Yet other biopolymers appear to be almost fully unstructured in a three-dimensional sense, with many energetically similar alternative conformational states being populated.^15^ Synthetic analogues of these less conformationally uniform biomolecules should form their own group within the general class of foldamers, comprising structures to which more than one well defined ‘specific compact conformation’ is available. Indeed, entropic considerations make it reasonable to expect that even the most conformationally well defined foldamers must spend at least some of their time in minor, less favourable conformations. Studies of the relative tendency towards helicity of alternative monomer units have been made in some foldamer classes by exploring their ability to communicate a screw-sense preference between two helical domains.^16,17^ More detailed exploration by classic spectroscopic techniques of the multiple conformers populated by some peptidomimetic structures poses well-established difficulties.^18–20^ For this reason, the intrusion of alternative conformers into foldamer structures of uniform primary sequence has rarely been quantified, explored, or exploited.^21^ Nonetheless, such studies are essential if the broader field of foldamer chemistry is to deliver biomimetic function as well as biomimetic structure.

**Fig. 1:**
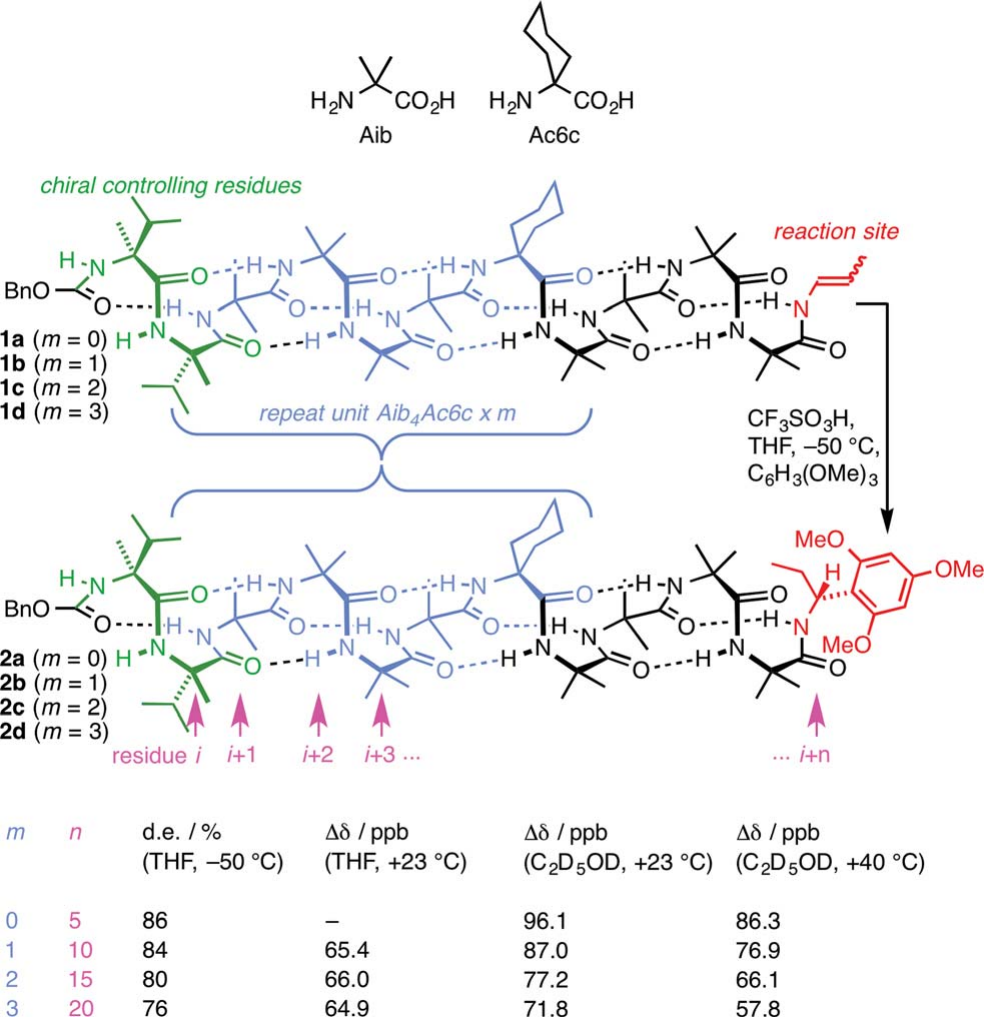
Remotely induced diastereoselective formation of diastereoisomeric foldamers **2a–d** and the solvent-dependent chemical shift separation between their CH signals.

Oligomers of achiral quaternary amino acids, of which 2-aminoisobutyric acid (Aib) is the archetype, but which also include 1-aminocyclohexanecarboxylic acid (Ac6c, Fig. 1) along with 1-aminocyclopentanecarboxylic acid (Ac5c), diethylglycine (Deg) *etc.* ^22,23^ form a class of foldamers that adopt specific compact conformations that take the form of hydrogen-bonded 3_10_ helices.^22,24–28^ These helices are necessarily equal mixtures of left- and right-handed screw-sense conformers because (unlike natural peptides or nucleic acids) the monomers themselves are achiral.^29^ Nonetheless an equal population of the *M* and *P* conformers may be detectably perturbed by incorporation of one or more terminal chiral residues^30–39^ or interaction with chiral ligands.^40–44^ A helical chain of achiral monomers may be switched between a left- and a right-handed screw-sense preference either by configurational inversion of a single chiral residue at one terminus,^45^ or by reversible covalent^40^ or non-covalent^46,47^ binding of a chiral ligand to a terminal binding site. Such foldamers exhibit a form of switchability analogous to membrane-bound receptors, in which a conformational response to the binding of a ligand mediates signal transduction through the thickness of the cell membrane to mediate a remote biochemical outcome.^13^

We have exploited this ability of helical foldamers to communicate information by propagation of a conformational preference in some reactions exhibiting remote stereochemical control.^48^ Two chiral l-α-methylvaline (α-MeVal) residues at the terminus of a peptide-like oligomer of achiral quaternary amino acids are sufficient to induce essentially complete control over screw-sense preference which persists far enough to allow reactions to take place with 1,61 asymmetric induction and 88 : 12 diastereoselectivity. Similar reactions over shorter distances (1,46, 1,31 and 1,16 asymmetric induction) show successively increased levels of stereoselectivity. This chain-length dependence^49^ indicates that the ability of the helical foldamer to communicate stereochemical information is high, but not perfect (Fig. 1).

We may consider a conformational perturbation such as the screw-sense preference induced by the chiral N-terminal l-α-MeVal residues of **1** as a signal^50–52^ that propagates through the polymeric molecule without erosion only if that molecule truly adopts one single ‘specific compact conformation’.^1^ Any random intrusion of alternative conformations must attenuate the signal, allowing the local conformational preference within the helix to decay towards an equal population of left- and right-handed helices the further the polymer is extended away from the chiral terminus.

It is the occurrence of these alternative minor foldamer conformations in the solution state that we explore in this paper. We use NMR and other spectroscopic methods to quantify the attenuation of a conformational preference with increasing distance from a source of screw-sense induction located at either terminus of a series of Aib-containing foldamers. We show that a conformational signal is in many cases remarkably persistent, but its decrease in intensity on moving in either direction through the polymer may be characterized as a solvent-dependent exponential decay. We interpret the spatial decay constant of the signal in terms of the stochastic intrusion of screw-sense reversals into the three-dimensional structure of the oligomers, and use the results to quantify the population of minor contributors to the overall ensemble of conformations that describe the dynamic three-dimensional structure of the foldamer.

## Results

### Solvent-dependent signal decay moving away from the N terminus

**Fig. 2:**
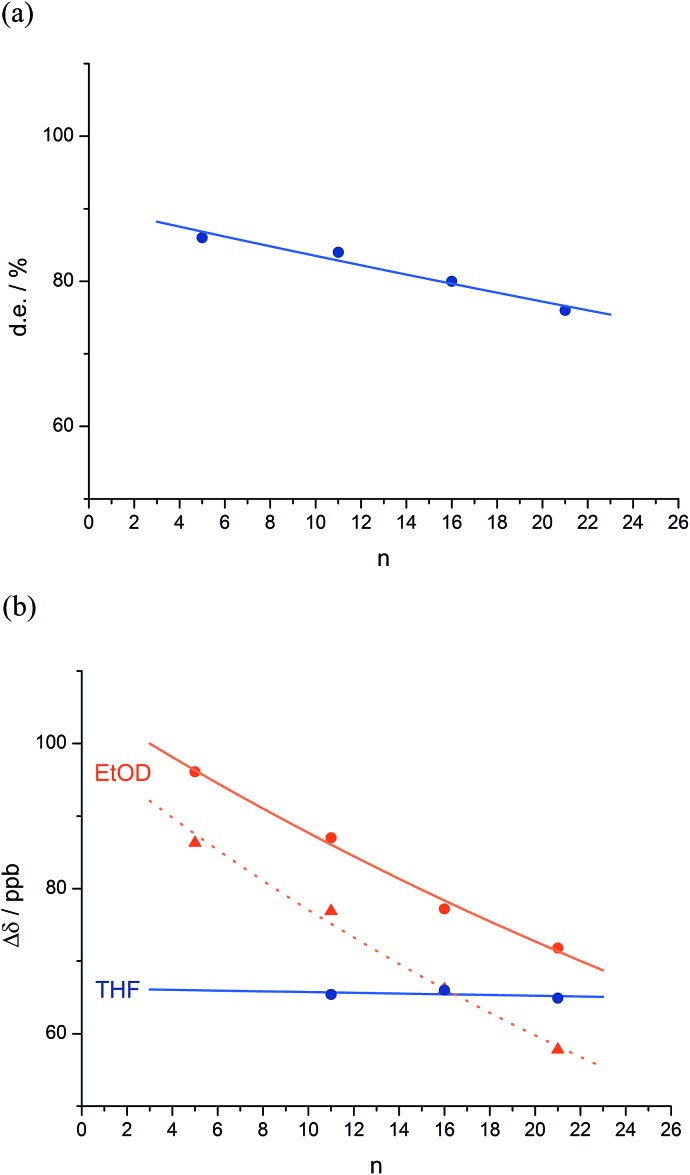
Effect of chain length *n* on characteristic features of the products **2a–d**. (a) Exponential decay of diastereoselectivity (diastereoisomeric excess, de) with chain length (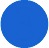 reactions in THF at –50 °C; 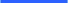 *f* = 0.008); (b) exponential decay of chemical shift separation Δ*δ* between CHN signals of diastereoisomeric pairs (*S*,*S*,*S*) and (*S*,*S*,*R*)-**2** (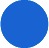 in d_8_-THF, 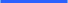 *f* = 0.001; 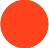 in C_2_D_5_OD at +23 °C, 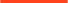 *f* = 0.019; 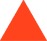 in C_2_D_5_OD at +40 °C, 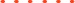 *f* = 0.025). The typical uncertainty in *f* is of the order of ±0.002.

The reactions shown in Fig. 1 (ref. 48) exhibit remote stereochemical control as a result of quantitative induction by the N-terminal chiral dimer (shown in green) of a screw-sense preference in the achiral helical chain (shown in blue and black). As a result the reaction site (shown in red) finds itself in a non-racemic environment, and reacts diastereoselectively by face-selective attack on the arene on an acyliminium ion generated *in situ*.^53^ The diastereoselectivity (measured as diastereoisomeric excess, de) of the reaction is in each case the product of the induced helical excess^54–56^ at the N-terminal dimer (he_*i*_), the fidelity with which this helical excess is communicated to the C terminus, and the diastereofacial selectivity (ds) with which a pure *M* or *P* helix would induce attack on the iminium ion intermediate in the acid-catalysed reaction. As the first and last of these factors are constant in all four reactions, the decay in diastereoselectivity in the reactions of the oligomers **1a–d** in Fig. 1, from 93 : 7 dr (86% de) for a 1.16 relationship to 88 : 12 dr (76% de) for a 1.61 relationship, allows us to make an initial estimate of the effect of oligomer length on the ability of an Aib/Ac6c-containing helix to communicate a screw-sense preference. Fig. 2a shows diastereoselectivity of the reaction **1a–d** → **2a–d** plotted against the distance *n* in residues from the inducer of helical preference at residue *i* to the reaction site. On the assumption that Aib and Ac6c have equivalent conformational preferences,^57^ we fitted exponential decays to these curves, using the formula

1 de_*n*_ = he_*i*_ × (1 – *f*)^*n*^ × ds

where de_*n*_ is the diastereoisomeric excess induced in a reaction at residue *i* + *n*, he_*i*_ is the local helical excess induced by the N terminal l-α-MeVal dimer, *f* is the fraction by which he decreases on passing through each residue in the chain, and ds is the inherent diastereoselectivity of the reaction (*i.e.* the de that would result from a purely *M* or purely *P* helix).

We deduce that de decays by a factor of 0.8% per residue (*i.e. f* = 0.008) under the conditions of the reaction in THF at –50 °C.^58–60^ Given that when *n* = 0, de_*i*_ = he_*i*_ × ds, we can use de_0_ (extrapolated from Fig. 2a) = 90% and the reported value of he_0_ for (l-α-MeVal)_2_ of 99% (ref. 31) to deduce that the inherent diastereoselectivity of the reaction corresponds to a ds of *ca.* 95 : 5.

Constraints of temperature and solvent, which must be chosen to be compatible with the reaction in question, make diastereoselectivity an inconvenient observable quantity for the wider study helix of conformation. We therefore turned to the anisochronicity Δ*δ* separating the signals arising from the major and minor diastereoisomers of the products of this addition reaction, which also allowed us to measure the conformational preference of the oligomers in alternative solvents.

We previously described a simple spectroscopic method for quantifying the relative proportions of the two screw-sense conformers, the helical excess (he), by measuring the anisochronicity of a pair of signals arising from diastereotopic reporter groups embedded in the helical chain.^34^ In the fast exchange regime, the chemical shift separation of two such anisochronous peaks is proportional to the local he experienced by the NMR reporter group, such that

2 he = Δ*δ*_fast_/Δ*δ*_slow_

where Δ*δ*_fast_ and Δ*δ*_slow_ represent the measured anisochronicity of the peaks in the fast and the slow exchange regime. We have applied the method to diastereotopic ^13^C (ref. 34), ^1^H (ref. 31 and 33) and ^19^F (ref. 61) signals: in each case, room temperature NMR spectra lie in the fast exchange regime for typical chemical shift separations.^29,62–64^

The same arguments must hold for pairs of signals arising from corresponding groups in diastereoisomeric structures. The detailed factors governing their anisochronicity will be more complicated to analyse, but these detailed conformational considerations are irrelevant to the extraction of an exponential decay factor. A decrease in the fast exchange regime anisochronicity of peaks arising from two diastereoisomers on increasing the length of the achiral segment separating the stereogenic centres that give rise to their diastereoisomeric relationship may be interpreted simply as a measure of that segment's decreasing ability to communicate stereochemical information between the relevant stereogenic centres.

Fig. 1 shows how the separation Δ*δ* (= Δ*δ*_fast_ of eqn (2)) between the CHN signals of the diastereoisomers (*S*,*S*,*S*) and (*S*,*S*,*R*)-**2a–d** decreases with increasing chain length at 23 °C in deuterated THF and at +23 °C or 40 °C in a more polar solvent, deuterated ethanol, and Fig. 2b plots anisochronicity against *n* (the length of the chain lying between the centres) for these solvents. Poor solubility prevented us studying these oligomers in chloroform. We fitted the points to a modification of the formula used earlier

3 Δ*δ*_*n*_ = Δ*δ*_*i*_ × (1 – *f*)^*n*^

where Δ*δ*_*n*_ is the anisochronicity of the CHN signals when they are located at residue *i* + *n* and Δ*δ*_*i*_ simply a limiting value to which we assign no chemical significance. Decay constants *f* (the per-residue decrease in Δ*δ*) can be extracted of 0.1% for THF at 23 °C, 1.9% for ethanol at 23 °C, and 2.5% for ethanol at 40 °C. The near-zero value of the decay constant in THF confirms the observation from Fig. 2b that the screw-sense preference is highly persistent in this solvent. A more polar solvent and a higher temperature lead to a greater rate of decay, presumably by facilitating the breaking of intramolecular hydrogen bonds and by increasing the population of alternative minor conformers.

**Fig. 3:**
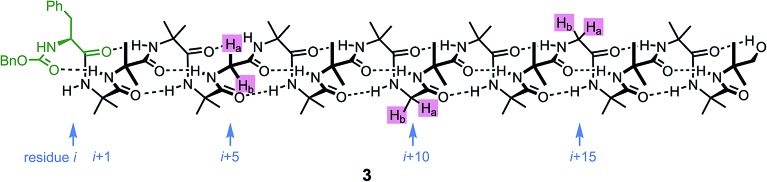
Three Gly residues as NMR reporters of helical excess embedded in an Aib-rich oligomer.

**Fig. 4:**
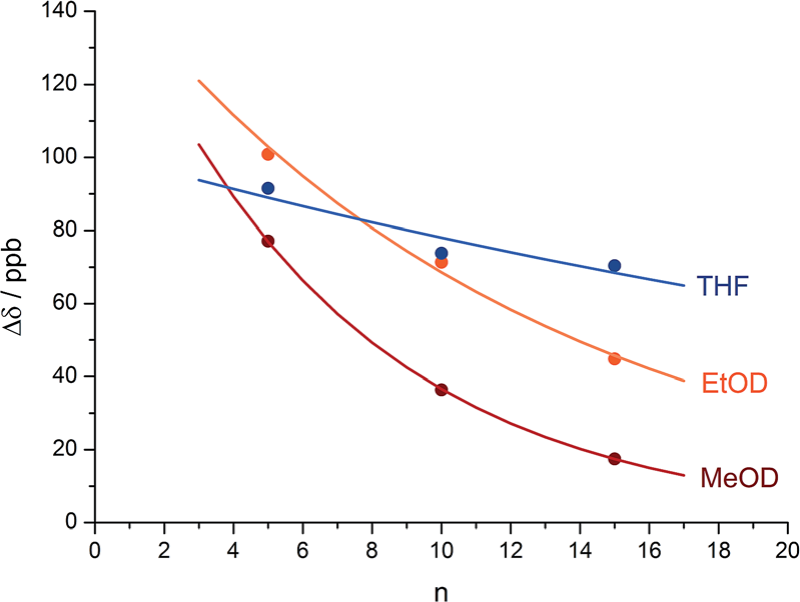
Anisochronicity Δ*δ* in the three Gly residues of **3** in THF, C_2_D_5_OD (23 °C and 40 °C) and CD_3_OD. 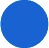 THF at 23 °C, 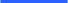 *f* = 0.026; 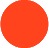 ethanol at 23 °C, 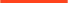 *f* = 0.078; 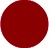 methanol at 23 °C, 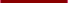 *f* = 0.14.

An alternative, simpler measure of signal decay may be obtained by inserting several NMR reporters, each containing a pair of diastereotopic groups giving rise to potentially anisochronous signals, at successive positions along a helical oligomer. Provided that the reporter groups are sufficiently far from the ends of the chain for their anisochronicity to be dictated solely by their local helical environment, the decrease in their anisochronicity along the chain gives a measure of signal decay. The challenge in such an approach is identifying which of several identical reporters is which. This is less difficult if the rate at which anisochronicity decreases is relatively high, because the signals from successive reporters are not superimposed upon one another, and a simplifying assumption can be made that anisochronicity can only decrease along the chain. We applied this principle to oligomer **3** (Fig. 3)^65^ which contains three glycine residues. These serve the dual purpose of firstly introducing greater flexibility than Aib, and therefore a steeper rate of decay of screw-sense preference, and secondly of providing pairs of diastereotopic protons as spectroscopic reporters of screw-sense preference. In ethanol and methanol, the three glycine ABX systems were resolved by Lorentz–Gauss transformation, while in CDCl_3_ and in CD_2_Cl_2_ poor dispersion prevented reliable assignment. In THF, however, the A and B coupled signals of the three ABX spin systems were identified using an absorption-mode 2D J-resolved experiment based on the PSYCHE method.^66,67^ Fig. 4 plots the fast exchange anisochronicity Δ*δ* between the A and B components of the AB systems^68^ against the position *n* of the relevant Gly residue relative to the N terminal Cbz-l-Phe at position *i*, and in each case a value for *f* was obtained using eqn (3).

This time, the additional conformational flexibility complicates the analysis of the decay constants. Gly introduces much more flexibility than Aib into a peptide chain,^17^ so we expect the values of *f* for Aib (*f*_Aib_) and for Gly (*f*_Gly_) to differ significantly. There are four Aib residues for every Gly in the chain, so the value *f* obtained from fitting eqn (3) to Fig. 4 is therefore:

4 *f* = 0.8*f*_Aib_ + 0.2*f*_Gly_

The values of *f* obtained exhibit a solvent dependence consistent with that noted for **2**, with the more polar methanol allowing greater conformational flexibility than the less polar THF, and ethanol lying somewhere between. Using our previous estimates of values for *f*_Aib_ at +23 °C in THF, in ethanol (Fig. 2) and in methanol,^31,34^ we can further deduce values of *f*_Gly_ from eqn (4) indicating that glycine residues introduce a fall in Δ*δ* at +23 °C of 13% in THF, 31% in C_2_D_5_OD or 49% in CD_3_OD, confirming that the Gly residues introduce significantly more conformational flexibility than the Aib residues.

### Solvent-dependent signal decay moving away from the C terminus

Identification of individual residues in an all-Aib oligomer is more challenging, because of the poor dispersion of the Aib methyl groups, which all lie within a <0.35 ppm range in the ^1^H NMR spectrum. Dispersion in the ^13^C NMR spectrum is better, and individual residues may be labelled by means of ^13^C-enriched methyl groups.^34^ However, difficulties of signal assignment remain, firstly because signals are likely to overlap when different parts of the oligomer exhibit a similar helical excess (in other words, when decay constants are low), and secondly because diastereotopic pairs of signals are harder to identify because they are not related to each other by coupling as they are in the ^1^H NMR spectra of oligomers containing germinal CH_2_ groups. We have previously shown, however, that successive members of a series of ^13^C labels may themselves each be labelled by changing their ^13^C abundance through successive dilutions with ^12^C, allowing paired diastereotopic labels to be identified by relative integrals.^34^

**Fig. 5:**
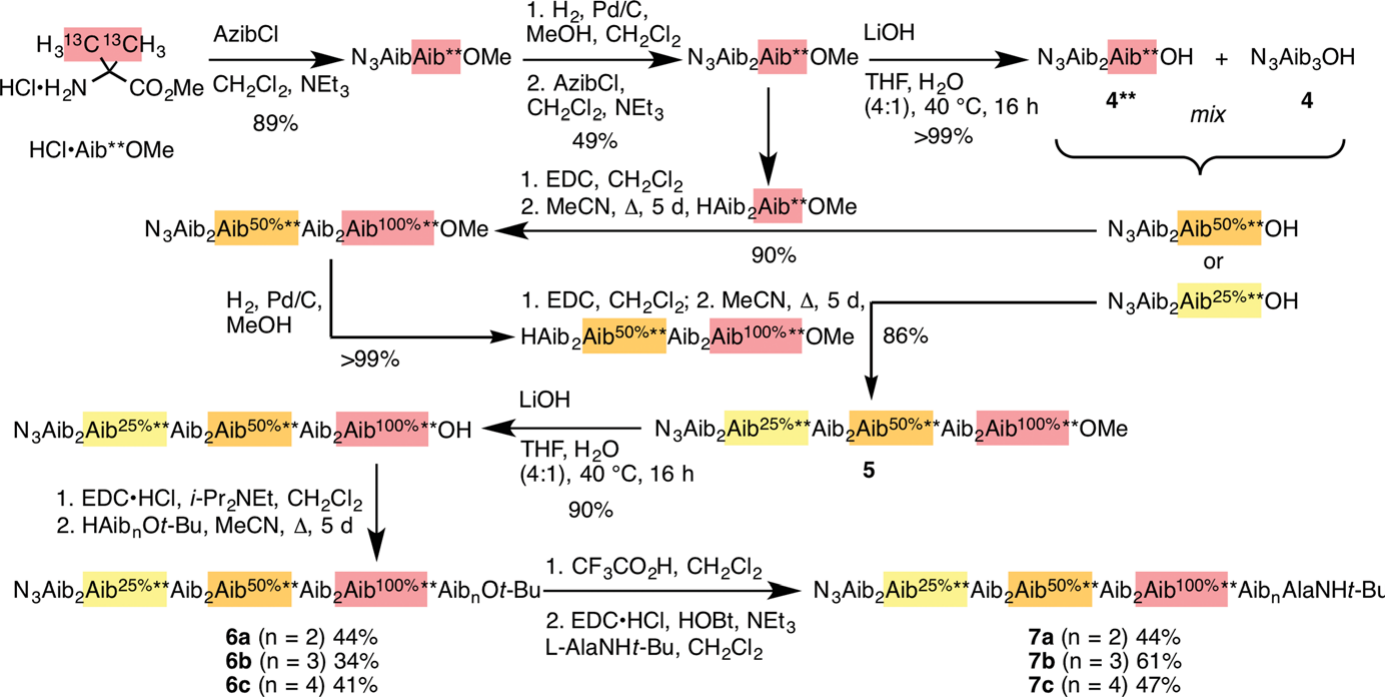
Synthesis of oligomers **7a–c** with residues identified by abundance-labelled ^13^C labels.

We used this approach to explore the communication of conformational information through an Aib oligomer from the C terminus to the N terminus. The multiply labelled compounds **7** were synthesized using the strategy illustrated in Fig. 5. Labelled and unlabelled Aib trimers 4** and **4** were built by standard methods from a starting monomer that was either 100% doubly (4**) or 1.1% singly (**4**, *i.e.* natural abundance) ^13^C labelled in its Aib methyl groups. The two samples of trimer 4** and **4** were then mixed in ratios of 50 : 50 or 25 : 75 and these two samples sequentially ligated to the methyl ester of 4** to make a nonamer **5**, three of whose residues are labelled with different abundances of ^13^C, as shown in Fig. 5. **5** was extended by ligation with an unlabelled Aib dimer, trimer or tetramer to make the three oligomers **6a–c**, which were each coupled at the C terminus to the powerful chiral controlling residue l-AlaNH*t*-Bu^32^ to provide 12-mer **7a**, 13-mer **7b** and 14-mer **7c**.

**Fig. 6:**
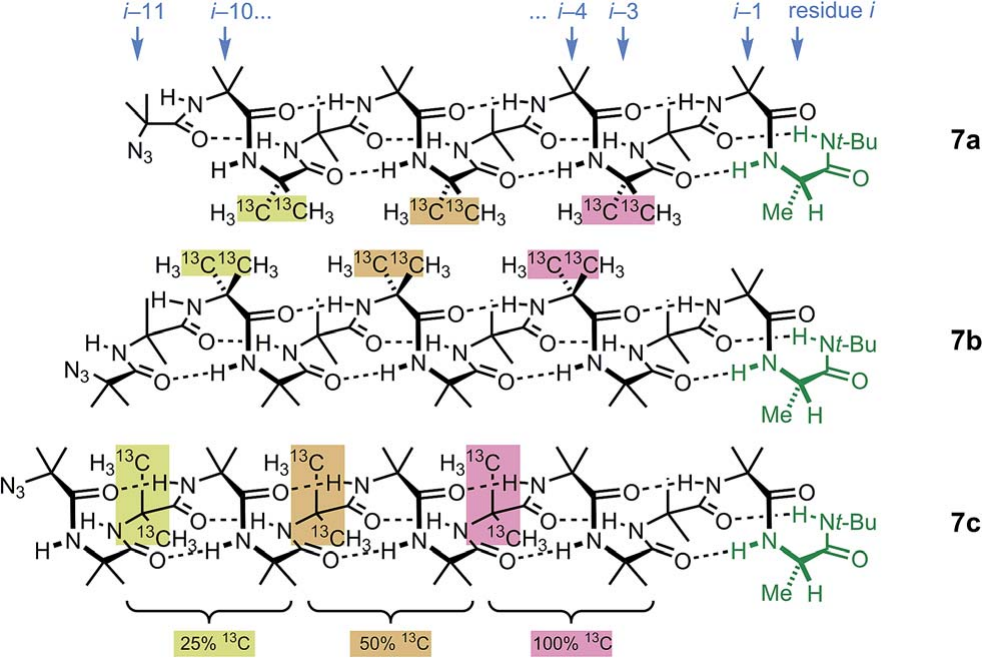
Multi-labelled oligomers **7a–c** allow measurement of screw sense at each of the nine positions in the oligomer from *i* – 3 to *i* – 11.

**Fig. 7:**
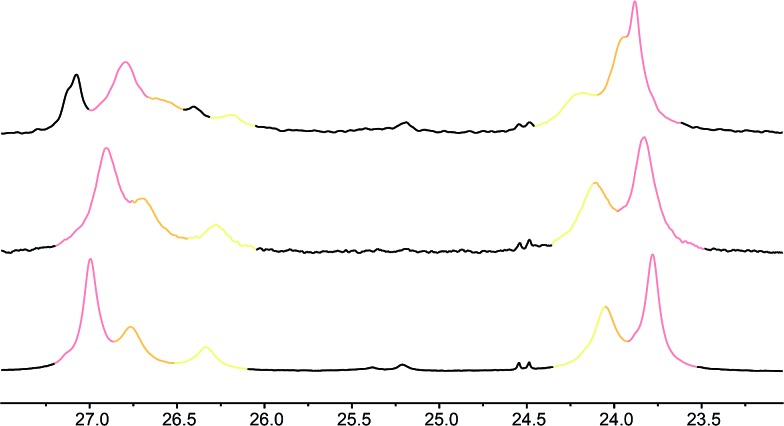
Illustrative ^13^C NMR spectra of **7a** (top), **7b** (middle) and **7c** (bottom) in 9 : 1 methanol : chloroform, with peaks arising from the 100%, 50% and 25% labels indicated by colours used in Fig. 6.

These three ‘frame-shifted’ oligomers between them contain a labelled probe at every one of the nine positions in the chain from *i* – 3 to *i* – 11, starting from the C terminal l-AlaNH*t*-Bu chiral controller at position *i* (Fig. 6). Only **7a** was soluble in pure methanol, but all were soluble in a 9 : 1 mixture of methanol and chloroform. The relevant portions of the three ^13^C spectra are shown in Fig. 7. The abundance-labelled labels are evident in the successively smaller anisochronicities of pairs of peaks with successively smaller integrals (100 : 50 : 25). Similar spectra in THF and in CDCl_3_ showed significant peak overlap due to the much lower decay of anisochronicity in these solvents, but individual peaks were still evident in THF. In CDCl_3_ Lorentz–Gauss transformation assisted assignment of the 50% labelled signals, but the 25% labelled signals could not be located reliably.

**Fig. 8:**
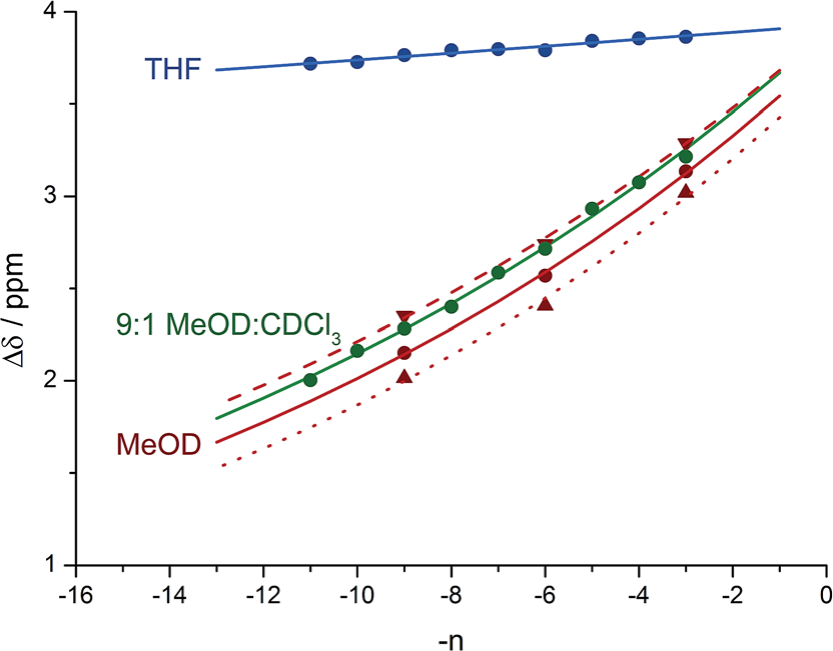
Anisochronicity in the labelled Aib** residues of **7a–c** in THF, ethanol (0 °C, 23 °C and 40 °C) and methanol. 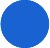 THF at 23 °C, 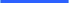 *f* = 0.005; 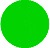 9 : 1 methanol : CDCl_3_ at 23 °C, 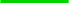 *f* = 0.054; 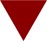 methanol at 0 °C, 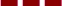 *f* = 0.055; 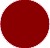 methanol at 23 °C, 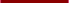 *f* = 0.061; 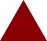 methanol at 40 °C, 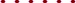 *f* = 0.065.

Fig. 8 shows a plot of anisochronicity at each of the positions in the chain against the position (–*n*) of the label in the chain relative to the C-terminal alaninamide (residue *i*). Fitting an exponential decay (eqn (3), using positive values of *n*) to each plot gives signal decay constants *f* (the per-residue decrease in Δ*δ*) of 6.1% in methanol, 5.4% in 9 : 1 methanol : chloroform, and 0.5% in THF. Earlier work had shown that aggregation effects may become important in solvents such as chloroform,^69^ but these were shown to be negligible in this case as the anisochronicity was independent of concentration over a 4-fold concentration range (4.6–18 μM). Changes in temperature induced a small variation in the value of *f* for the oligomers in methanol, which was 5.5% at 0 °C; 6.1% at 23 °C and 6.5% at 40 °C. These values correspond to an enthalpy difference of about 3 kJ mol^–1^ and an entropy difference of –13 J mol^–1^ K^–1^.

**Fig. 9:**
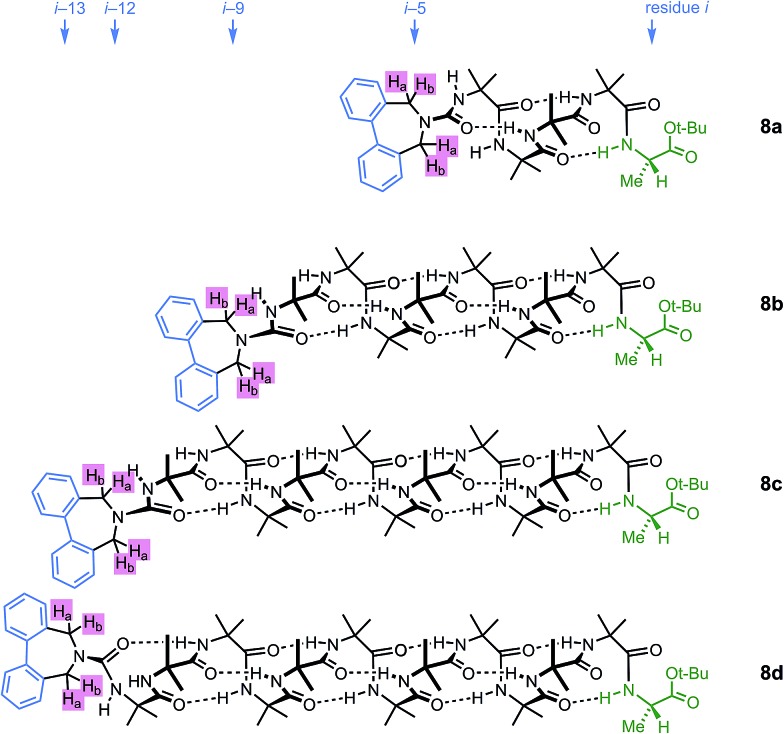
Dibenzazepinocarbonyl-capped oligomers with screw-sense induction from a C terminal l-Ala residue.

**Fig. 10:**
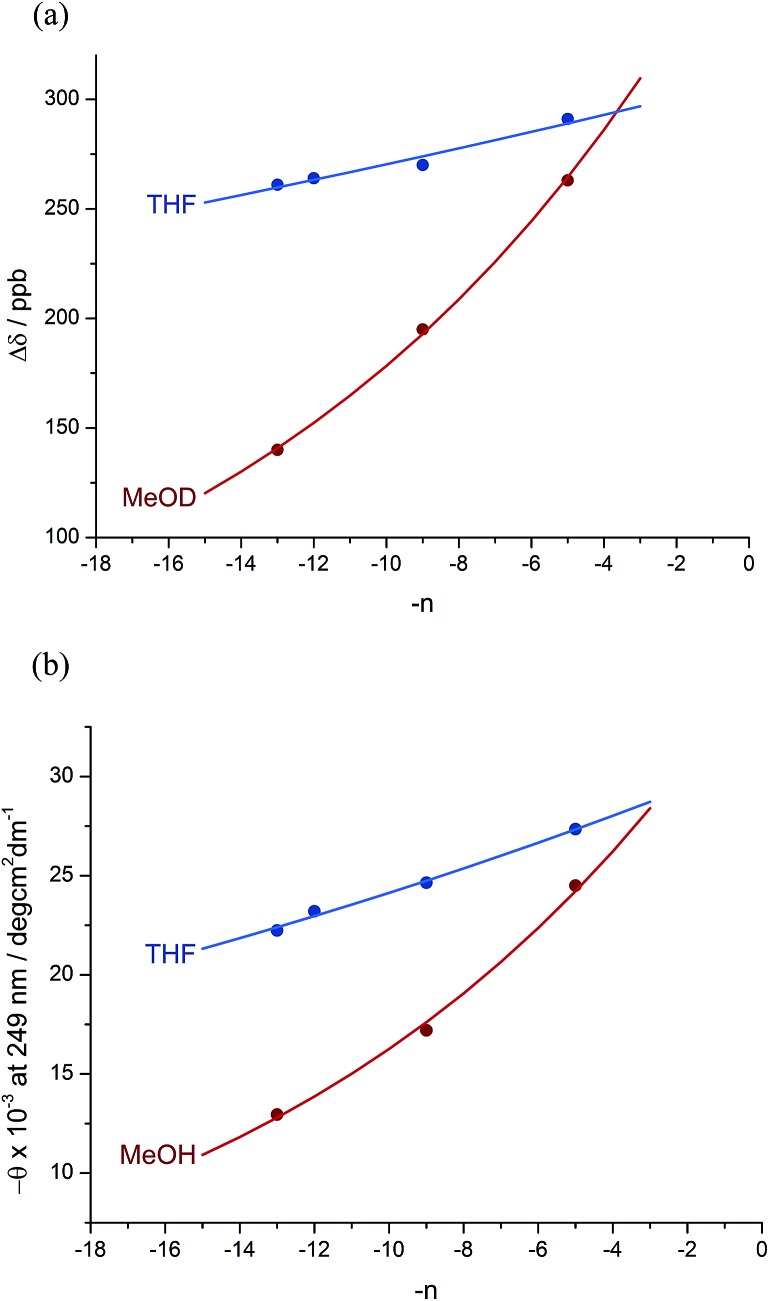
Effect of chain length *n* on characteristic features of the products **8a–d**. (a) Exponential decay of anisochronicity in the azepine CH_2_ groups with chain length (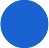 in THF at 23 °C; 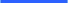 *f* = 0.013; 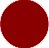 in methanol at 23 °C; 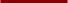 *f* = 0.077); (b) exponential decay of molar ellipticity at 249 nm (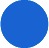 in THF at 23 °C; 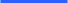 *f* = 0.025; 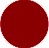 in methanol at 23 °C; 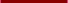 *f* = 0.076).

A further group of compounds **8a–d** (Fig. 9), developed as part of a related project,^70^ had a better solubility profile than **7**, and allowed comparisons of induced helical excess through four, eight, eleven or twelve achiral residues in methanol, chloroform and THF, as well as correlation with circular dichroism data. ^1^H NMR chemical shift separations Δ*δ* between *H*_a_ and *H*_b_ at the two equivalent methylene groups of the azepine rings in **8a–d** compounds at +23 °C are shown plotted against –*n* in Fig. 10, and an exponential decay (eqn (3)) fitted to these separations gave signal decay constants *f* of 1.3% in THF and 7.7% in MeOH. In CDCl_3_ the signals unfortunately showed no anisochronicity.

In the circular dichroism spectra of **8a–d** the chromophore associated with the azepine biaryl (*θ*_max_ at 249 nm) is well separated from those associated with the carbonyl groups in the chain (*θ*_max_ < 220 nm), and thus we may expect molar ellipticity at 249 nm to be proportional^71^ to the induced helical excess at the N terminus of the chain. Fig. 10b shows a plot of this value against chain length. From a modification of eqn (3)

5 *θ*_*n*_ = *θ*_*i*_ × (1 – *f*)^*n*^

we deduce signal decay constants *f* of 2.5% in THF and 7.6% in MeOH, at +23 °C.

## Discussion

There are numerous polymeric structures that are helical even though the individual monomers are achiral.^2,72–81^ Examples include not only oligomers of Aib,^27,29^ but also polyacetylenes,^82–84^ polyisocyanides,^85,86^ polyisocyanates,^87,88^ polyaromatic structures of various types^60,73,89–91^ and even polyacetals.^92,93^ The conformations of such helices consist of domains of identical screw-sense which may span the whole length of the oligomer, or alternatively may be interrupted by ‘helical perversions’:^94^ locations where the screw sense reverses.^95^ The lengths of the resulting domains of screw-sense uniformity have in some cases been quantified, and in one well studied case, the polyisocyanates, this length can be some 600 monomers at 20 °C.^87^ In other words, the average number of monomers separating one screw-sense reversal from the next is 600.

The decay of screw-sense preference on lengthening a helical domain interposed between a screw-sense controller and reporter may be viewed in terms of the chance of helix reversals occurring in that domain. A domain containing a single reversal, or indeed any odd number of reversals, will give rise to opposite screw-sense preferences at the two termini of the domain, while zero or an even number of reversals will lead to identical screw senses at the termini. Thus a helix having *P* screw sense at one terminus will have *P* screw sense at the other if it contains zero or an even number of reversals and *M* screw sense if it contains an odd number of reversals. The likelihood of the two ends of the domain having the same screw sense may be calculated by considering the excess probability of an odd number over an even number of screw sense reversals, assuming an equal probability of screw-sense reversal at each location in the domain.

For a domain of *N* elements, the difference in probability between having an even number of elements in state α and an odd number in state α is^65^

6 (2*p* – 1)^*N*^

where the probability of state α at each element is (1 – *p*). Since helical excess (he) = ([*P*] – [*M*])/([*P*] + [*M*]), interpreting state α as a helix reversal allows the inference that he at position *i* in a helix built from achiral monomers is related to he at position *i* + *n* by this same expression

7 he_*n*_ = he_*i*_ × (2*p* – 1)^*n*^

where *p* may be interpreted as the ‘fidelity’ with which a screw sense preference is transmitted from one monomer to the next (*p* = 1 implies complete communication of screw sense; *p* = 0.5 implies complete loss of screw sense control, and *p* = 0 implies complete inversion of screw sense).

Thus the empirical values for the signal decay constant *f* derived from eqn (3)–(5) may be interpreted structurally as a measure of the probability of screw-sense reversal, with *f* = 2 × (1 – *p*). In other words with *p* as the screw-sense fidelity, *f*/2 may be interpreted as the probability of a helix reversal occurring, and *f*, the signal decay constant, as the fractional decrease in helical excess as the screw-sense preference passes from one monomer to the next. Values for decay constant *f* and hence fidelity *p* obtained from Fig. 2, 4, 8 and 10 are assembled into Table 1.

**Table 1:** Values for signal decay constant *f*, screw-sense fidelity *p*, and helix persistence length *L* for compounds in the paper in various solvents

Compound series	Monomer	Solvent	*T*/°C	Per-residue decrease in he *f* ^*a*^/%	Screw-sense fidelity *p*	Average length of uniform screw sense *L*
**2**	Aib/Ac6c	THF	–50	0.8	0.996	
**2**	Aib/Ac6c	THF	+23	0.1	0.9996	
**7**	Aib	THF	+23	0.5	0.9975	200
**8**	Aib	THF	+23	1.3	0.993	
**8**	Aib	THF	+23	2.5^*b*^	0.988	
**2**	Aib/Ac6c	EtOH	+23	1.9	0.991	50
**2**	Aib/Ac6c	EtOH	+40	2.5	0.987	
**7**	Aib	9 : 1 MeOH : CDCl_3_	+23	5.4	0.973	18
**7**	Aib	MeOH	0	5.5	0.972	18
**7**	Aib	MeOH	+23	6.1	0.970	16
**7**	Aib	MeOH	+40	6.5	0.967	15
**8**	Aib	MeOH	+23	7.7	0.962	
**8**	Aib	MeOH	+23	7.6^*b*^	0.962	
**3**	Gly	THF	+23	13	0.935	—
**3**	Gly	EtOH	+23	31	0.845	—
**3**	Gly	MeOH	+23	49	0.76	—

^*a*^Determined by NMR unless otherwise stated.

^*b*^Determined by circular dichroism.

This interpretation of the decay of he along a chain in terms of the likelihood of the two termini of a helical domain having the same or opposite screw sense makes it clear that the decay constant *f* for any monomer must be independent of whether the ‘signal’ is passing from the N to the C (as in **2** and **3**) or the C to the N (as in **7** and **8**) terminus. The values of *p* obtained for Aib in MeOH from **7** or **8** for a signal travelling from the C terminus to the N terminus (*p* = 0.970, 0.962, 0.962) are close to values previously calculated for a signal travelling from the N terminus to the C terminus (0.9735 (ref. 31,34,96), 0.965 (ref. 65)). It is also notable that the value of *p* obtained for Gly from **3** corresponds closely to a previous observation^17,97^ that Gly conducts a helical preference 59% as efficiently as Aib (*i.e.* (1 – *f* ^Gly^)/(1 – *f* ^Aib^) = 0.59; the corresponding value calculated from the data in Table 1 is 0.51/0.939 = 0.54).

The values in Table 1 clearly show that helical reversals are more likely at higher temperatures, and in more polar solvents. It may be deduced that whatever disruption of the hydrogen-bonding network is required to allow a helix reversal is more favourable in polar than non-polar solvents. The question of what structures these helix reversals adopt is still under investigation, but one important observation suggests that helix reversal does not require the formation of an unstructured random coil conformation. In such a conformation, oligomer NH groups would be exposed to the deuterated solvent and would therefore undergo hydrogen–deuterium exchange.^98,99^ Yet both the observed rate at which the NH signals of the oligomer exchange with D from the deuterated solvent, and the rate at which protonated signals returned on dissolution of deuterated oligomer in protonated solvent, are extremely slow. H/D exchange in CD_3_OD occurs on a time scale of hours to days at ambient temperature, while helical inversion occurs on the millisecond time scale,^29,34,62–64^ suggesting that random coil conformations make a negligible contribution to the ensemble of structures adopted by Aib oligomers. Thus we propose that the helical reversals that lead to signal decay are well-defined, local structural features which entail only a small perturbation of the 3_10_ helical structure, and retain a well-defined intramolecularly hydrogen-bonded network. We are currently exploring experimentally the possible detailed structures for these helix reversals. As suggested by Kubasik,^63^ migration of a well-structured helical reversal along a chain, sequentially breaking and reforming only one or maybe two hydrogen bonds at a time, could provide a mechanism for the remarkably rapid inversion of a 3_10_ helix.

The values also allow for the first time an estimate to be made, using the one-dimensional Ising model,^95,100^ of the average length over which hypothetical extended Aib homo-polymers might be expected to adopt a uniform screw sense. The Ising model, first used to describe ferromagnetic and antiferromagnetic arrays of atoms, assumes that the energetic cost of a state reversal (here a change in screw sense) in a linear array is determined solely by nearest neighbours. If the chance of a helix reversal at any one residue is (1 – *p*), the Boltzmann relation gives

8 (1 – *p*)/*p* = e^–2*J*/*kT*^

where 2*J* is the energetic penalty for introducing a helix reversal.

According to the one-dimensional Ising model, the helical excess will decay exponentially over *n* segments as the nth power of tanh(*J*/*kT*). This factor is equivalent to our empirical decay factor (1 – *f*) (eqn (3)), giving as expected *f* = 2 × (1 – *p*). The persistence length *L* (the average distance between reversals) may be obtained from the Ising model by^101^

9 *L* = –1/ln[tanh(*J*/*kT*)] = –1/ln(2*p* – 1) = –1/ln(1 – *f*)

Values for *L* are shown in Table 1 for selected data. A sufficiently soluble Aib polymer, in a non-polar solvent, can evidently be expected to maintain a preferred screw sense over distances measurable in hundreds of monomers.

One advantage of the Ising model is that it allows a simple estimate, from eqn (8), of the energetic cost 2*J* of a helix reversal. For example, in the case of **2a–d** in THF at –50 °C (Fig. 2a), *J* = 10 kJ mol^–1^; for **7a–c** in methanol at +40 °C it is 9 kJ mol^–1^, both considerably less than the typical strength of a hydrogen bond. This is consistent with the observation noted earlier that hydrogen–deuterium exchange is very slow in such systems, providing strong evidence that helix inversion simply involves a rearrangement of intramolecular hydrogen bonds rather than any reduction in their number. The Ising model assumes that the driving force for the retention of helicity is purely energetic; as the analysis of the temperature dependence of *f* for **7a–c** in methanol in the preceding section shows, a substantial part of the driving force is in fact entropic.

## Conclusion

A screw-sense preference induced by a chiral residue at the terminus of an achiral helical oligomer may be considered as a signal whose strength decays as with decreasing distance from the chiral inducer. The spatial decay rate may be quantified by means of NMR or CD probes of helical excess located at successive positions in an oligomeric structure, or located at the terminus of oligomers of varying lengths. The decay rate depends on solvent and temperature (signal decay is faster at higher temperature and in more polar solvents) and on the nature of the achiral oligomers in the chain (greater flexibility gives rise to faster signal decay). Typical values are of the order of 5% for Aib in MeOH, and 1% for Aib in THF, leading to helix persistence lengths in non-polar solvents of around 200 monomers. The decay rate may be interpreted as the result of a helix reversal occurring randomly at any position in the oligomer. Helix reversals appear to be localized, well-ordered structures, and ongoing work seeks to establish their detailed geometry.
