# Supplementary material for: Flaws in foldamers: conformational uniformity and signal decay in achiral helical peptide oligomers
Source: Chem Sci. 2015 Jan 21;6(4):2313–22. doi: 10.1039/c4sc03944k (PMC5645781; doi:10.1039/c4sc03944k)
Supplement: Supplementary file 1 [file SC-006-C4SC03944K-s001.pdf]

Electronic Supplementary Material (ESI) for Chemical Science.
This journal is © The Royal Society of Chemistry 2015

Flaws in foldamers: screw-sense fidelity and signal decay in achiral helical peptide oligomers

*Bryden A. F. Le Bailly, Liam Byrne, Vincent Diemer, Mohammadali Foroozandeh, Gareth A. Morris, and Jonathan Clayden**

School of Chemistry, University of Manchester, Oxford Rd, Manchester M13 9PL, UK

Supporting Information

Table of Contents

Experimental Section S3

General Experimental and Materials S3

Instrumentation S3

Synthetic Procedures S4

Example spectra showing ^{13}C-labelled Aib** of **7a** S15

Calculation of decay rate *f* S16

Details of NMR experiment used to resolve glycines in compound **3** S16

NMR Spectra S19

References S39

General Experimental and Materials

All reactions were carried out in oven-dried glassware under an atmosphere of nitrogen using standard anhydrous techniques. All reagents were obtained from commercially available sources and used without further purification, or where indicated prepared internally. Air- and moisture-sensitive liquids and solutions were transferred *via* syringe or stainless steel cannula. Reactions performed at 0 °C were done so using an ice bath; those performed at -78 °C were done so using an acetone/dry ice bath. Anhydrous dichloromethane was obtained by distillation from calcium hydride. Other anhydrous reaction solvents were obtained from standard anhydrous solvent engineering system. Triethylamine was stored over potassium hydroxide. All products were dried on a rotary evaporator followed by connection to a high vacuum system to remove any residual solvent. Flash chromatography was performed on silica gel (Merck 60H, 40-60 nm, 230 – 300 mesh). Analytical thin layer chromatography was performed on aluminium backed silica (60 F_{254}) plates.

Instrumentation

All ^{1}H and ^{13}C nuclear magnetic resonance spectra were obtained using Bruker AVANCE 300, 400 or 500 MHz spectrometers. Chemical shifts are quoted in parts per million (ppm), and coupling constants (*J*) are quoted in Hz to the nearest 0.5 Hz. ^{1}H-NMR spectra were referenced to the residual deuterated solvent peak ($CDCl_3$ 7.27; CD_3OD 3.31; CD_3CD_2OD 3.56; THF-d_8 1.73 ppm) and ^{13}C-NMR were referenced to the carbon resonance of the solvent ($CDCl_3$ 77.00; CD_3OD 49.05; CD_3CD_2OD 17.31; THF-d_8 25.37 ppm). Multiplicities are denoted as s (singlet), d (doublet), t (triplet), q (quartet), spt (septet) and m (multiplet) or denoted as br (broad), or some combination of these, where appropriate. Where ^{1}H-NMR spectra were run in CD_3OD, D_2O or CD_3CD_2OD exchangeable protons (NH, OH) are reported only where observed.

Infra-red spectra were recorded on an ATi Perkin Elmer Spectrum RX1 FT-IR spectrometer. Only absorption maxima (λ_{max}) of interest are reported and quoted in wavenumbers (cm^{-1}). Low and high resolution mass spectra were recorded by staff at the University of Manchester. Electrospray (ES) spectra were recorded on a Waters Platform II and high resolution mass spectra (HRMS) were recorded on a Thermo Finnigan MAT95XP and are accurate to ± 0.001 Da. Melting points were determined on a GallenKamp apparatus and are uncorrected. Optical rotation measurements were taken on an AA-100 polarimeter at 20 °C with the solvent and concentration stated. Circular Dichroism (CD) measurements were performed at 20 °C on a JASCO J-815 spectropolarimeter, using a 1 mm cell with the solvent and concentration stated, where applicable.

Methods for the synthesis of HCl.H-Aib**-OMe,[1] N_3-AibOH, Azib-Cl, H-AlaNH^{t}Bu, H-Aib$_2$-O^{t}Bu, H-Aib$_3$-O^{t}Bu, H-Aib$_4$-O^{t}Bu,[2] N_3-Aib$_3$-OH,[3] **1a-1d** and **2a-2d**[4] and **3**[2] have been reported previously. The synthesis of compounds **8a** and **8b** will be reported in a future communication.

Synthetic Procedures

Aze-Aib$_{11}$AlaNH^{t}Bu 8c

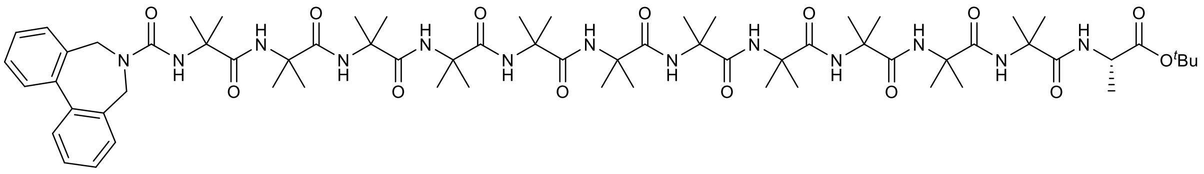

^{1}H-NMR (400 MHz, $CDCl_3$) δ_H 8.40 (1H, br, NH), 7.82 (1H, br s, NH), 7.79 (1H, br s, NH), 7.77 (1H, br s, NH), 7.75 (2H, br s, NH x2), 7.69 (1H, br s, NH), 7.67 (1H, br s, NH), 7.57 (1H, br s, NH), 7.55 (2H, s, ArCH x2), 7.51 (3H, m, ArCH x3), 7.41 (3H, m, ArCH x3), 7.40 (1H, br s, NH), 6.71 (1H, br s, NH), 5.68 (1H, br s, NH), 4.28 (5H, m, CH_2 x2 and CH), 1.55 (12H, m, CH_3 x4), 1.47-1.53 (45H, m, CH_3 x 15), 1.45 (3H, s, CH_3), 1.43 (12H, s, CH_3 and $C(CH_3)_3$), 1.40 (3H, m, C*H*$_3$-CH), 1.37 (3H, s, CH_3). ^{13}C-NMR (101 MHz, $CDCl_3$) δ_C 176.2 (CO), 176.1 (CO), 176.1 (CO), 176.1 (CO), 176.0 (CO), 175.9 (CO), 175.7 (CO), 175.5 (CO), 175.4 (CO), 175.2 (CO), 174.7 (CO), 172.6 (CO), 156.5 (CON), 140.4 (ArC), 133.8 (ArC), 129.3 (ArCH), 128.9 (ArCH), 128.5 (ArCH), 128.4 (ArCH), 80.4 (CMe_3), 57.3 ($^{\alpha}$C), 56.8 ($^{\alpha}$C), 56.7 ($^{\alpha}$C), 56.7 ($^{\alpha}$C), 56.6 ($^{\alpha}$C), 56.5 ($^{\alpha}$C), 56.5 ($^{\alpha}$C x4), 56.4 ($^{\alpha}$C), 49.5 ($^{\alpha}$CH), 48.1 (br, CH_2Ar), 28.0 (C(*C*H$_3$)$_3$), 23.0-27.4 (br, CH_3 x24), 16.9 (*C*H$_3$-CH). IR (neat) ν_{max}/cm^{-1} = 3273, 2984, 2935, 1738, 1650, 1538, 1455, 1383, 1361. MS (ES$^+$, CH_2Cl_2) 1303.1 (95%, [(M+H]$^+$), 1325.1 (100%, [M+Na]$^+$). Mp 240-242 °C. $[\alpha]_D^{20}$ = −20.0 (*c* 0.5, CH_2Cl_2).

Aze-Aib$_{11}$AlaNH^{t}Bu 8d

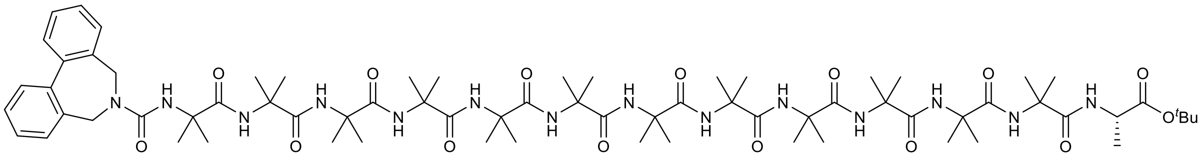

^{1}H-NMR (400 MHz, $CDCl_3$) δ_H 8.39 (1H, br s, NH), 7.82 (1H, br s, NH), 7.80 (1H, br s, NH), 7.76 (4H, m, NH x4), 7.69 (1H, br s, NH), 7.67 (1H, br s, NH), 7.57 (1H, br s, NH), 7.55 (2H, s, ArCH x2), 7.51 (3H, m, ArCH x3), 7.41 (3H, s, ArCH x3), 7.40 (1H, br s, NH), 6.71 (1H, br s, NH), 5.63 (1H, br s, NH), 4.27 (5H, m, CH_2 x2 and CH), 1.56 (9H, m, CH_3 x3), 1.47-1.54 (54H, m, CH_3 x18), 1.45 (3H, s, CH_3), 1.43 (12H, s, CH_3 and $C(CH_3)_3$), 1.41 (1H, m, C*H*$_3$-CH), 1.38 (3H, s, CH_3). ^{13}C-NMR (101 MHz, $CDCl_3$) δ_C 176.2 (CO x2), 176.2 (CO), 176.1 (CO), 176.1 (CO), 176.0 (CO), 175.9 (CO), 175.7 (CO), 175.5 (CO), 175.4 (CO), 175.2 (CO), 174.8 (CO), 172.7 (CO), 156.5 (CO), 140.4 (ArC), 133.7 (ArC), 129.3 (ArCH), 129.0 (ArCH), 128.5 (ArCH), 128.4 (ArCH), 80.4 (CMe_3), 57.4 ($^{\alpha}$C), 56.8 ($^{\alpha}$C), 56.7 ($^{\alpha}$C), 56.7 ($^{\alpha}$C), 56.6 ($^{\alpha}$C), 56.5 ($^{\alpha}$C x6), 56.4 ($^{\alpha}$C), 49.5 ($^{\alpha}$CH), 48.1 (CH_2 x2), 28.0 (C(*C*H$_3$)$_3$), 22.6-27.4 (CH_3 x24), 16.9 (*C*H$_3$-CH). IR (neat) ν_{max}/cm^{-1} = 3291, 2984, 2931, 1651, 1531, 1455, 1384, 1362. MS (ES$^+$, CH_2Cl_2) 1388.4 (80%, [(M+H]$^+$), 1411.1 (100%, [M+Na]$^+$). Mp 283-285 °C. $[\alpha]_D^{20}$ = −21.6 (*c* 0.5, CH_2Cl_2).

N_3-AibAib-OMe**

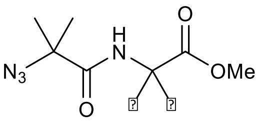

HCl.N_3-Aib-OMe** (1.91 g, 12.27 mmol) and triethylamine (4.28 mL, 30.69 mmol) were dissolved in CH_2Cl_2 (30 mL) and the resulting mixture cooled to 0 °C. A freshly prepared batch of Azib-Cl (2.55 g, 17.30 mmol) was added dropwise and the reaction mixture was allowed to warm to room temperature and stirred for 16 h. EtOAc (60 mL) was added and the solution washed with $KHSO_4$ (10%, 2 x 15 mL), $NaHCO_3$ (sat., 2 x 15 mL) and brine (10 mL). The organic phase was then dried ($MgSO_4$), filtered and concentrated *in vacuo*. The crude reaction product was isolated by column chromatography (3:1 PE:EtOAc) to give **N_3-AibAib**-OMe** (2.52 g, 89%) as a pale yellow oil. ^{1}H-NMR (400 MHz, $CDCl_3$) δ_H 6.98 (1H, br s, NH), 3.74 (3H, s, OCH_3), 1.55 (6H, dd, *J*=129.0, 4.5, *CH_3 x2), 1.53 6H, s, CH_3 x2). ^{13}C-NMR (101 MHz, $CDCl_3$) δ_C 174.6 (CO), 171.6 (CO), 64.2 (◦C), 52.6 (OCH_3), 24.7 (*CH_3), 24.3 (CH_3). The triplet signal arising from the carbon alpha to the ^{13}C-labels could not be located. IR (neat) ν_{max}/cm^{-1} = 3379, 2979, 2109, 1740, 1674, 1511, 1455. MS (ES^+, MeOH) 231.2 (100%, $[(M+H]^+$), 253.1 (40%, $[M+Na]^+$).

H-AibAib-OMe**

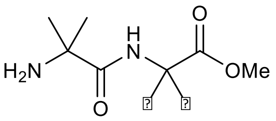

N_3-AibAib-OMe** (2.52 g, 10.96 mmol) was dissolved in MeOH (25 mL) and Pd/C (10%, 750 mg) was added under an atmosphere of N_2. An atmosphere of H_2 was introduced *via* a vacuum/H_2 purge cycle (3 x) and the reaction mixture stirred for 16 h. The Pd/C was filtered off with celite and the solvent removed *in vacuo* to give **H-AibAib**-OMe** (2.24 g, >99%) as a waxy white solid which was used without further purification. Note: Product was used as soon as possible to prevent cyclisation to the diketopiperazine. ^{1}H-NMR (300 MHz, $CDCl_3$) δ_H 7.85 (1H, br s, NH), 3.74 (3H, s, OCH_3), 1.55 (6H, dd, *J*=129.5, 4.5, *CH_3 x2), 1.46 (6H, s, CH_3 x2). ^{13}C-NMR (101 MHz, $CDCl_3$) δ_C 174.9 (CO), 170.5 (CO), 58.1 (◦C), 52.9 (OCH_3), 24.8 (CH_3), 24.6 (*CH_3). The triplet signal arising from the carbon alpha to the ^{13}C-labels could not be located. IR (neat) ν_{max}/cm^{-1} = 2965, 1663, 1490, 1444. HRMS (ES^+, CH_2Cl_2) Calc. for $C_7{}^{13}C_2H_{19}N_2O_3$ ($[M+H]^+$) 205.1463, found 205.1471.

N_3-Aib_2Aib-OMe**

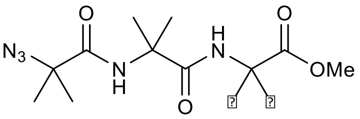

H-AibAib-OMe** (BL200, 2.24 g, 10.96 mmol) and triethylamine (2.3 mL, 16.40 mmol) were dissolved in CH_2Cl_2 (30 mL) and the resulting mixture cooled to 0 °C. A freshly prepared batch of Azib-Cl (2.43 g, 16.44 mmol) was added dropwise and the reaction mixture was allowed to warm to room temperature and stirred for 16 h. EtOAc (90 mL) was added and the solution washed with $KHSO_4$ (5%, 2 x 20 mL), $NaHCO_3$ (sat., 2 x 20 mL) and brine (20 mL). The organic phase was then dried

($MgSO_4$), filtered and concentrated *in vacuo*. The crude reaction product was isolated by column chromatography (twice; 4:1 to 1:1 PE:EtOAc) to give **N_3-Aib$_2$Aib**-OMe** (1.68 g, 49%) as an off-white solid. ^{1}H-NMR (400 MHz, $CDCl_3$) δ_H 7.08 (1H, br s, NH), 7.05 (1H, br s, NH), 3.75 (3H, s, OCH_3), 1.56 (6H, s, CH_3 x2), 1.55 (6H, dd, *J*=129.5, 4.5, *CH_3 x2), 1.54 (6H, s, CH_3 x2). ^{13}C-NMR (101 MHz, $CDCl_3$) δ_C 175.2 (CO), 173.1 (CO), 172.1 (CO), 64.3 ($^\alpha$C), 57.2 ($^\alpha$C), 56.6 (t, *J*=36.5, $^\alpha$*C*-(*CH_3)$_2$), 52.7 (OCH_3), 24.9 (CH_3), 24.4 (*CH_3), 24.3 (CH_3). IR (neat) ν_{max}/cm^{-1} = 3402, 3366, 2989, 2117, 1719, 1677, 1663, 1502, 1464, 1436. HRMS (ES^+, CH_2Cl_2) Calc. for $C_{11}{}^{13}C_2H_{24}N_5O_4$ ($[M+H]^+$) 316.1890, found 316.1888. Mp 88-90 °C.

H-Aib$_2$Aib-OMe**

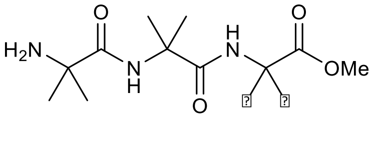

N_3-Aib$_2$Aib-OMe** (663 mg, 2.1 mmol) was dissolved in MeOH (20 mL) and Pd/C (10%, 70 mg) was added under an atmosphere of N_2. An atmosphere of H_2 was introduced *via* a vacuum/H_2 purge cycle (3 x) and the reaction mixture stirred for 16 h. The Pd/C was filtered off with celite and the solvents removed *in vacuo* to give **H-Aib$_2$Aib**-OMe** (609 mg, >99%) as a white solid. ^{1}H-NMR (400 MHz, $CDCl_3$) δ_H 8.16 (1H, br s, NH), 7.71 (1H, br s, NH), 3.72 (3H, s, OCH_3), 1.53 (6H, s, CH_3 x2), 1.52 (6H, dd, *J*=129.5, 4.5, *CH_3 x2), 1.36 (6H, s, CH_3 x2). ^{13}C-NMR (101 MHz, $CDCl_3$) δ_C 178.1 (CO), 175.2 (CO), 173.7 (CO), 57.0 ($^\alpha$C), 55.0 ($^\alpha$C), 52.5 (OCH_3), 28.9 (CH_3), 25.1 (CH_3), 24.63 (*CH_3). The triplet signal arising from the carbon alpha to the ^{13}C-labels could not be located. IR (neat) ν_{max}/cm^{-1} = 3313, 3282, 2979, 1784, 1641, 1493, 1454. HRMS (ES^+, CH_2Cl_2) Calc. for $C_{11}{}^{13}C_2H_{26}N_3O_4$ ($[M+H]^+$) 290.1985, found 290.1980. Mp 143-145 °C.

N_3-Aib$_2$Aib-OH 4****

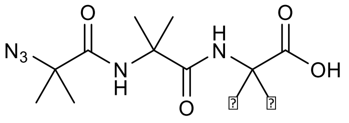

N_3-Aib$_2$Aib-OMe** (994 mg, 3.15 mmol) and LiOH (529 mg, 22 mmol) were dissolved in THF/H_2O (4:1, 30 mL total) and the resulting mixture was heated at 40 °C for 16 h. The reaction mixture was allowed to cool to room temperature and HCl (1 M) was added until the pH=1. The aqueous phase was extracted with EtOAc (100 mL) and the organic phase was then washed with HCl (1 M, 20 mL), brine (20 mL) and was dried (Na_2SO_4), filtered and concentrated *in vacuo* to give **N_3-Aib$_2$Aib**-OH 4**** (949 mg, >99%) as a white solid. ^{1}H-NMR (400 MHz, $CDCl_3$) δ_H 7.23 (1H, br s, NH), 7.01 (1H, br s, NH), 1.57 (6H, s, CH_3 x2), 1.56 (6H, dd, *J*=130.0, 4.5,*CH_3 x2), 1.53 (6H, s, CH_3 x2). ^{13}C-NMR (101 MHz, $CDCl_3$) δ_C 177.3 (CO), 174.1 (CO), 172.9 (CO), 64.3 ($^\alpha$C), 57.3 ($^\alpha$C), 24.9 (CH_3), 24.6 (*CH_3), 24.3 (CH_3). The triplet signal arising from the carbon alpha to the ^{13}C-labels could not be located. IR (neat) ν_{max}/cm^{-1} = 3353, 2977, 2934, 2107, 1710, 1683, 1535, 1498, 1535, 1498, 1473, 1452, 1409. HRMS (ES^+, CH_2Cl_2) Calc. for $C_{10}{}^{13}C_2H_{25}N_2O_5Na$ ($[M+Na]^+$) 302.1723, found 302.1729. Mp 114-116 °C.

N_3-Aib$_2$Aib$^{50\%}$Aib$_2$Aib**-OMe**

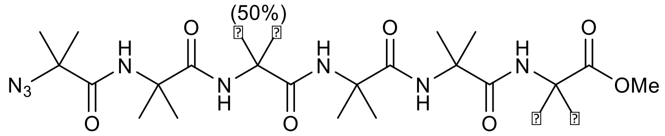

N_3-Aib$_2$AibOH 4**** (243 mg, 0.81 mmol) and **N_3-Aib$_3$-OH 4** (241 mg, 0.81 mmol) were dissolved in CH_2Cl_2 and the resulting solution cooled to 0 °C. *N*-(3-Dimethylaminopropyl)-*N*′-ethylcarbodiimide (0.34 mL, 1.93 mmol) was added, the reaction mixture was allowed to warm to room temperature and stirred for 3 h. The solution was then concentrated *in vacuo*, EtOAc (30 mL) was added and the organic phase washed with $KHSO_4$ (10%, 3 x 7 mL), brine (7 mL), dried ($MgSO_4$), filtered and concentrated *in vacuo.* The crude azlactone was then place under high vacuum (<0.1 mbar) before being dissolved in MeCN (10 mL) with **H-Aib$_2$Aib**-OMe** (513 mg, 1.77 mmol) and the resulting mixture was heated to reflux for 5 d. The solvent was removed *in vacuo*, EtOAc (40 mL) was added and the organic phase washed with $KHSO_4$ (10%, 2 x 8 mL), $NaHCO_3$ (2 x 8 mL), brine (8 mL), dried ($MgSO_4$), filtered and concentrated to give **N_3-Aib$_2$Aib$^{50\%}$**Aib$_2$Aib**-OMe** (831 mg, 90%) as a white solid. ^{1}H-NMR (500 MHz, $CDCl_3$) δ_H 7.39 (1H, br s, NH), 7.37 (1H, br s, NH), 7.21 (1H, br s, NH), 6.89 (1H, br s, NH), 6.10 (1H, br s, NH), 3.69 (3H, s, OCH_3), 1.56 (6H, s, CH_3 x2), 1.52 (6H, dd, *J*=129.0, 4.5, *CH_3 x2 (100%)), 1.50 (12H, s, CH_3 x4), 1.47 (6H, s, CH_3 x2), 1.43 (3H, s, CH_3 x2 (50%)), 1.43 (3H, dd, *J*=129.0, 4.5, *CH_3 x2 (50%)). ^{13}C-NMR (126 MHz, $CDCl_3$) δ_C 175.5 (CO), 174.5 (CO), 173.6 (CO), 173.5 (CO), 173.1 (CO), 172.7 (CO), 63.9 ($^\alpha$C), 57.0 ($^\alpha$C), 56.9 ($^\alpha$C), 56.7 ($^\alpha$C), 56.6 ($^\alpha$C), 52.0 (OCH_3), 25.3 (CH_3), 25.2 (*CH_3 (50%)), 24.9 (*CH_3 (100%)), 24.8 (CH_3), 24.3 (CH_3). The triplet signals arising from the carbons alpha to the ^{13}C-labels could not be located. IR (neat) ν_{max}/cm^{-1} = 3324, 2983, 2936, 2110, 1729, 1668, 1645, 1525, 1454. HRMS (ES$^+$, MeOH) Calc. for $C_{23}{}^{13}C_2H_{44}N_7O_8Na$ ([M+Na]$^+$) 593.3298, found 593.3280; calc. for $C_{21}{}^{13}C_4H_{45}N_7O_8$ ([M+H]$^+$) 573.3540, found 573.3524. Mp 223-225 °C.

H-Aib$_2$Aib$^{50\%}$Aib$_2$Aib**-OMe**

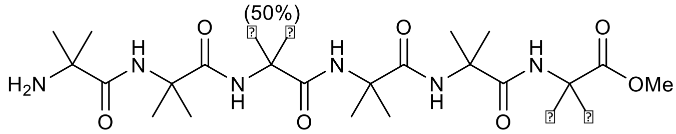

N_3-Aib$_2$Aib$^{50\%}$Aib$_2$Aib**-OMe** (824 mg, 1.44 mmol) was dissolved in MeOH (15 mL) and Pd/C (10%, 85 mg) was added under an atmosphere of N_2. An atmosphere of H_2 was introduced *via* a vacuum/H_2 purge cycle (3 x) and the reaction mixture stirred for 16 h. The Pd/C was filtered off with celite and the solvents removed *in vacuo* to give **H-Aib$_2$Aib$^{50\%}$**Aib$_2$Aib**-OMe** (782 mg, >99%) as a white solid. ^{1}H-NMR (300 MHz, CD_3OD) δ_H 7.86 (1H, br s, NH), 7.76(1H, br s, NH), , 3.66 (3H, s, OCH_3), 1.57 (6H, s, CH_3 x2), 1.49 (6H, dd, *J*=129.0, 4.5, *CH_3 x2), 1.47 (12H, s, CH_3 x4), 1.46 (6H, s, CH_3 x2), 1.42 (3H, s, CH_3 x2 (50%)), 1.42 (3H, dd, *J*=129.0, 4.0, CH_3 x2 (50%)). ^{13}C-NMR (75 MHz, CD_3OD) δ_C 177.0 (CO), 177.0 (CO), 176.9 (CO), 176.9 (CO), 176.2 (CO), 58.3 ($^\alpha$C), 58.1 ($^\alpha$C), 58.0 ($^\alpha$C), 57.9 ($^\alpha$C), 57.7 ($^\alpha$C), 52.7 (OCH_3), 25.8 (CH_3), 25.6 (CH_3), 25.4 (*CH_3), 25.3 (*CH_3, CH_3), 25.0 (CH_3). The triplet signals arising from the carbons alpha to the ^{13}C-labels could not be located. IR (neat) ν_{max}/cm^{-1} = 3307, 2983, 1729, 1657, 1527, 1456. HRMS (ES$^+$, CH_2Cl_2) Calc. for $C_{23}{}^{13}C_2H_{47}N_6O_7$ ([M+H]$^+$) 545.3568, found 545.3568; calc. for $C_{21}{}^{13}C_4H_{47}N_6O_7$ ([M+H]$^+$) 547.3635, found 547.3631. Mp 168-171 °C.

N_3-Aib$_2$Aib$^{25\%}$Aib$_2$Aib$^{50\%}$**Aib$_2$Aib**-OMe 5**

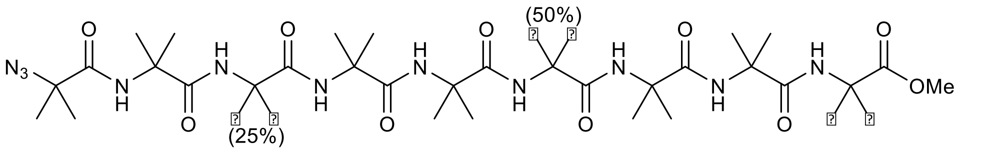

N_3-Aib$_2$AibOH 4**** (99 mg, 0.33 mmol) and **N_3-Aib$_3$-OH 4** (296 mg, 0.99 mmol) were dissolved in CH_2Cl_2 (10 mL) and the resulting solution cooled to 0 °C. *N*-(3-Dimethylaminopropyl)-*N*′-ethylcarbodiimide (0.28 mL, 1.58 mmol) was added, the reaction mixture was allowed to warm to room temperature and stirred for 3 h. The solution was then concentrated *in vacuo*, EtOAc (40 mL) was added and the organic phase washed with $KHSO_4$ (10%, 3 x 7 mL), brine (7 mL), dried ($MgSO_4$), filtered and concentrated *in vacuo.* The crude azlactone was then place under high vacuum (<0.1 mbar) before being dissolved in MeCN (6 mL) with **H-Aib$_2$Aib$^{50\%}$**Aib$_2$Aib**-OMe** (720 mg, 1.32 mmol) and the resulting mixture was heated to reflux for 5 d. The solvent was removed *in vacuo*, CH_2Cl_2 (50 mL) was added and the organic phase washed with $KHSO_4$ (10%, 2 x 7 mL), $NaHCO_3$ (2 x 7 mL), brine (7 mL), dried ($MgSO_4$), filtered and concentrated to give **N_3-Aib$_2$Aib$^{25\%}$**Aib$_2$Aib$^{50\%}$**Aib$_2$Aib**-OMe 5** (340 mg, 32%) as a white solid. ^{1}H-NMR (400 MHz, $CDCl_3$) δ_H 7.64 (1H, br s, NH), 7.59 (1H, br s, NH), 7.57 (1H, br s, NH), 7.52 (1H, br s, NH), 7.47 (1H, br s, NH), 7.36 (1H, br s, NH), 6.95 (1H, br s, NH), 6.16 (1H, br s, NH), 3.69 (3H, s, OCH_3), 1.57 (6H, s, CH_3 x2), 1.54 (6H, dd, *J*=129.5, 4.0, *CH_3 x2), 1.53 (6H, s, CH_3 x2), 1.51 (6H, s, CH_3 x2), 1.50 (6H, s, CH_3 x2), 1.49 (3H, s, CH_3 x2 (50%)), 1.49 (3H, dd, *J*=129.0, 4.0, *CH_3 x2 (50%)), 1.48 (6H, s, CH_3 x2), 1.47 (6H, s, CH_3 x2), 1.44 (4.5H, s, CH_3 x2 (75%)), 1.44 (1.5H, dd, *J*=129.5, 4.5, *CH_3 x2 (25%)). ^{13}C-NMR (101 MHz, $CDCl_3$) δ_C 176.2, 175.7 (CO), 175.6 (CO), 175.5 175.2 (CO), 175.1 (CO), 174.9 (CO), 174.2 (CO), 174.0 (CO), 173.3 (CO), 173.1 (CO), 64.0 (αC), 56.9 (αC), 56.8 (αC), 56.8 (αC), 56.6 (αC), 56.6 (αC), 56.6 (αC), 51.9 (OCH_3), 25.3 (CH_3), 25.0 (*CH_3 (100%, 50%, 25%) and CH_3 x3), 24.7 (CH_3), 24.7 (CH_3), 24.4 (CH_3), 24.3 (CH_3). The triplet signals arising from the carbons alpha to the ^{13}C-labels could not be located. IR (neat) ν_{max}/cm^{-1} = 3303, 2983, 2110, 1729, 1658, 1532, 1454. HRMS (ES$^+$, MeOH) Calc. for $C_{35}{}^{13}C_2H_{66}N_{11}O_{10}$ ($[M+H]^+$) 826.5056, found 826.5061; Calc. for $C_{33}{}^{13}C_4H_{66}N_{11}O_{10}$ ($[M+H]^+$) 828.5123, found 828.5128; Calc. for $C_{31}{}^{13}C_6H_{66}N_{11}O_{10}$ ($[M+H]^+$) 830.5190, found 830.5195. Mp 289-291°C.

N_3-Aib$_2$Aib$^{25\%}$Aib$_2$Aib$^{50\%}$**Aib$_2$Aib**-OH**

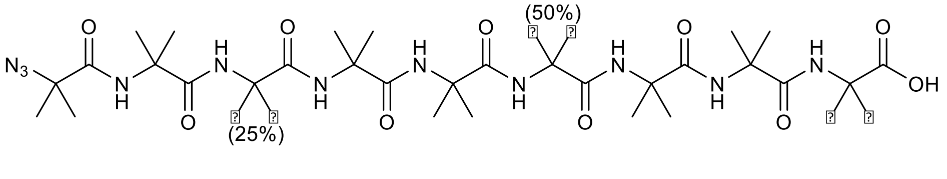

N_3-Aib$_2$Aib$^{25\%}$Aib$_2$Aib$^{50\%}$**Aib$_2$Aib**-OMe 5** (344 mg, 0.43 mmol) and LiOH (82 mg, 3.42 mmol) were dissolved in THF/H_2O (4:1, 5 mL total) and the resulting mixture was heated at 40 °C for 16 h. The reaction mixture was allowed to cool to room temperature and HCl (1 M) was added until the pH=1. The aqueous phase was extracted with CH_2Cl_2 (2x25 mL) and the organic phase was then washed with HCl (1 M, 10 mL), brine (10 mL) and was dried (Na_2SO_4), filtered and concentrated *in vacuo* to give **N_3-Aib$_2$Aib$^{25\%}$**Aib$_2$Aib$^{50\%}$**Aib$_2$Aib**-OH** (332 mg, 99%) as a white solid. ^{1}H-NMR (500 MHz, $CDCl_3$) δ_H 7.90 (1H, br s, NH), 7.77 (1H, br s, NH), 7.72 (1H, br s, NH), 7.71 (1H, br s, NH), 7.67 (1H, br s, NH), 7.57 (1H, br s, NH), 7.21 (1H, br s, NH), 6.73 (1H, br s, NH), 1.61 (6H, dd, *J*=129.0, 4.0, *CH_3 x2), 1.57 (6H, s, CH_3 x2), 1.52 (6H, s, CH_3 x2), 1.51 (6H, s, CH_3 x2), 1.49 (6H, s, CH_3 x2), 1.48 (3H, dd, *J*=129.0, 4.0, *CH_3 x2 (50%)), 1.48 (15H, s, CH_3 x2; CH_3 x2 (50%)), 1.44 (4.5H, s, CH_3 x2 (75%)),

1.44 (1.5H, dd, *J*=129.0, 4.0, *CH_3 x2 (25%)). ^{13}C-NMR (126 MHz, $CDCl_3$) δ_C 176.5 (CO), 176.2 (CO), 176.1 (CO), 175.7 (CO), 175.7 (CO), 175.5 (CO), 174.4 (CO), 173.6 (CO), 173.3 (CO), 63.9 (αC), 56.9 (αC), 56.8 (αC), 56.8 (αC), 56.7 (αC), 56.7 (αC), 56.6 (αC), 30.3 (CH_3), 29.7 (CH_3), 25.3 ($^{13}CH_3$ x2 (100%)), 24.3 – 25.5 (all other *CH_3 and CH_3). The triplet signals arising from the carbons alpha to the ^{13}C-labels could not be located. IR (neat) ν_{max}/cm^{-1} = 3279, 2985, 2936, 2114, 1738, 1650, 1537, 1454. HRMS (ES$^+$, MeOH) Calc. for $C_{34}{}^{13}C_2H_{64}N_{11}O_{10}$ ([M+H]$^+$) 812.4899, found 812.4902; Calc. for $C_{32}{}^{13}C_4H_{64}N_{11}O_{10}$ ([M+H]$^+$) 814.4966, found 814.4963; Calc. for $C_{30}{}^{13}C_6H_{64}N_{11}O_{10}$ ([M+H]$^+$) 816.5033, found 816.5030. Mp >300 °C.

N_3-Aib$_2$Aib$^{25\%}$Aib$_2$Aib$^{50\%}$**Aib$_2$Aib**Aib$_2$-O^{t}Bu 6a**

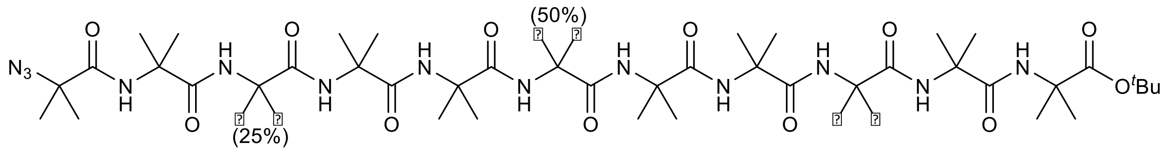

N_3-Aib$_2$Aib$^{25\%}$Aib$_2$Aib$^{50\%}$**Aib$_2$Aib**-OH** (100 mg, 0.123 mmol) was dissolved in CH_2Cl_2 (5 mL) and the resulting solution cooled to 0 °C. *N*-(3-Dimethylaminopropyl)-*N*′-ethylcarbodiimide hydrochloride (35 mg, 0.184 mmol) and *N,N*-diisopropylethylamine (32 μL, 0.184 mmol) were added, the reaction mixture was allowed to warm to room temperature and stirred for 3 h. The solution was then concentrated *in vacuo*, EtOAc (15 mL) was added and the organic phase washed with $KHSO_4$ (10%, 3 x 3 mL), brine (3 mL), dried ($MgSO_4$), filtered and concentrated *in vacuo.* The crude azlactone was then place under high vacuum (<0.1 mbar) before being dissolved in MeCN (3 mL) and CH_2Cl_2 (1 mL) with **H-Aib$_2$-O^{t}Bu** (41 mg, 0.123 mmol) and the resulting mixture was heated to reflux for 5 d. The solvent was removed *in vacuo*, CH_2Cl_2 (20 mL) was added and the organic phase washed with HCl (1 M, 2 x 5 mL), brine (5 mL), dried ($MgSO_4$), filtered and concentrated. The crude residue was purified by column chromatography (2-10% MeOH in CH_2Cl_2) to give **N_3-Aib$_2$Aib$^{25\%}$**Aib$_2$Aib$^{50\%}$**Aib$_2$Aib**Aib$_2$-O^{t}Bu 6a** (56 mg, 44%) as a white solid. ^{1}H-NMR (400 MHz, $CDCl_3$) δ_H 7.71 (1H, br s, NH), 7.66 (3H, br m, NH x3), 7.64 (1H, br s, NH), 7.56 (1H, br s, NH), 7.40 (2H, br m, NH x2), 7.04 (1H, br s, NH), 6.26 (1H, br s, NH), 1.56 (6H, s, CH_3 x2), 1.54 (6H, s, CH_3 x2), 1.50 (12H, m, CH_3 x4), 1.50 (6H, dd, *J*=129.0, 4.5, *CH_3 x2), 1.49 (3H, dd, *J*=129.0, 4.5, *CH_3 x2 (50%)), 1.49 (6H, s, CH_3 x2), 1.49 (9H, m, CH_3 x2 and CH_3 x2 (50%)), 1.48 (12H, s, CH_3 x4), 1.44 (13.5H, m, $(CH_3)_3$ and CH_3 x2 (75%)), 1.44 (1.5H, dd, *J*=129.0, 4.0, *CH_3 x2 (25%)). ^{13}C-NMR (101 MHz, $CDCl_3$) δ_C 175.8 (CO), 175.7 (CO), 175.7 (CO), 175.5 (CO), 175.4 (CO), 174.3 (CO), 174.3 (CO), 174.2 (CO), 174.2 (CO), 173.3 (CO), 173.3 (CO), 79.6 (CMe_3), 63.9 (αC), 56.9 (αC), 56.8 (αC), 56.6 (αC), 56.6 (αC), 56.6 (αC x2), 55.9 (αC), 27.9 (C(*C*)H_3)$_3$), 23.6-26.4 (all CH_3 and *CH_3). The triplet signals arising from the carbons alpha to the ^{13}C-labels could not be located. IR (neat) ν_{max}/cm^{-1} = 3293, 2982, 2935, 2112, 1736, 1649 1531, 1455. MS (ES$^+$, MeOH) 1038.8 (48%, [($^{13}C_2$)M+H]$^+$), 1040.8 (100%, [($^{13}C_4$)M+H]$^+$), 1042.9 (45%, [($^{13}C_6$)M+H]$^+$). Mp >300 °C.

N_3-Aib$_2$Aib$^{25\%}$Aib$_2$Aib$^{50\%}$**Aib$_2$Aib**Aib$_3$O^{t}Bu 6b**

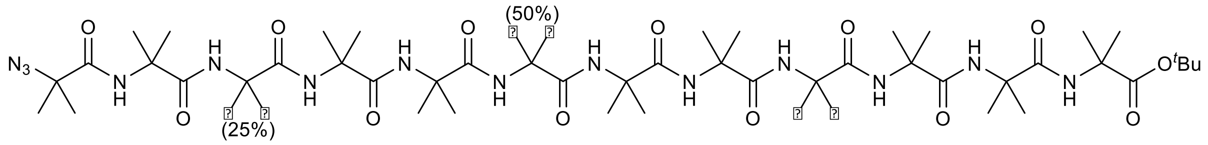

N_3-$Aib_2Aib^{25\%}$ $Aib_2Aib^{50\%}$**Aib_2Aib**-OH** (100 mg, 0.123 mmol) was dissolved in CH_2Cl_2 (5 mL) and the resulting solution cooled to 0 °C. *N*-(3-Dimethylaminopropyl)-*N*′-ethylcarbodiimide (0.026 mL, 0.147 mmol) was added, the reaction mixture was allowed to warm to room temperature and stirred for 3 h. The solution was then concentrated *in vacuo*, EtOAc (15 mL) was added and the organic phase washed with $KHSO_4$ (10%, 3 x 3 mL), brine (3 mL), dried ($MgSO_4$), filtered and concentrated *in vacuo.* The crude azlactone was then place under high vacuum (<0.1 mbar) before being dissolved in MeCN (3 mL) with **H-Aib_3-O*t*Bu** (41 mg, 0.123 mmol) and the resulting mixture was heated to reflux for 5 d. The solvent was removed *in vacuo*, CH_2Cl_2 (20 mL) was added and the organic phase washed with HCl (1 M, 2 x 5 mL), brine (7 mL), dried ($MgSO_4$), filtered and concentrated. The crude residue was purified by column chromatography (1-5% MeOH in CH_2Cl_2) to give **N_3-$Aib_2Aib^{25\%}$**$Aib_2Aib^{50\%}$**Aib_2Aib**Aib_3-O*t*Bu 6b** (47 mg, 34%) as a white solid. ^{1}H-NMR (400 MHz, $CDCl_3$) δ_H 7.73 (1H, br s, NH), 7.71 (1H, br s, NH), 7.67 (2H, br s, NH x2), 7.66 (1H, br s, NH), 7.63 (1H, br s, NH), 7.56 (1H, br s, NH), 7.40 (2H, br s, NH), 7.01 (1H, br s, NH), 6.26 (1H, br s, NH), 1.71 (6H, s, CH_3 x2), 1.57 (6H, s, CH_3 x2), 1.54 (6H, s, CH_3 x2), 1.51 (6H, s, CH_3 x2), 1.50 (6H, dd, *J*=129.0, 4.5, *CH_3 x2), 1.50 (6H, s, CH_3 x2), 1.50 (12H, s, CH_3 x4), 1.49 (3H, dd, *J*=129.0, 4.5, *CH_3 x2 (50%)), 1.49 (9H, m, CH_3 x2 and CH_3 x2 (50%)), 1.48 (12H, s, CH_3 x4), 1.44 (1.5H, dd, *J*=129.0, 4.0, *CH_3 x2 (25%)), 1.44 (13.5H, m, $(CH_3)_3$ and CH_3 x2 (75%)). ^{13}C-NMR (101 MHz, $CDCl_3$) δ_C 175.9 (CO), 175.9 (CO), 175.7 (CO), 175.7 (CO), 175.6 (CO), 175.4 (CO), 174.3 (CO), 174.3 (CO), 174.2 (CO), 174.1 (CO), 173.3 (CO), 173.3 (CO), 79.7 (CMe_3), 63.9 (αC), 56.9 (αC), 56.8 (αC), 56.8 (αC), 56.6 (αC x2), 56.6 (αC x3), 56.0 (αC), 27.9 ($C(CH_3)_3$), 23.3-26.7 (all CH_3 and *CH_3). The triplet signals arising from the carbons alpha to the ^{13}C-labels could not be located. IR (neat) ν_{max}/cm^{-1} = 3281, 2982, 2936, 2113, 1648, 1535, 1455. MS (ES$^+$, MeOH) 1126.2 (60%, [($^{13}C_2$)M+H]$^+$), 1124.2 (100%, [($^{13}C_4$)M+H]$^+$), 1128.1 (48%, [($^{13}C_6$)M+H]$^+$). Mp >300 °C.

N_3-$Aib_2Aib^{25\%}$ $Aib_2Aib^{50\%}$**Aib_2Aib**Aib_4O^tBu 6c**

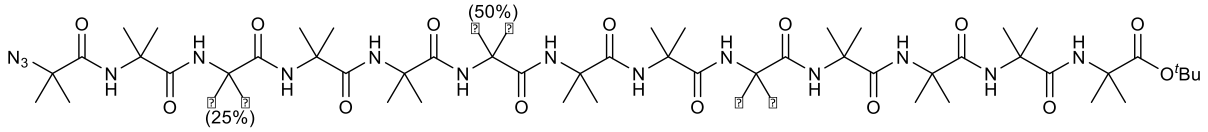

N_3-$Aib_2Aib^{25\%}$ $Aib_2Aib^{50\%}$**Aib_2Aib**-OH** (100 mg, 0.123 mmol) was dissolved in CH_2Cl_2 (5 mL) and the resulting solution cooled to 0 °C. *N*-(3-Dimethylaminopropyl)-*N*′-ethylcarbodiimide (0.026 mL, 0.147 mmol) was added, the reaction mixture was allowed to warm to room temperature and stirred for 3 h. The solution was then concentrated *in vacuo*, EtOAc (15 mL) was added and the organic phase washed with $KHSO_4$ (10%, 3 x 3 mL), brine (3 mL), dried ($MgSO_4$), filtered and concentrated *in vacuo.* The crude azlactone was then place under high vacuum (<0.1 mbar) before being dissolved in MeCN (3 mL) with **H-Aib_4-O*t*Bu** (51 mg, 0.123 mmol) and the resulting mixture was heated to reflux for 5 d. The solvent was removed *in vacuo*, CH_2Cl_2 (20 mL) was added and the organic phase washed with HCl (1 M, 2 x 5 mL), brine (7 mL), dried ($MgSO_4$), filtered and

concentrated. The crude residue was purified by column chromatography (3-10% MeOH in CH_2Cl_2) to give **N_3-Aib$_2$Aib$^{25\%}$**Aib$_2$Aib$^{50\%}$**Aib$_2$Aib**Aib$_4$-O*t*Bu 6c** (61 mg, 41%) as a white solid. ^{1}H-NMR (500 MHz, $CDCl_3$) δ_H 7.74 (2H, br m, NH x2), 7.73 (1H, br s, NH), 7.68 (2H, br m, NH x2), 7.66 (1H, br s, NH), 7.64 (1H, br s, NH), 7.56 (1H, br s, NH), 7.41 (2H, br m, NH x2), 7.03 (1H, br s, NH), 6.26 (1H, br s, NH), 1.57 (6H, s, CH_3 x2), 1.54 (6H, s, CH_3 x2), 1.51 (12H, s, CH_3 x4), 1.51 (6H, s, CH_3 x2), 1.50 (12H, s, CH_3 x4), 1.50 (9H, m, CH_3 x2 and CH_3 x2 (50%)), 1.49 (9H, m, *CH_3 and *CH_3(50%)), 1.48 (12H, s, CH_3 x2), 1.44 (13.5H, m, $C(CH_3)_3$ and CH_3 x2 (75%)), 1.44 (1.5H, m, *CH_3 x2 (25%)). ^{13}C-NMR (126 MHz, $CDCl_3$) δ_C 176.0 (CO), 176.0 (CO), 175.9 (CO), 175.8 (CO), 175.7 (CO), 175.6 (CO), 175.4 (CO), 174.3 (CO), 174.3 (CO), 174.3 (CO), 174.2 (CO), 173.3 (CO), 173.3 (CO), 79.6 (CMe_3), 63.9 ($^{\alpha}$C), 56.9 ($^{\alpha}$C), 56.8 ($^{\alpha}$C), 56.8 ($^{\alpha}$C), 56.6 ($^{\alpha}$C), 56.6 ($^{\alpha}$C), 56.6 ($^{\alpha}$C x3), 56.0 ($^{\alpha}$C), 27.9, $C(CH_3)_3$), 23.2-26.7 (all CH_3 and *CH_3). The triplet signals arising from the carbons alpha to the ^{13}C-labels could not be located. IR (neat) ν_{max}/cm^{-1} = 3281, 2982, 2936, 2113, 1648, 1535, 1455. MS (ES$^+$, MeOH) 1209.3 (60%, $[(^{13}C_2)M+H]^+$), 1211.2 (100%, $[(^{13}C_4)M+H]^+$), 1213.4 (45%, $[(^{13}C_6)M+H]^+$). Mp >300 °C.

N_3-Aib$_2$Aib$^{25\%}$Aib$_2$Aib$^{50\%}$**Aib$_2$Aib**Aib$_2$-OH**

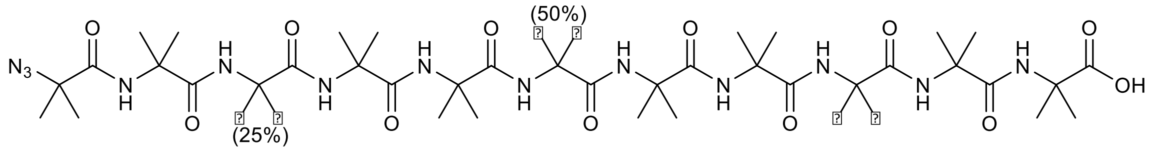

N_3-Aib$_2$Aib$^{25\%}$Aib$_2$Aib$^{50\%}$**Aib$_2$Aib**Aib$_2$-O^{t}Bu 6a** (55 mg, 0.053 mmol) was dissolved in CH_2Cl_2 (1 mL) and trifluoroacetic acid (100 μL) was added dropwise. After 2 h, the solvents were removed *in vacuo* and the crude residue purified by column chromatography (2-10% MeOH in CH_2Cl_2) to give **N_3-Aib$_2$Aib$^{25\%}$**Aib$_2$Aib$^{50\%}$**Aib$_2$Aib**Aib$_2$-OH** (46 mg, 88%) as a white solid. ^{1}H-NMR (400 MHz, CD_3OD) δ_H 7.99 (1H, br s, NH), 7.93 (1H, br s, NH), 7.91 (5H, br m, NH x5), 7.82 (1H, br s, NH), 7.80 (1H, br s, NH), 7.67 (1H, br s, NH), 1.54 (6H, s, CH_3 x2), 1.49 (9H, m, *CH_3 x2 and CH_3 x2 (50%)), 1.49 (39H, m, CH_3 x12 and CH_3 x2 (50%)), 1.46 (6H, s, CH_3 x2), 1.41 (4.5H, s, CH_3 x2 (75%), 1.41 (1.5H, dd, *J*=129.0, 4.0, *CH_3 x2 (25%)). ^{13}C-NMR (126 MHz, 90:10 $CDCl_3$:CD_3OD) δ_C 176.2 (CO x4), 176.1 (CO), 175.9 (CO), 175.1 (CO), 174.4 (CO), 174.3 (CO), 173.3 (CO), 173.2 (CO), 63.7 ($^{\alpha}$C), 57.0 ($^{\alpha}$C), 56.8 ($^{\alpha}$C), 56.7 ($^{\alpha}$C), 56.6 ($^{\alpha}$C), 56.5 ($^{\alpha}$C x2), 56.5 ($^{\alpha}$C), 56.4 ($^{\alpha}$C), 29.6 (CH_3 x2), 22.0 – 27.7 (all other CH_3 and *CH_3). The triplet signals arising from the carbons alpha to the ^{13}C-labels could not be located. IR (neat)ν_{max}/cm^{-1} = 3383, 2984, 2935, 2113, 1651, 1533, 1456. MS (ES$^+$, MeOH) 1004.9 (45%, $[(^{13}C_2)M+H]^+$), 1007.0 (100%, $[(^{13}C_4)M+H]^+$), 1008.3 (40%, $[(^{13}C_6)M+H]^+$), Mp >300 °C.

N_3-Aib$_2$Aib$^{25\%}$Aib$_2$Aib$^{50\%}$**Aib$_2$Aib**Aib$_3$-OH**

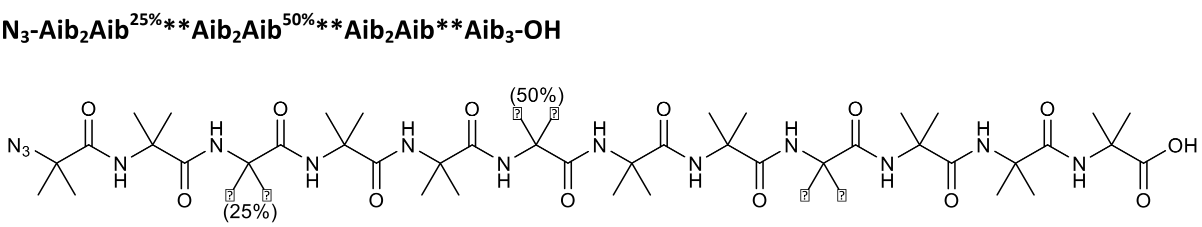

N_3-Aib$_2$Aib$^{25\%}$Aib$_2$Aib$^{50\%}$**Aib$_2$Aib**Aib$_3$-O^{t}Bu 6b** (42 mg, 0.037 mmol) was dissolved in CH_2Cl_2 (1 mL) and trifluoroacetic acid (100 μL) was added dropwise. After 2 h, the solvents were removed *in vacuo* and the crude residue purified by column chromatography (2-10% MeOH in CH_2Cl_2) to give **N_3-Aib$_2$Aib$^{25\%}$**Aib$_2$Aib$^{50\%}$**Aib$_2$Aib**Aib$_3$-OH** (39 mg, 99%) as a white solid. ^{1}H-NMR (500 MHz, $CDCl_3$) δ_H 7.90 (1H, br s, NH), 7.76 (4H, br m, NH x4), 7.72 (2H, br m, NH x2), 7.67 (1H, br s, NH), 7.58 (2H, br

m, NH x2), 6.80 (1H, br s, NH), 1.62 (6H, s, CH_3 x2), 1.58 (28 H, s), 1.52 (9H, m, CH_3 x2 and CH_3 x2 (50%)), 1.52 (9H, m, *CH_3 x2 and *CH_3 x2 (50%)), 1.49 (30H, m, CH_3 x 10), 1.45 (4.5H, s, CH_3 x2 (75%)), 1.45 (1.5H, *CH_3 x2 (25%). ^{13}C-NMR (126 MHz, $CDCl_3$) δ_C 176.2 (CO x3), 176.2 (CO), 176.1 (CO x2), 175.9 (CO), 175.9 (CO), 175.2 (CO), 175.0 (CO), 173.4 (CO x2), 63.8 (αC), 56.9 (αC), 56.8 (αC), 56.7 (αC), 56.6 (αC), 56.6 (αC), 56.5 (αC), 56.5 (αC x2), 29.7 (CH_3 x2), 21.7 – 27.8 (all other CH_3 and *CH_3). The triplet signals arising from the carbons alpha to the ^{13}C-labels could not be located. IR (neat) ν_{max}/cm^{-1} = 3286, 2985, 2935, 2115, 1652, 1534, 1469, 1417. MS (ES$^+$, CH_2Cl_2) 1090.0 (35%, [($^{13}C_2$)M+H]$^+$), 1092.0 (100%, [($^{13}C_4$)M+H]$^+$), 1094.1 (20%, [($^{13}C_6$)M+H]$^+$). Mp >300 °C.

N_3-Aib$_2$Aib$^{25\%}$Aib$_2$Aib$^{50\%}$**Aib$_2$Aib**Aib$_4$-OH**

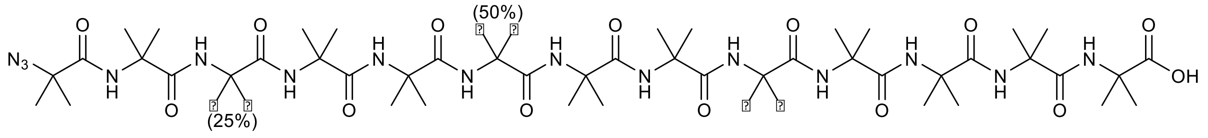

N_3-Aib$_2$Aib$^{25\%}$Aib$_2$Aib$^{50\%}$**Aib$_2$Aib**Aib$_4$-O^{t}Bu 6c** (34 mg, 0.028 mmol) was dissolved in CH_2Cl_2 (1 mL) and trifluoroacetic acid (100 μL) was added dropwise. After 2 h, the solvents were removed *in vacuo* and the crude residue purified by column chromatography (2-10% MeOH in CH_2Cl_2) to give **N_3-Aib$_2$Aib$^{25\%}$**Aib$_2$Aib$^{50\%}$**Aib$_2$Aib**Aib$_4$-OH** (31 mg, 96%) as a white solid. ^{1}H-NMR (400 MHz, $CDCl_3$) δ_H 7.86 (1H, br s, NH), 7.76 (8H, br m, NH x8), 7.60 (1H, br s, NH), 7.42 (1H, br s, NH), 7.02 (1H, br s, NH), 1.56 (6H, s, CH_3 x2), 1.48 (6H, dd, *J*=129.0, 4.0, *CH_3 x2), 1.48 (3H, m, *CH_3 x2 (50%)), 1.45 – 1.53 (57H, m, CH_3 x18 and CH_3 x2 (50%), 1.43 (4.5H, s, CH_3 x2 (75%)), 1.43 (1.5H, m, *CH_3 x2 (25%)).^{13}C-NMR (101 MHz, 95:5 $CDCl_3$:CD_3OD) δ_C 176.3 (CO), 176.3 (CO x2), 176.2 (CO x2), 176.0 (CO), 175.9 (CO x2), 175.0 (CO), 174.3 (CO), 174.2 (CO), 173.3 (CO), 173.3 (CO), 63.8 (αC), 56.9 (αC), 56.8 (αC), 56.8 (αC), 56.6 (αC x2), 56.5 (αC x2), 56.5 (αC), 29.7 (CH_3 x2), 21.2 – 28.1 (all other CH_3 and *CH_3). The triplet signals arising from the carbons alpha to the ^{13}C-labels could not be located. IR (neat) ν_{max}/cm^{-1} = 3278, 2985, 2117, 1653, 1540, 1456. MS (ES$^+$, MeOH) 1175.4 (45%, [($^{13}C_2$)M+H]$^+$), 1177.4 (100%, [($^{13}C_4$)M+H]$^+$), 1179.4 (20%, [($^{13}C_6$)M+H]$^+$). Mp >300 °C.

N_3-Aib$_2$Aib$^{25\%}$Aib$_2$Aib$^{50\%}$**Aib$_2$Aib**Aib$_2$Ala-NH^{t}Bu 7a**

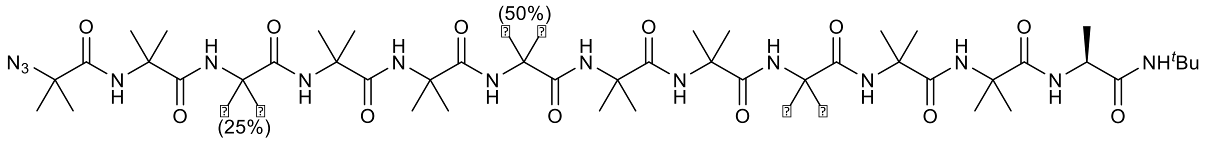

N_3-Aib$_2$Aib$^{25\%}$Aib$_2$Aib$^{50\%}$**Aib$_2$Aib**Aib$_2$-OH** (46 mg, 0.047 mmol) and 1-Hydroxybenzotriazole hydrate (11 mg, 0.061 mmol) were dissolved in CH_2Cl_2 (4 mL) and the suspension cooled to 0 °C. *N*-(3-Dimethylaminopropyl)-*N*′-ethylcarbodiimide hydrochloride (10 mg, 0.051 mmol) and triethylamine (10 μL, 0.07 mmol) were added and the reaction was allowed to warm to room temperature and stirred until it was homogenous. H-Ala-NH^{t}Bu (13 mg, 0.09 mmol) and triethylamine (10 μL, 0.07 mmol) were added and the reaction mixture stirred for 72 h. The solvent was removed *in vacuo* and CH_2Cl_2 (15 mL) was added. The organic phase was washed with $KHSO_4$ (5%, 2 x 5 mL), $NaHCO_3$ (2 x 5 mL), brine (5 mL), dried (Na_2SO_4), filtered and concentrated. The crude residue was purified by column chromatography (1-5% MeOH in CH_2Cl_2) to give **N_3-**

Aib$_2$Aib$^{25\%}$Aib$_2$Aib$^{50\%}$**Aib$_2$Aib**Aib$_2$Ala-NH^{t}Bu 7a** (26 mg, 50%) as a white solid. ^{1}H-NMR (400 MHz, $CDCl_3$) δ_H 7.74 (5H, br m, NH x5), 7.69 (1H, br s, NH), 7.67 (1H, br s, NH), 7.57 (1H, br s, NH), 7.56 (1H, br s, NH), 7.12 (1H, br s, NH), 6.91 (1H, br s, NH), 6.41 (1H, br s, NH), 4.32 (1H, dq, *J*=7.5, 7.5, αCH), 1.60 (3H, s, CH_3), 1.58 (3H, s, CH_3), 1.56 (3H, s, CH_3), 1.53 (6H, s, CH_3 x2), 1.52 (3H, s, CH_3), 1.50 (3H, dd, *J*=129.0, 4.0, *CH_3), 1.49 (3H, dd, *J*=129.0, 4.0, *CH_3), 1.46 – 1.51 (30H, m, CH_3 x10), 1.46 (3H, dd, *J*=129.0, 4.5, *CH_3 x2 (50%)), 1.46 (3H, s, CH_3 x2 (50%)), 1.40 (1.5H, m, *CH_3 x2 (25%), 1.40 (13.5H, m, $C(CH_3)_3$ and CH_3 x2 (75%)). ^{13}C-NMR (101 MHz, $CDCl_3$) δ_C 176.2 (CO), 176.1 (CO), 176.0 (CO x2), 175.8 (CO x2), 175.5 (CO), 175.2 (CO), 174.3 (CO), 173.5 (CO), 173.3 (CO), 172.8 (CO), 63.9 (αC), 56.9 (αC), 56.9 (αC), 56.8 (αC), 56.7 (αC), 56.6 (αC), 56.6 (αC), 56.5 (αC), 50.9 (CMe_3), 50.2 (αCH), 28.7 ($C(CH_3)_3$), 26.3 – 27.4 (*CH_3 (25,50,100%) and CH_3 x6), 24.9 (CH_3), 24.4 (CH_3), 24.2 (CH_3), 22.3 – 23.4 (*CH_3 (25,50,100%) and CH_3 x6), 23.4 (CH_3), 17.3 (*C*H_3-CH). The triplet signals arising from the carbons alpha to the ^{13}C-labels could not be located. IR (neat)ν_{max}/cm^{-1} = 3394, 2986, 2469, 2113, 1645, 1531, 1472, 1418. MS (ES$^+$, MeOH) 1131.1 (55%, $[(^{13}C_2)M+H]^+$), 1133.1 (100%, $[(^{13}C_4)M+H]^+$), 1135.2 (25%, $[(^{13}C_6)M+H]^+$). Mp >300 °C. $[\alpha]_D^{20}$ = 117.6 (*c* 0.5, CH_2Cl_2).

N$_3$-Aib$_2$Aib$^{25\%}$Aib$_2$Aib$^{50\%}$**Aib$_2$Aib**Aib$_3$Ala-NH^{t}Bu 7b**

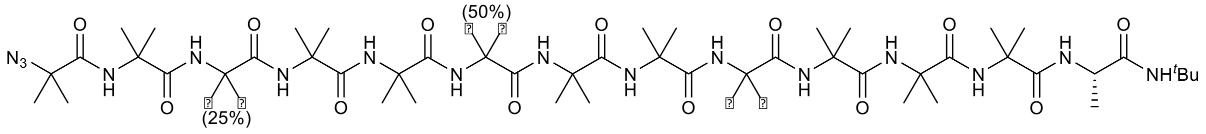

N$_3$-Aib$_2$Aib$^{25\%}$Aib$_2$Aib$^{50\%}$**Aib$_2$Aib**Aib$_3$-OH** (37 mg, 0.035 mmol) and 1-Hydroxybenzotriazole hydrate (8 mg, 0.045 mmol) were dissolved in CH_2Cl_2 (4 mL) and the suspension cooled to 0 °C. *N*-(3-Dimethylaminopropyl)-*N*′-ethylcarbodiimide hydrochloride (7 mg, 0.051 mmol) and triethylamine (7.5 μL, 0.05 mmol) were added and the reaction was allowed to warm to room temperature and stirred until it was homogenous. H-Ala-NH^{t}Bu (8 mg, 0.055 mmol) and triethylamine (7.5 μL, 0.05 mmol) were added and the reaction mixture stirred for 72 h. The solvent was removed *in vacuo* and CH_2Cl_2 (15 mL) was added. The organic phase was washed with $KHSO_4$ (5%, 2 x 5 mL), $NaHCO_3$ (2 x 5 mL), brine (5 mL), dried (Na_2SO_4), filtered and concentrated. The crude residue was purified by column chromatography (1-5% MeOH in CH_2Cl_2) to give **N$_3$-Aib$_2$Aib$^{25\%}$**Aib$_2$Aib$^{50\%}$**Aib$_2$Aib**Aib$_3$Ala-NH^{t}Bu 7b** (26 mg, 62%) as a white solid. ^{1}H-NMR (400 MHz, $CDCl_3$) δ_H 7.75 (5H, br m, NH x5), 7.73 (1H, br s, NH), 7.70 (1H, br s, NH), 7.69 (1H, br s, NH), 7.58 (1H, br s, NH), 7.57 (1H, br s, NH), 7.18 (1H, br s, NH), 6.91 (1H, br s, NH), 6.50 (1H, br s, NH), 4.31 (1H, dq, *J*=7.5, 7.5, αCH), 1.60 (3H, s, CH_3), 1.57 (3H, s, CH_3), 1.56 (3H, s, CH_3), 1.53 (6H, s, CH_3 x2), 1.52 (3H, s, CH_3), 1.50 (3H, dd, *J*=130.0, 4.5, *CH_3), 1.48 – 1.51 (33H, m, CH_3 x10 and CH_3 x2 (50%)), 1.48 (6H, m, *CH_3 and *CH_3 x2 (50%)), 1.46 (3H, s, CH_3 x2), 1.40 (13.5H, m, $C(CH_3)_3$ and CH_3 x2 (75%)), 1.40 (1.5H, m, *CH_3 x2 (25%)). ^{13}C-NMR (101 MHz, $CDCl_3$) δ_C 176.2 (CO), 176.1 (CO x2), 176.0 (CO x2), 175.9 (CO x2) 175.6 (CO), 175.2 (CO), 174.4 (CO), 173.6 (CO), 173.3 (CO), 172.8 (CO), 63.9 (αC), 56.9 (αC), 56.9 (αC), 56.7 (αC), 56.6 (αC), 56.6 (αC), 56.6 (αC), 56.5 (αC x2), 50.9 (CMe_3), 50.3 (αCH), 28.7 ($C(CH_3)_3$), 26.3 – 27.4 (*CH_3 (25,50,100%) and CH_3 x8), 24.4 (CH_3), 24.2 (CH_3), 22.4 – 23.4 (*CH_3 (25,50,100%) and CH_3 x8), 17.3 (*C*H_3-CH). The triplet signals arising from the carbons alpha to the ^{13}C-labels could not be located. IR (neat)ν_{max}/cm^{-1} = 3294, 2986, 2469, 2113, 1645, 1531, 1418. MS (ES$^+$, CH_2Cl_2) 1216.1 (40%, $[(^{13}C_2)M+Na]^+$), 1218.2 (100%, $[(^{13}C_4)M+Na]^+$), 1220.1 (70%, $[(^{13}C_6)M+Na]^+$). Mp >300 °C. $[\alpha]_D^{20}$ = 176.0 (*c* 0.5, CH_2Cl_2).

N_3-Aib$_2$Aib$^{25\%}$Aib$_2$Aib$^{50\%}$**Aib$_2$Aib**Aib$_4$Ala-NH^{t}Bu 7c**

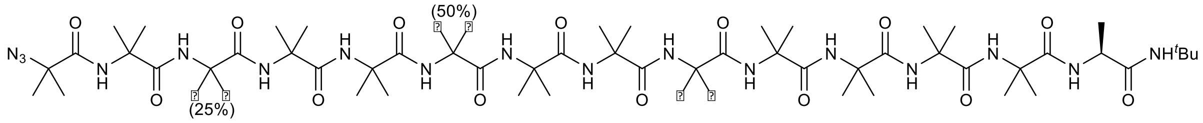

N_3-Aib$_2$Aib$^{25\%}$Aib$_2$Aib$^{50\%}$**Aib$_2$Aib**Aib$_4$-OH** (25 mg, 0.022 mmol) and 1-Hydroxybenzotriazole hydrate (5 mg, 0.028 mmol) were dissolved in CH_2Cl_2 (2 mL) and the suspension cooled to 0 °C. *N*-(3-Dimethylaminopropyl)-*N*'-ethylcarbodiimide hydrochloride (5 mg, 0.024 mmol) and triethylamine (3 μL, 0.022 mmol) were added and the reaction was allowed to warm to room temperature and stirred until it was homogenous. H-Ala-NH^{t}Bu (10 mg, 0.069 mmol) and triethylamine (6 μL, 0.044 mmol) were added and the reaction mixture stirred for 72 h. The solvent was removed *in vacuo* and $CHCl_3$/^{i}PrOH (3:1, 15 mL total) was added. The organic phase was washed with $KHSO_4$ (5%, 2 x 3 mL), $NaHCO_3$ (2 x 3 mL), brine (3 mL), dried (Na_2SO_4), filtered and concentrated. The crude residue was purified by column chromatography (1-5% MeOH in CH_2Cl_2) to give **N_3-Aib$_2$Aib$^{25\%}$**Aib$_2$Aib$^{50\%}$**Aib$_2$Aib**Aib$_4$Ala-NH^{t}Bu 7c** (13 mg, 47%) as a white solid. ^{1}H-NMR (500 MHz, $CDCl_3$) δ_H 7.76 (5H, br m, NH x5), 7.73 (2H, br m, NH x2), 7.69 (1H, br s, NH), 7.66 (1H, br s, NH), 7.57 (1H, br s, NH), 7.56 (1H, br s, NH), 7.13 (1H, br s, NH), 6.91 (1H, br s, NH), 6.43 (1H, br s, NH), 4.32 (1H, dq, *J*=7.0, 7.5, $^\alpha$CH), 1.60 (3H, s, CH_3), 1.58 (3H, s, CH_3), 1.56 (3H, s, CH_3), 1.53 (6H, s, CH_3 x2), 1.52 (3H, dd, *J*=128.0, 4.0, *CH_3), 1.52 (3H, s, CH_3), 1.49 (6H, m, *CH_3 and *CH_3 x2 (50%)), 1.48 – 1.51 (39H, m, CH_3 x12 and CH_3 x2 (50%)), 1.47 (6H, s, CH_3 x2), 1.41 (13.5H, s, $C(CH_3)_3$ and CH_3 x2 (75%)), 1.41 (1.5H, m, *CH_3 x2 (25%)). ^{13}C-NMR (126 MHz, $CDCl_3$) δ_C 176.2 (CO), 176.2 (CO), 176.2 (CO), 176.1 (CO), 176.0 (CO), 176.0 (CO), 175.8 (CO x2), 175.5 (CO), 175.2 (CO), 174.3 (CO), 173.5 (CO), 173.3 (CO), 172.8 (CO), 63.9 ($^\alpha$C), 56.9 ($^\alpha$C), 56.9 ($^\alpha$C), 56.8 ($^\alpha$C), 56.8 ($^\alpha$C), 56.6 ($^\alpha$C), 56.6 ($^\alpha$C), 56.5 ($^\alpha$C x3), 50.9 (CMe_3), 50.2 ($^\alpha$CH), 29.7 (CH_3), 28.8 ($C(CH_3)_3$), 26.1 – 27.6 (*CH_3 (25,50,100%) and CH_3 x6), 25.0 (CH_3), 24.4 (CH_3), 24.2 (CH_3), 22.4 – 23.5 (*CH_3 (25,50,100%) and CH_3 x6),17.3 (CH_3-CH). The triplet signals arising from the carbons alpha to the ^{13}C-labels could not be located. IR (neat) ν_{max}/cm^{-1} = 3283, 1984, 2936, 2113, 1646, 1535, 1421. MS (ES$^+$, CH_2Cl_2) 1301.4 (40%, $[(^{13}C_2)M+Na]^+$), 1302.8 (100%, $[(^{13}C_4)M+Na]^+$), 1304.5 (80%, $[(^{13}C_6)M+Na]^+$). Mp >300 °C. $[\alpha]_D^{20}$ = 27.2(*c* 0.5, CH_2Cl_2).

Example spectra showing ^{13}C-labelled Aib** of 7a

In THF-d_8

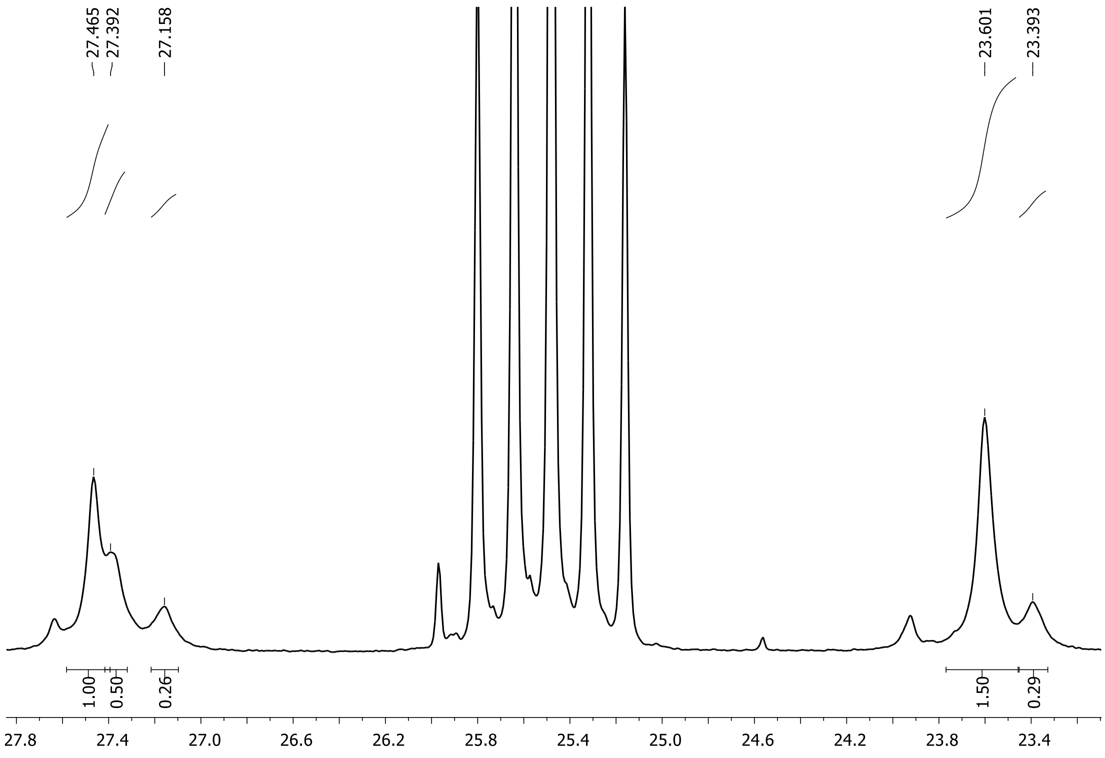

In MeOD/$CDCl_3$ (9:1)

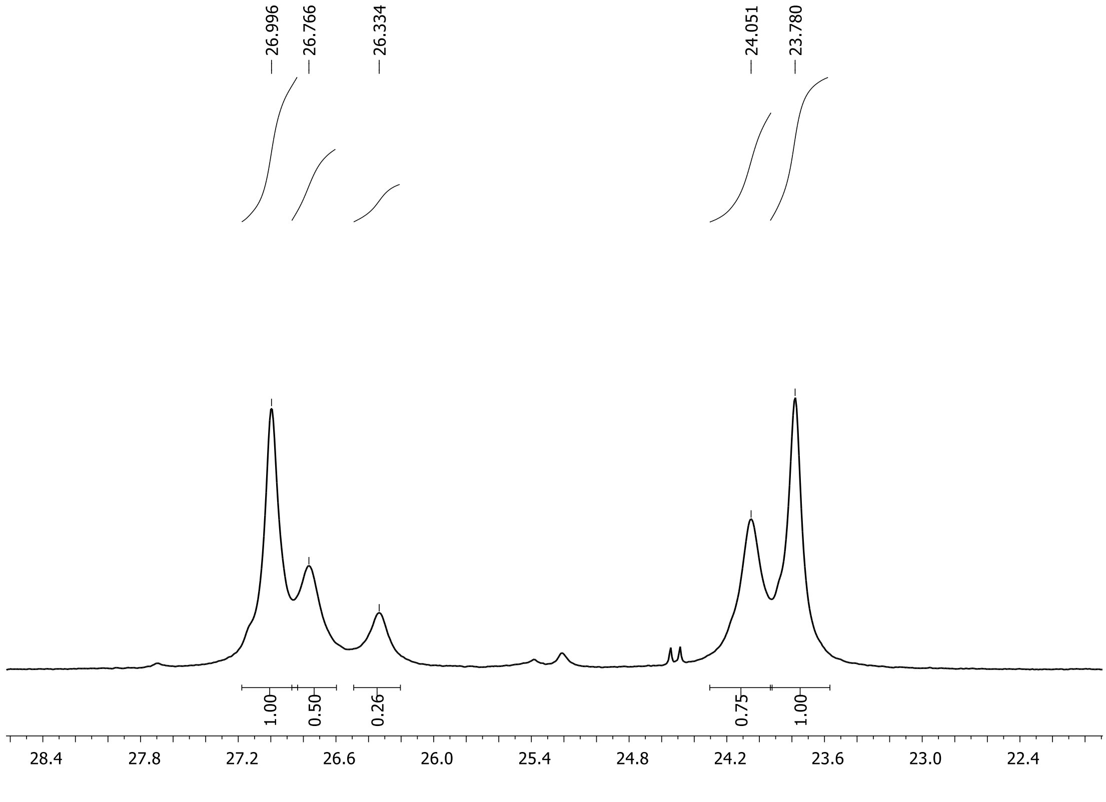

Calculation of decay rate ***f***

$$\Delta\delta_n = \Delta\delta_i.(1-f)^n = \Delta\delta_n.e^{-mn}$$

$$(1-f)^n = e^{-mn}$$

$$nln(1-f) = -mn$$

$$1-f = e^{-m}\ or\ f = 1-e^{-m}$$

Where $\Delta\delta_i$ and m are obtained from the exponential fit of the data using Excel.

Details of NMR experiment used to resolve glycines in compound 3

The PSYCHE[5,6] J-refocusing element was used in a 2D J-resolved pulse sequence to allow the measurement of absorption mode phase-sensitive 2DJ spectra. PSYCHE has the added advantage of attenuating the artefact signals due to strong coupling that can complicate analysis of 2DJ spectra. Adding the F_1 scalar coupling dimension to the PSYCHE experiment gives access both to the high resolution 1D pure shift spectrum, with all homonuclear couplings suppressed, and to shift-resolved absorption mode multiplets from which coupling constants can be determined.

1D PSYCHE

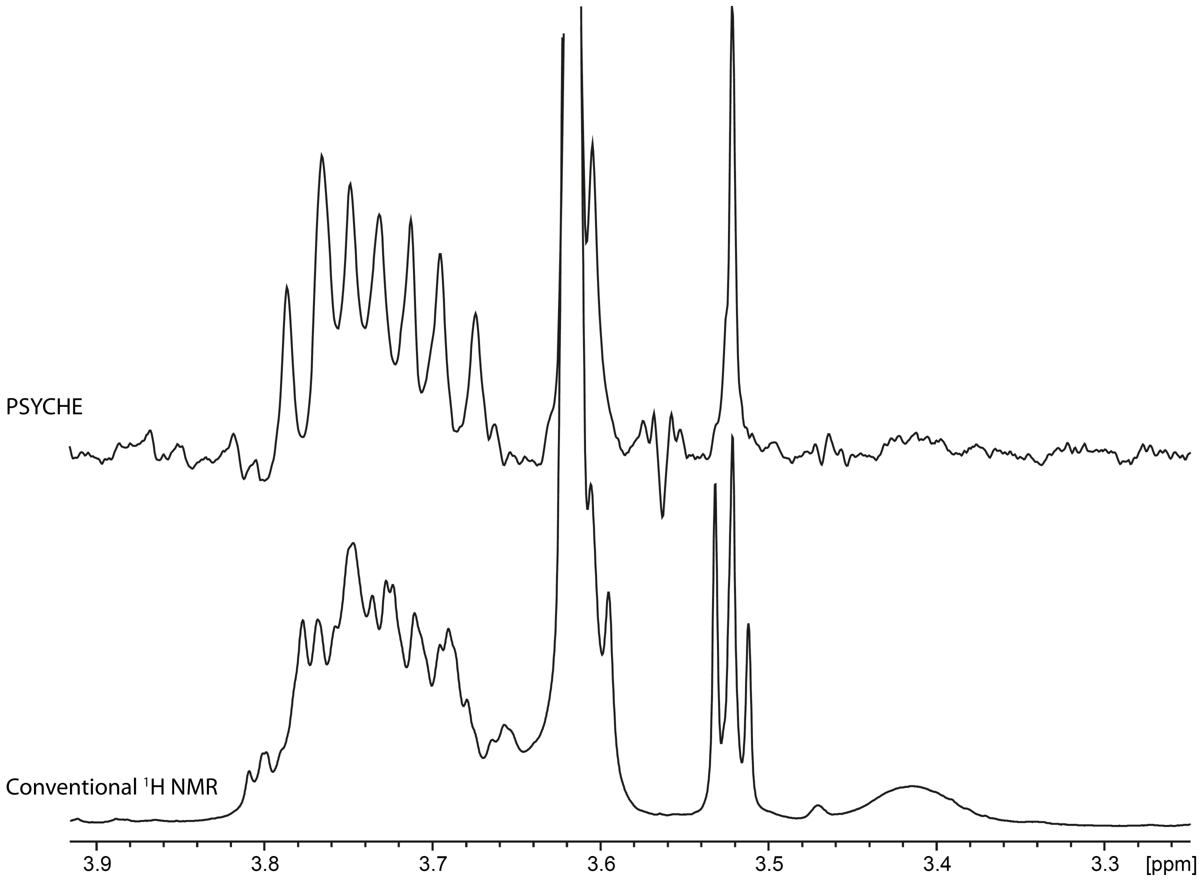

2DJ PSYCHE

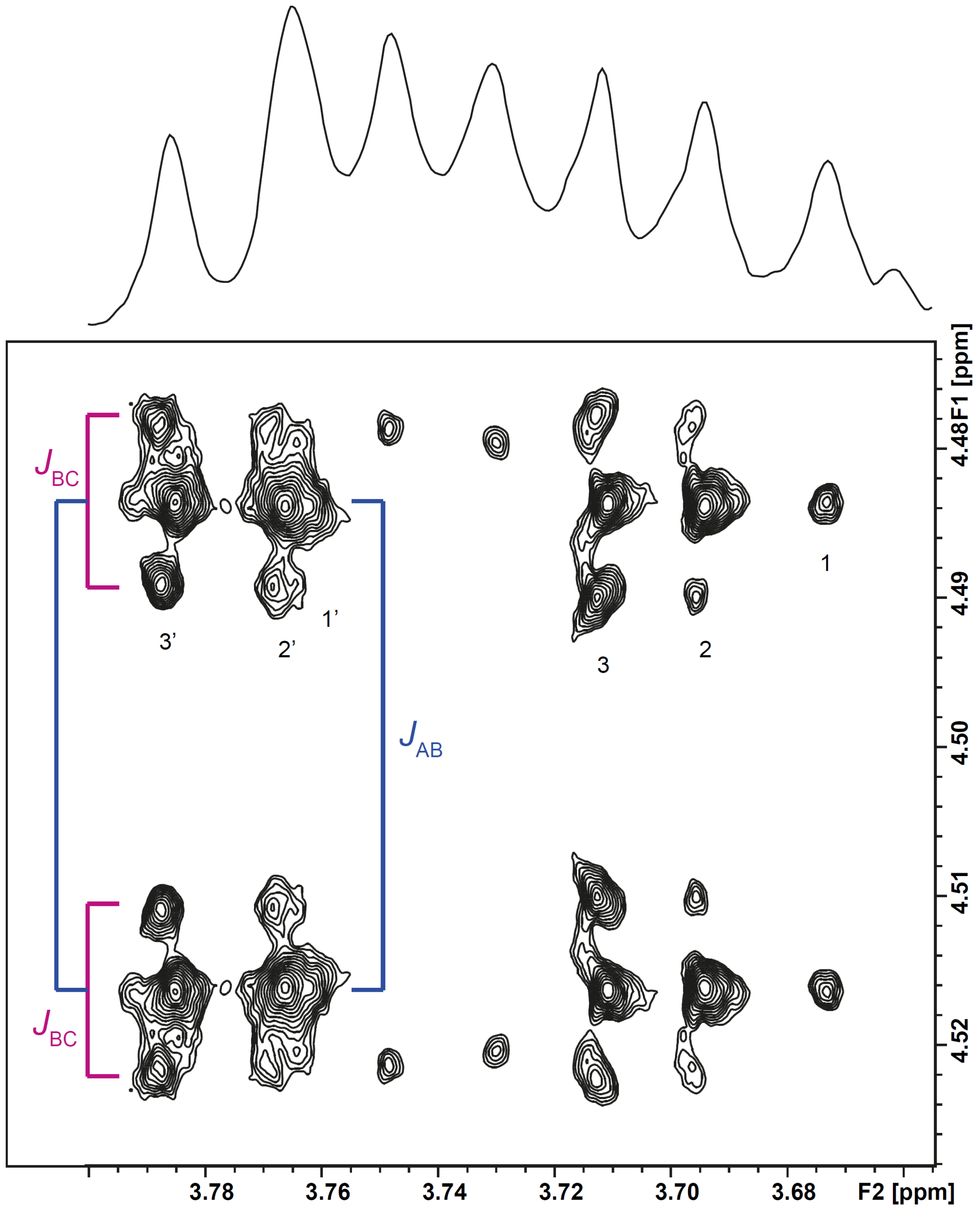

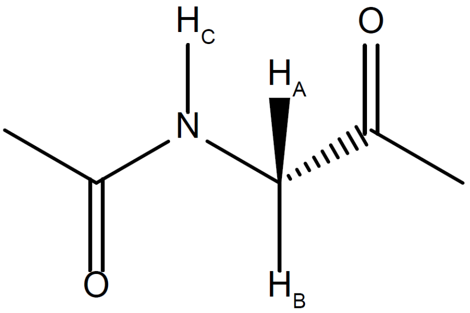

J_{AB} = 16.4 Hz

J_{BC} = 5.2 Hz

J_{AC} = Small

^{1}H-NMR of Aze-Aib$_{11}$AlaNH^{t}Bu 8c

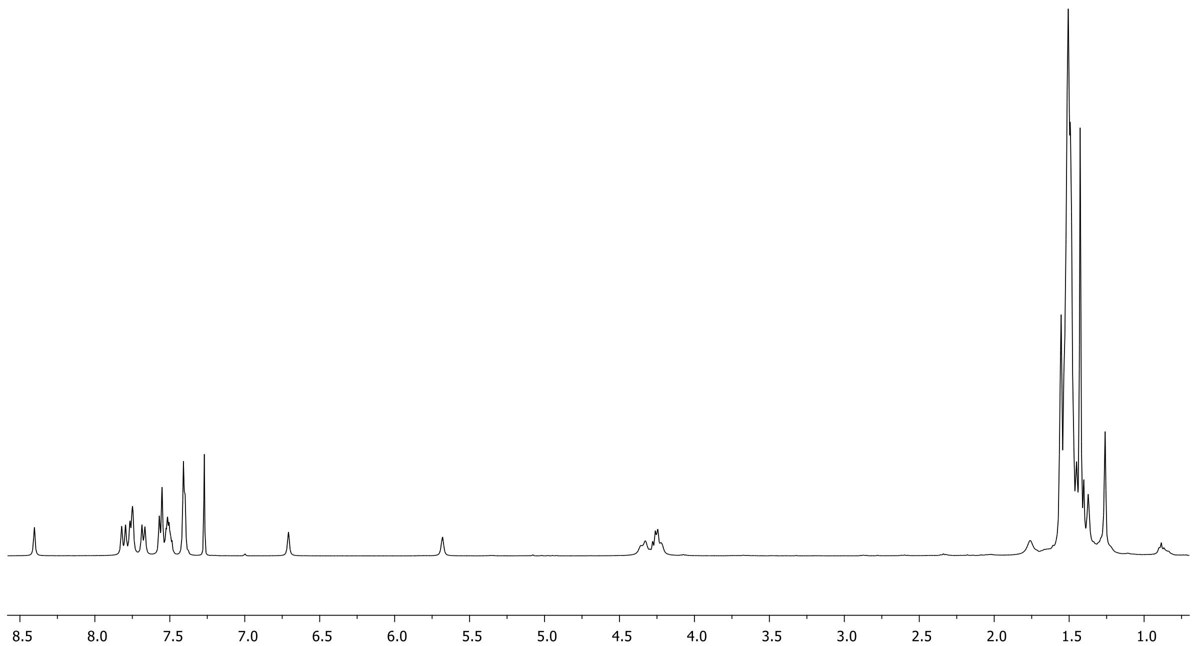

^{13}C-NMR of Aze-Aib$_{11}$AlaNH^{t}Bu 8c

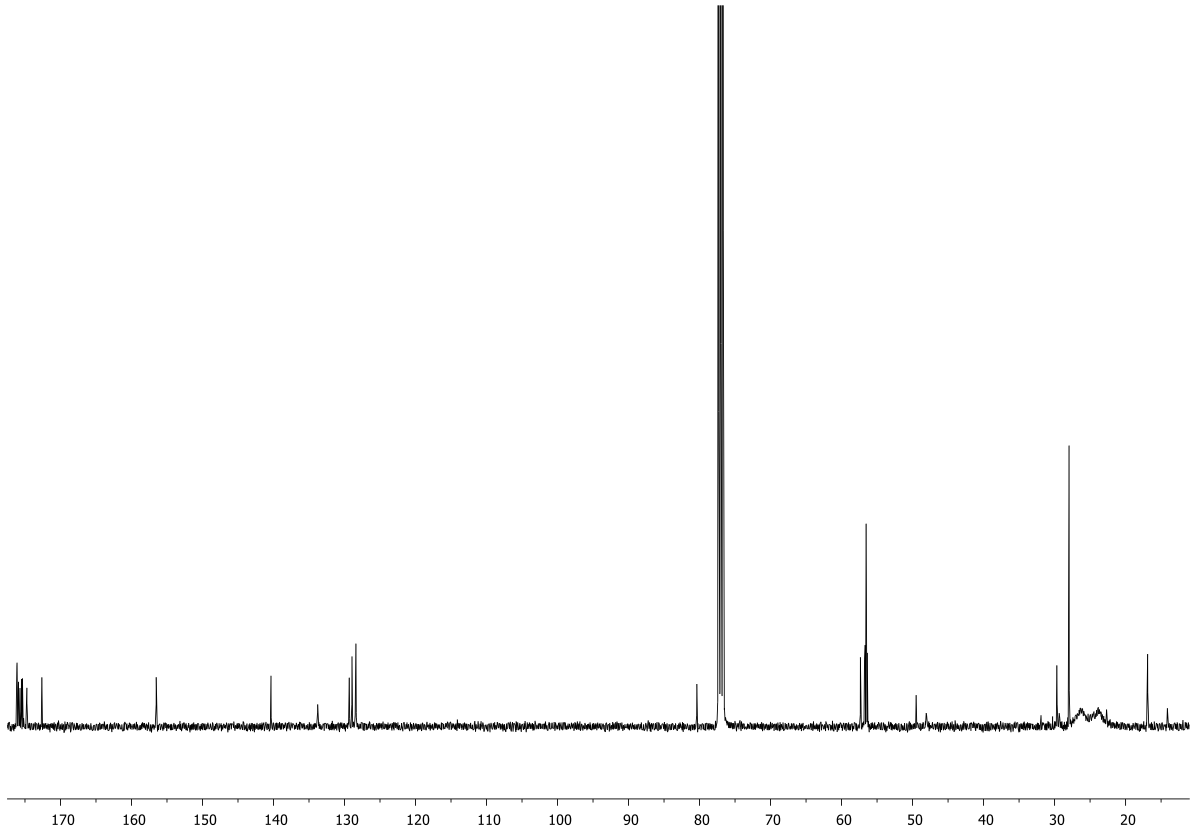

^{1}H-NMR of Aze-Aib$_{12}$AlaNH^{t}Bu 8d

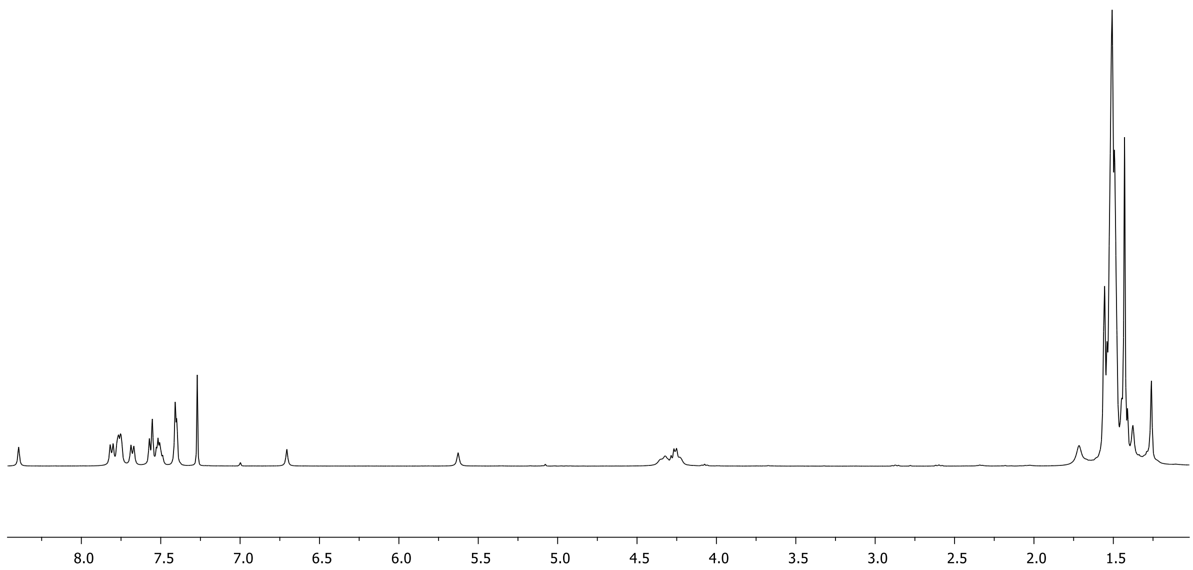

^{13}C-NMR of Aze-Aib$_{12}$AlaNH^{t}Bu 8d

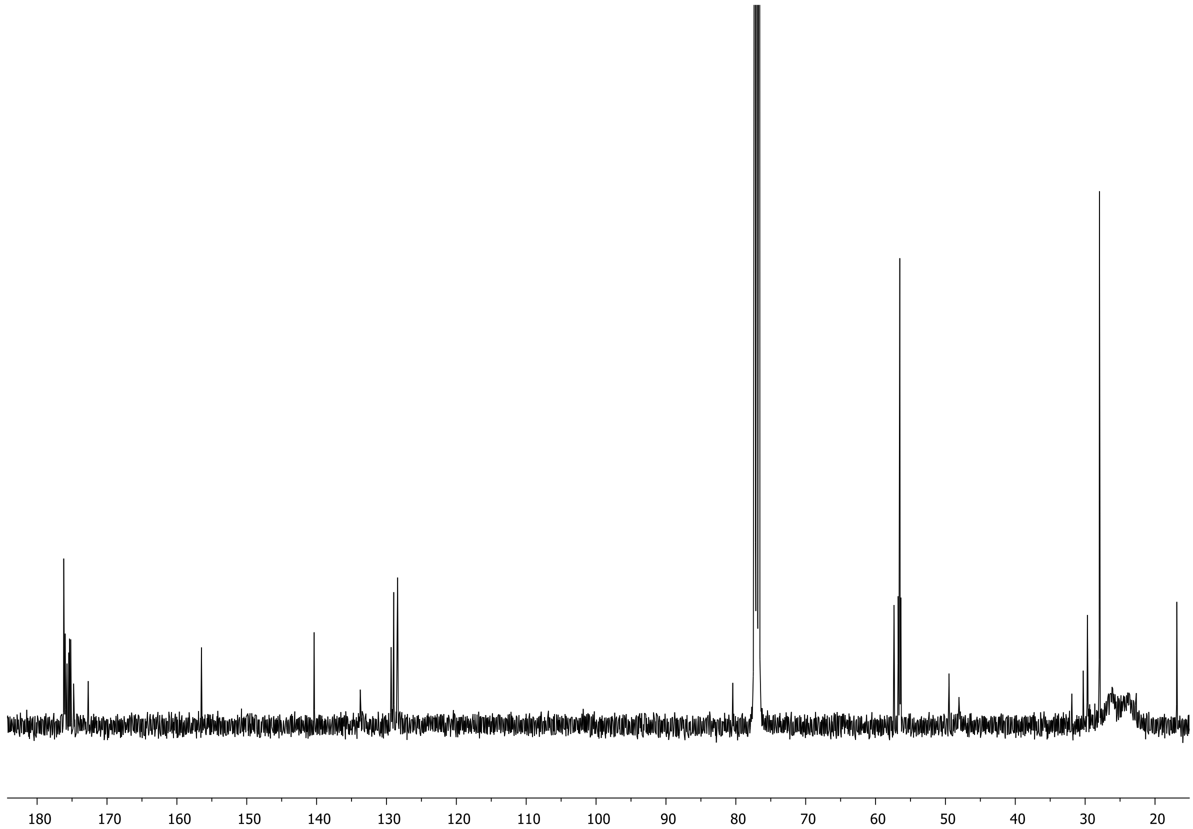

^{1}H-NMR of N_3-AibAib-OMe**

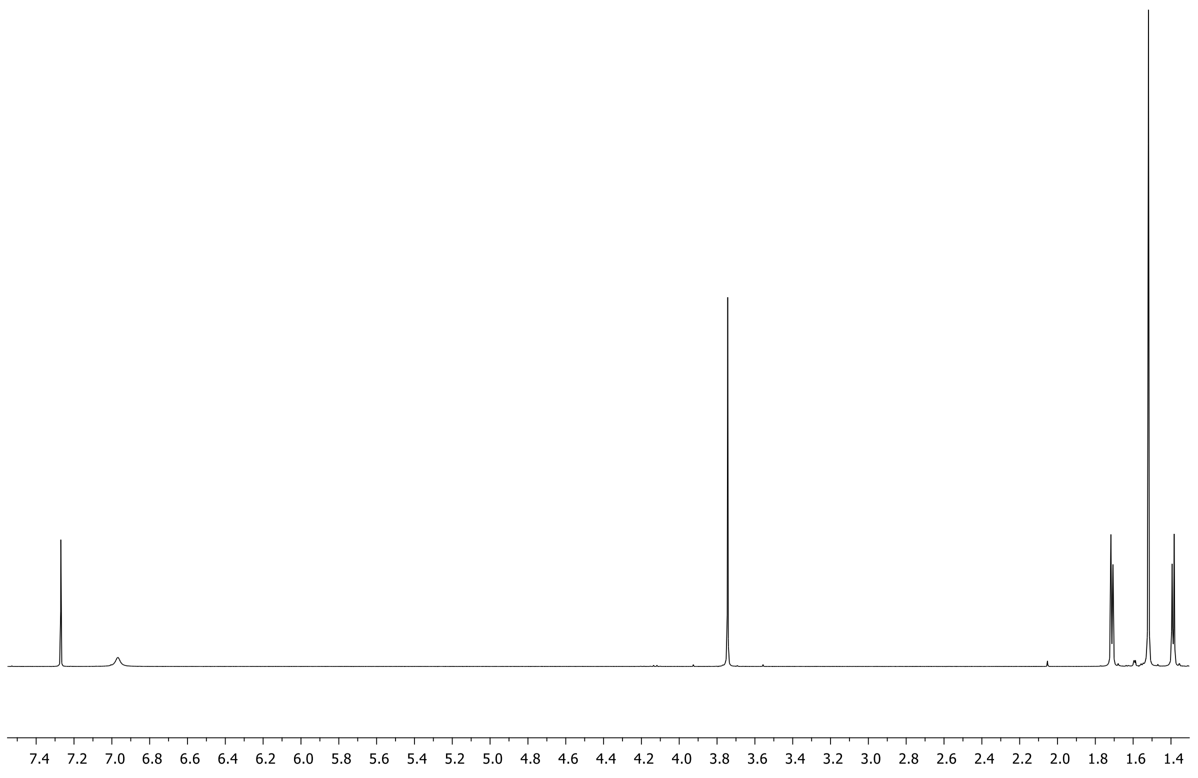

^{13}C-NMR of N_3-AibAib-OMe[7]**

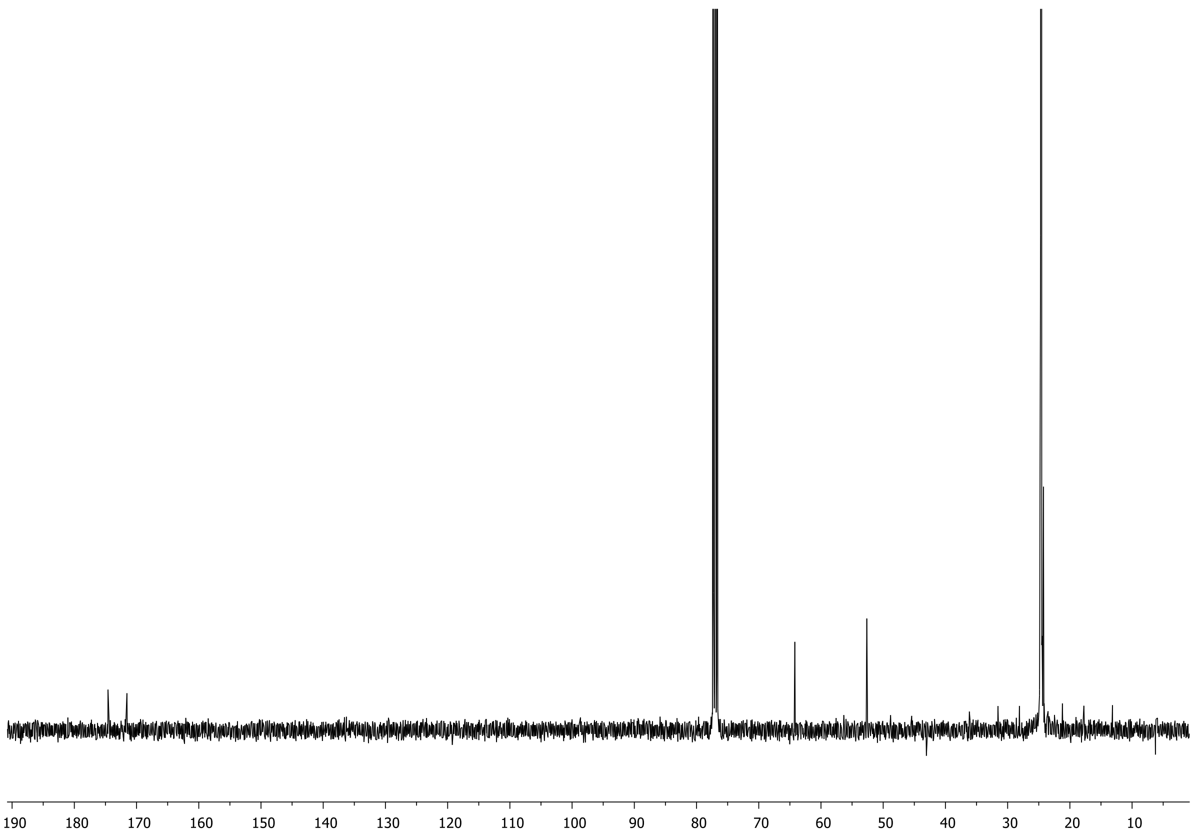

^{1}H-NMR of H-AibAib-OMe**

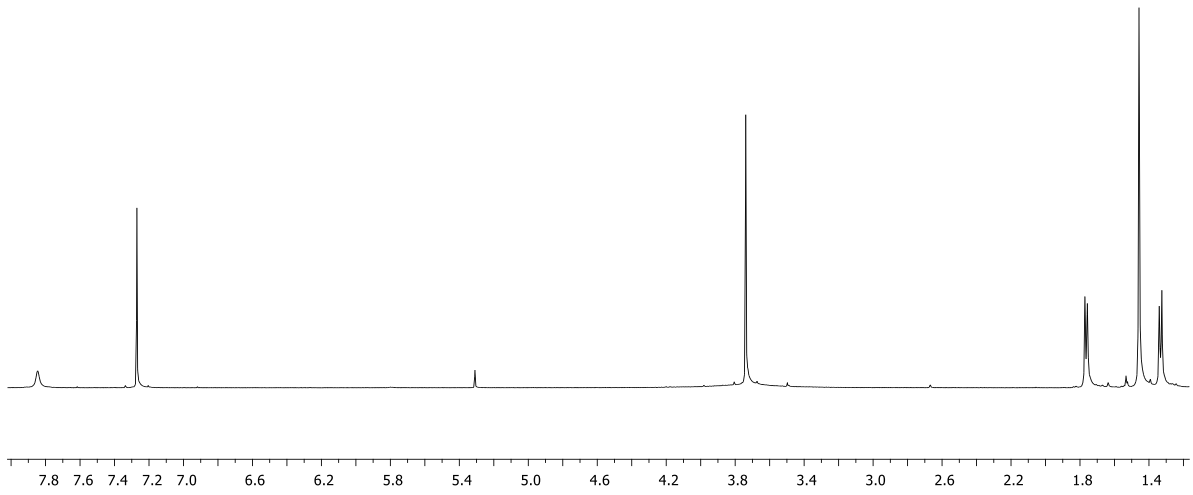

^{13}C-NMR of H-AibAib-OMe**

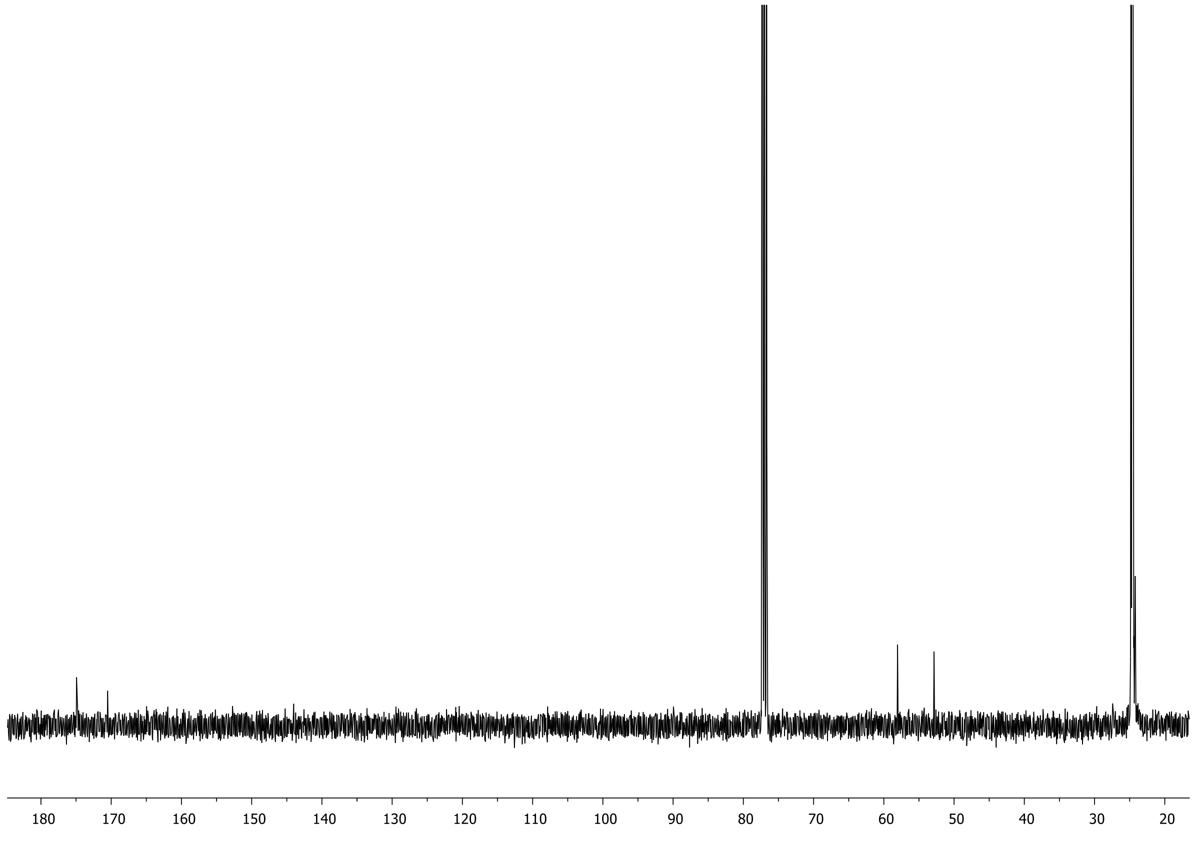

^{1}H-NMR of N_3-Aib$_2$Aib-OMe**

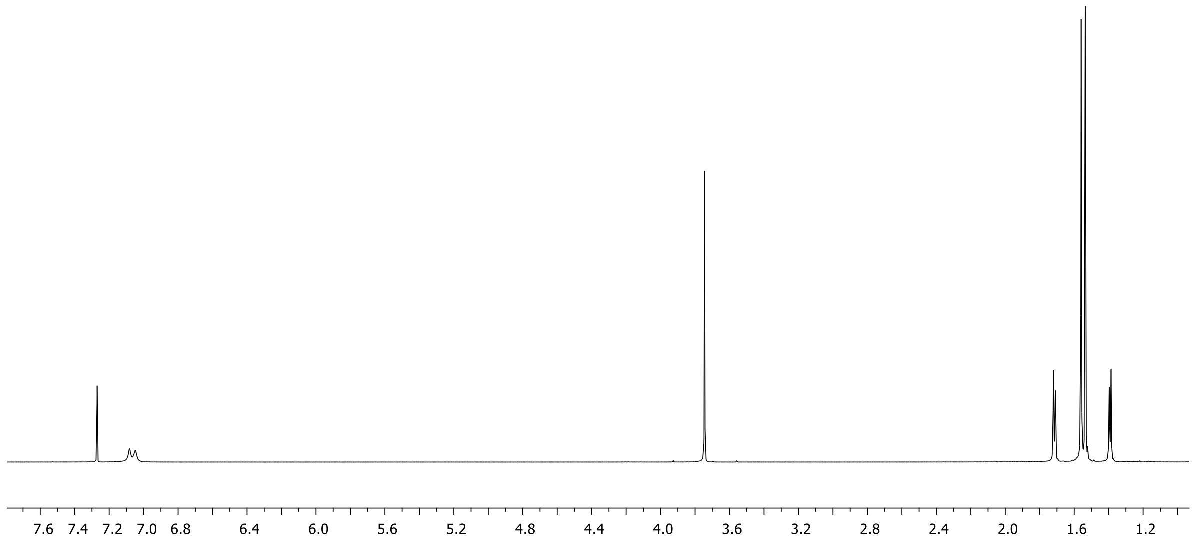

^{13}C-NMR of N_3-Aib$_2$Aib-OMe**

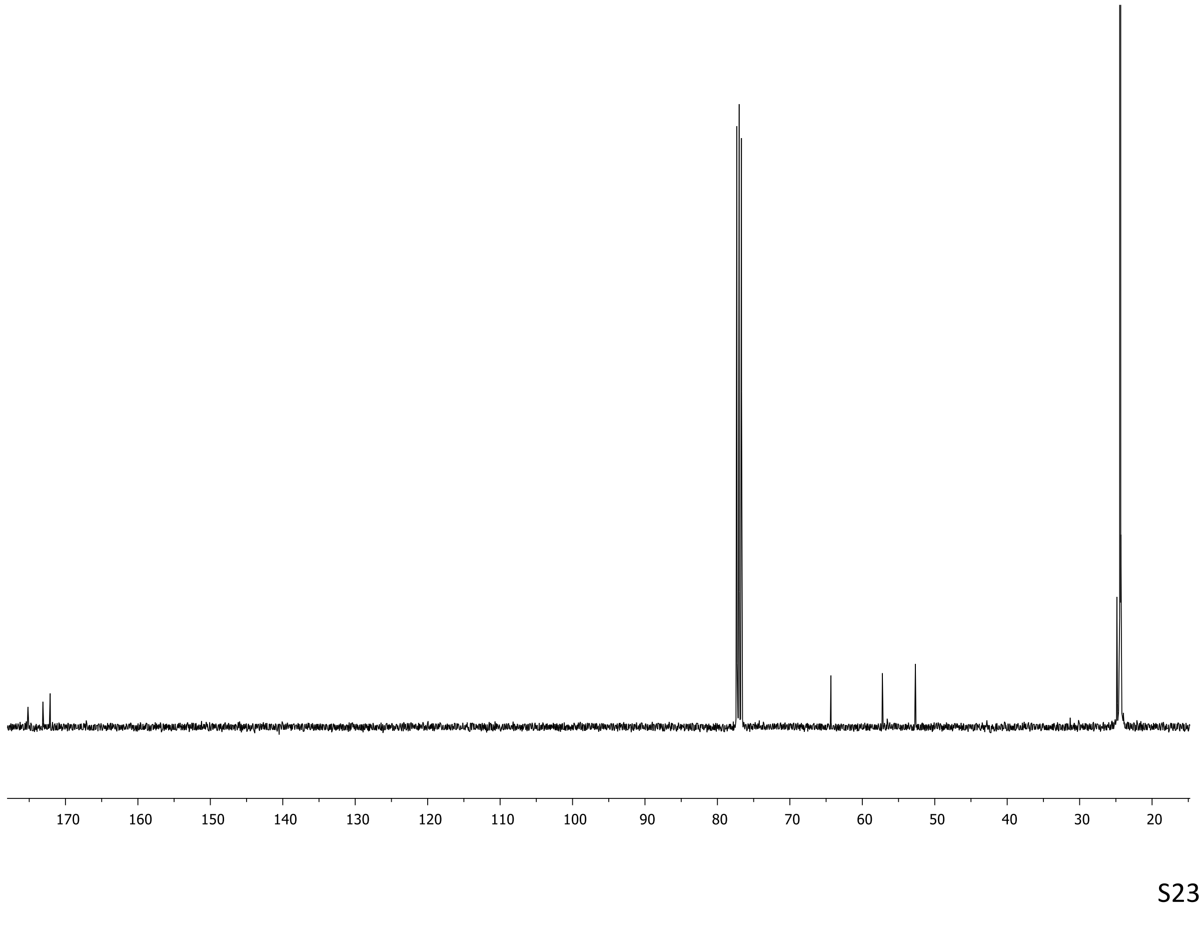

^{1}H-NMR of H-Aib$_2$Aib-OMe**

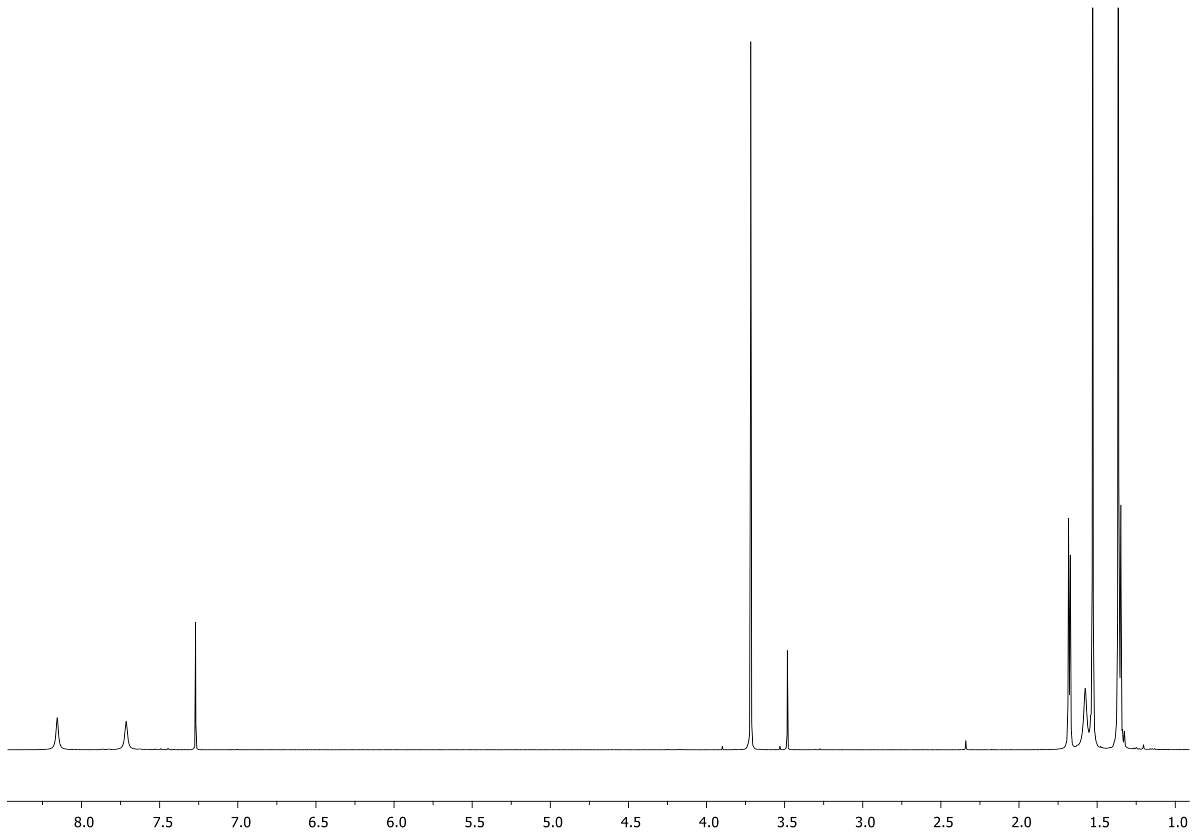

^{13}C-NMR of H-Aib$_2$Aib-OMe[7]**

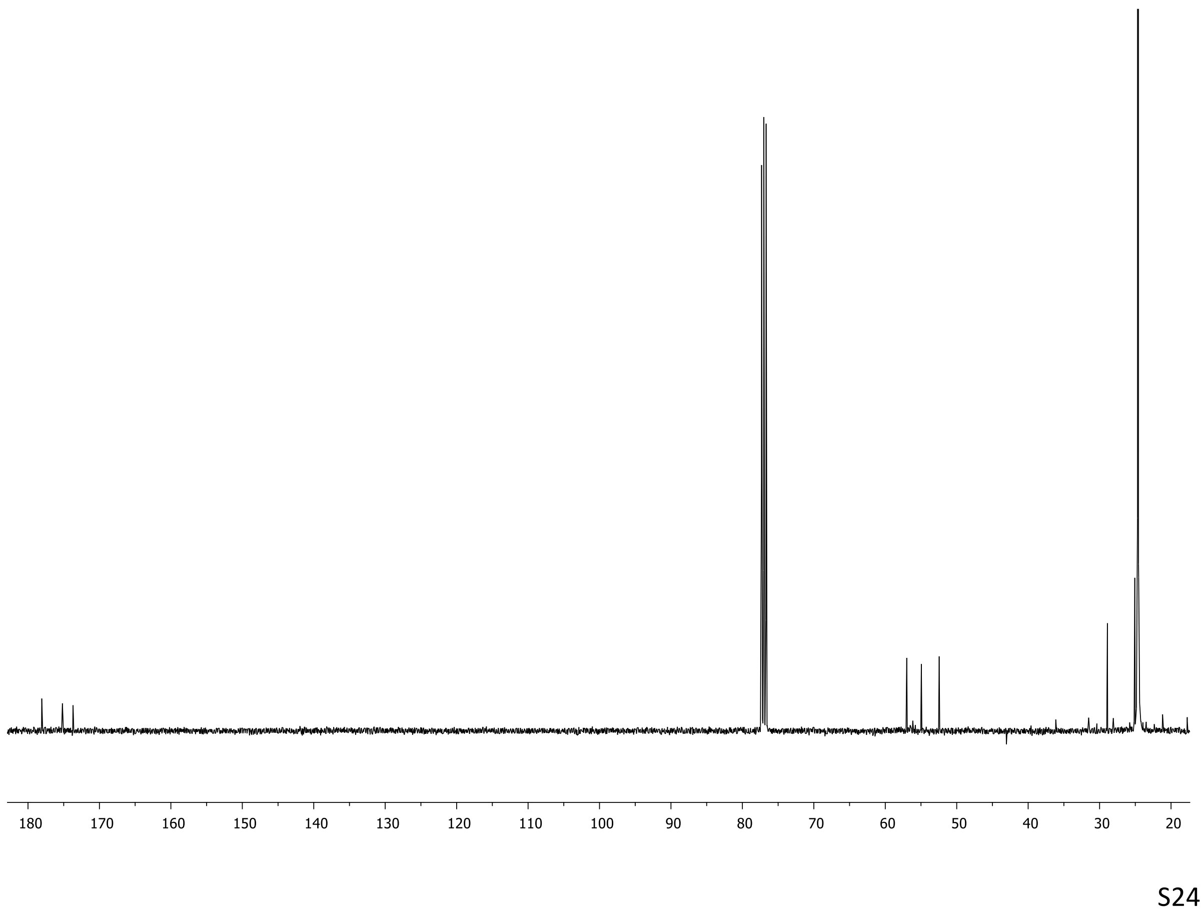

^{1}H-NMR of N_3-Aib$_2$Aib-OH 4****

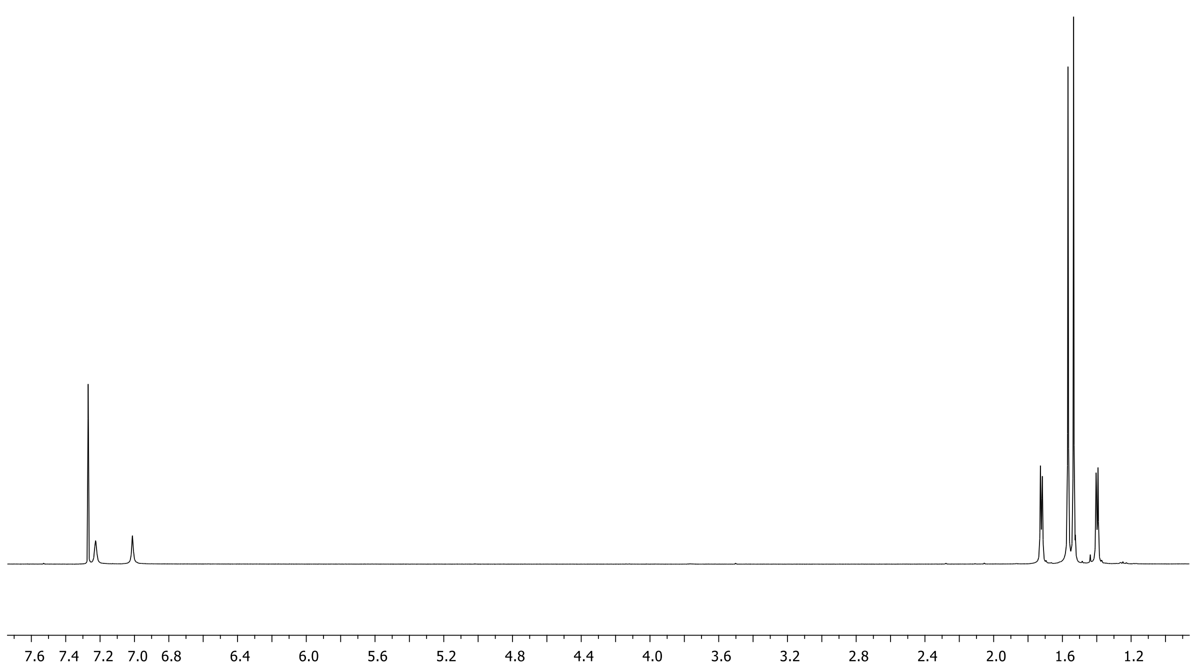

^{13}C-NMR of N_3-Aib$_2$Aib-OH 4****

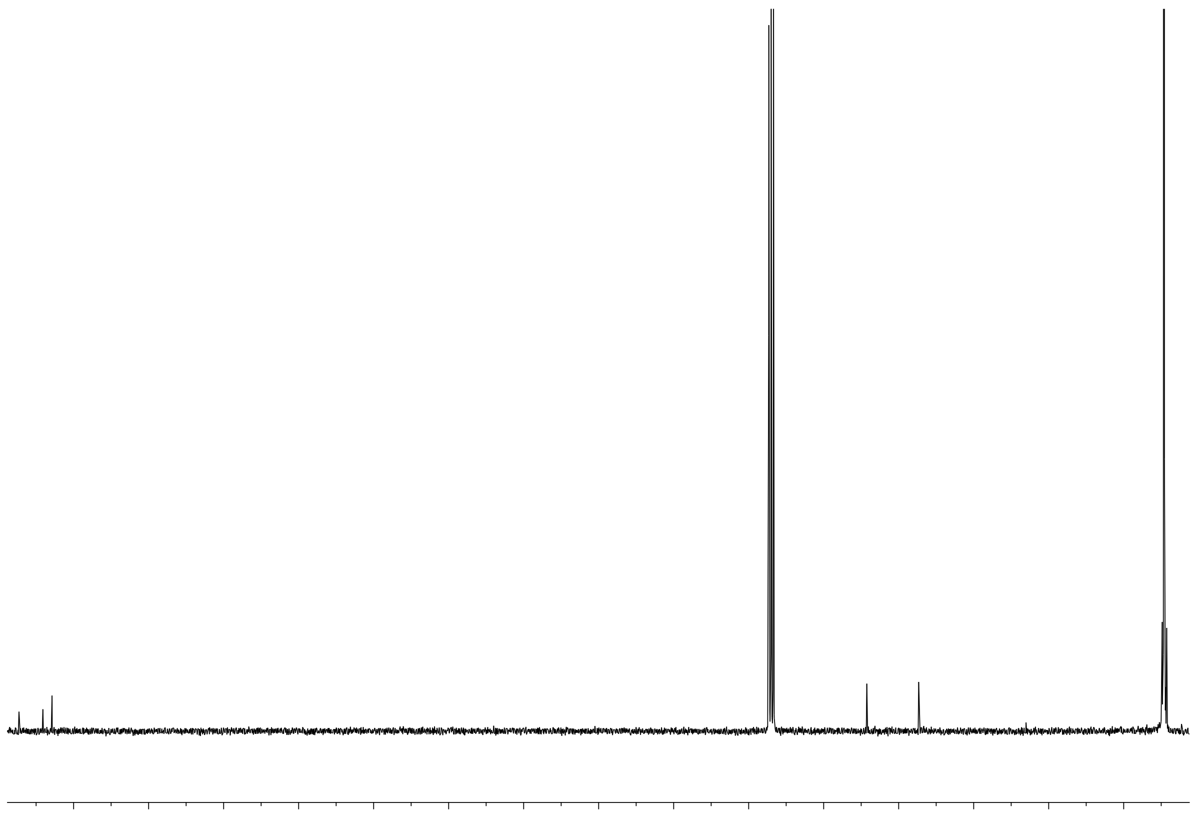

^{1}H-NMR of N_3-Aib$_2$Aib$^{50\%}$Aib$_2$Aib**-OMe**

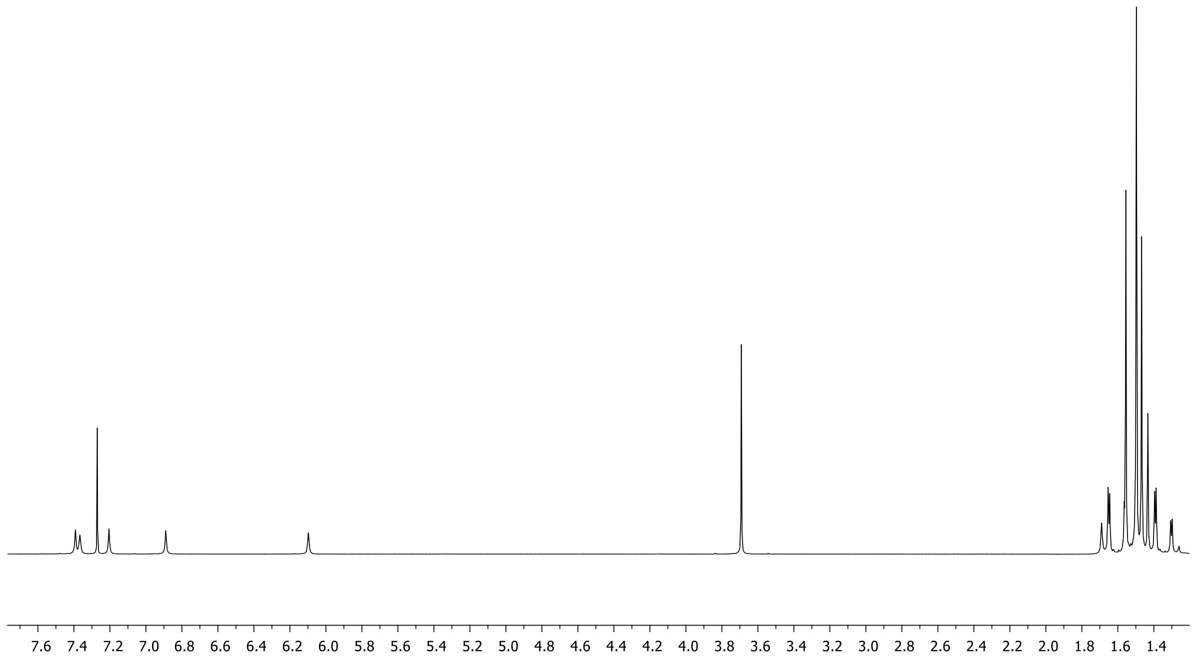

^{13}C-NMR of N_3-Aib$_2$Aib$^{50\%}$Aib$_2$Aib**-OMe**

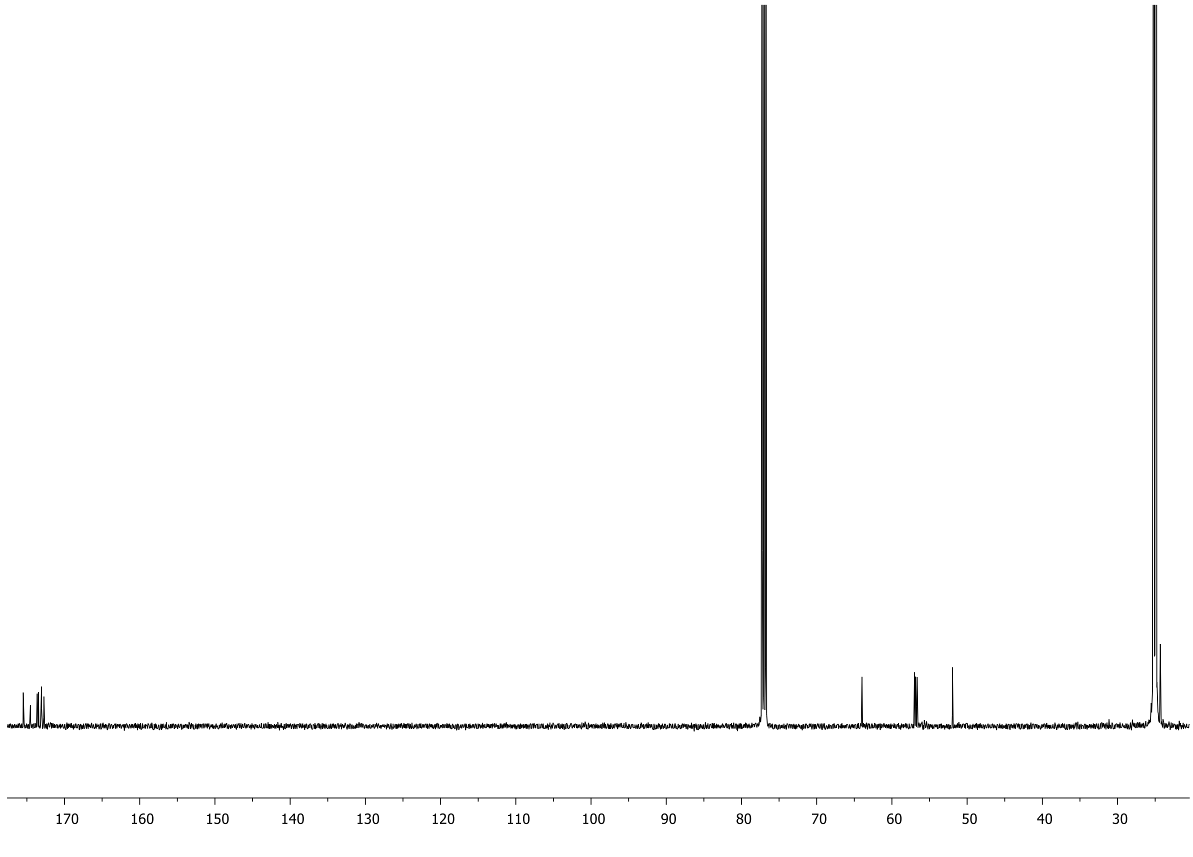

^{1}H-NMR of H-Aib$_2$Aib$^{50\%}$Aib$_2$Aib**-OMe**

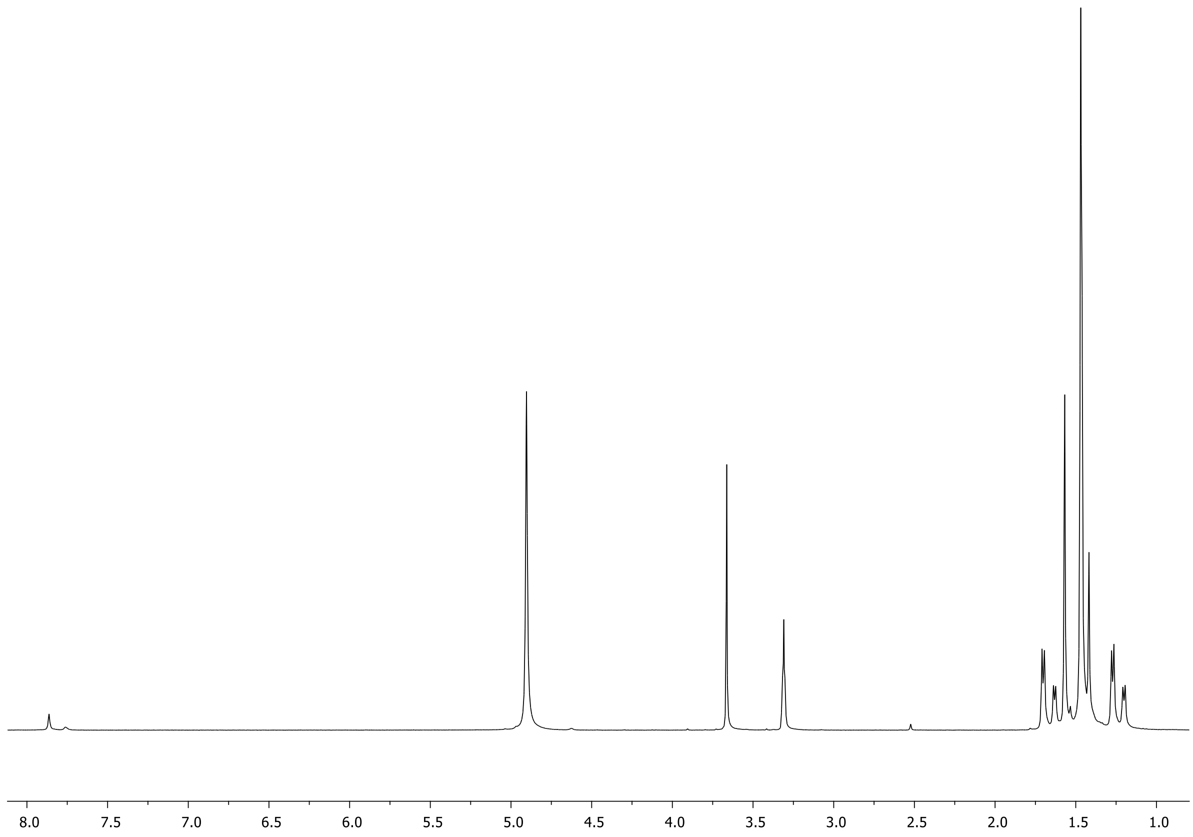

^{13}C-NMR of H-Aib$_2$Aib$^{50\%}$Aib$_2$Aib**-OMe**

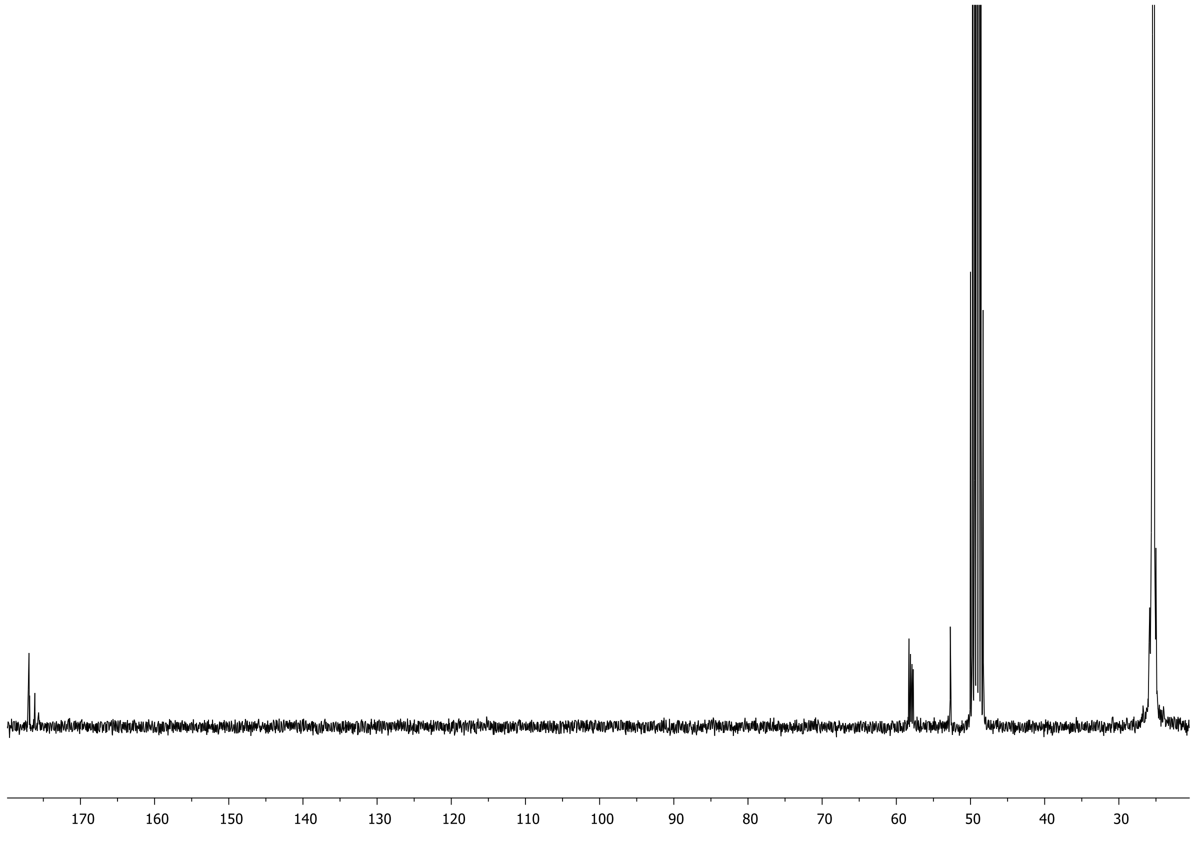

^{1}H-NMR of N_3-Aib$_2$Aib$^{25\%}$Aib$_2$Aib$^{50\%}$**Aib$_2$Aib**-OMe 5**

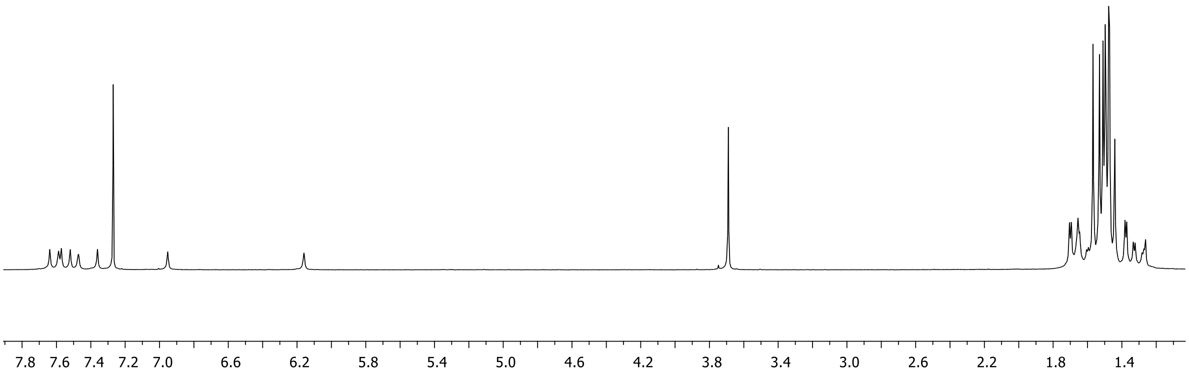

^{13}C-NMR of N_3-Aib$_2$Aib$^{25\%}$Aib$_2$Aib$^{50\%}$**Aib$_2$Aib**-OMe 5**

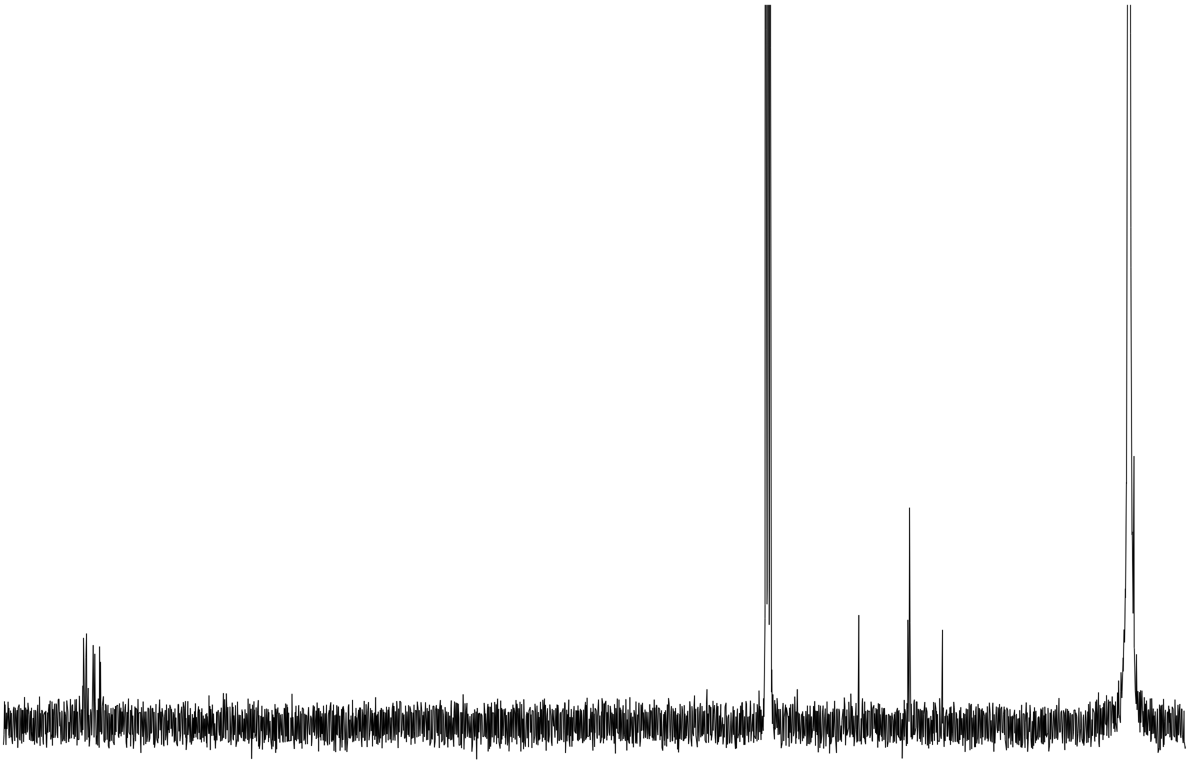

^{1}H-NMR of N_3-Aib$_2$Aib$^{25\%}$Aib$_2$Aib$^{50\%}$**Aib$_2$Aib**-OH**

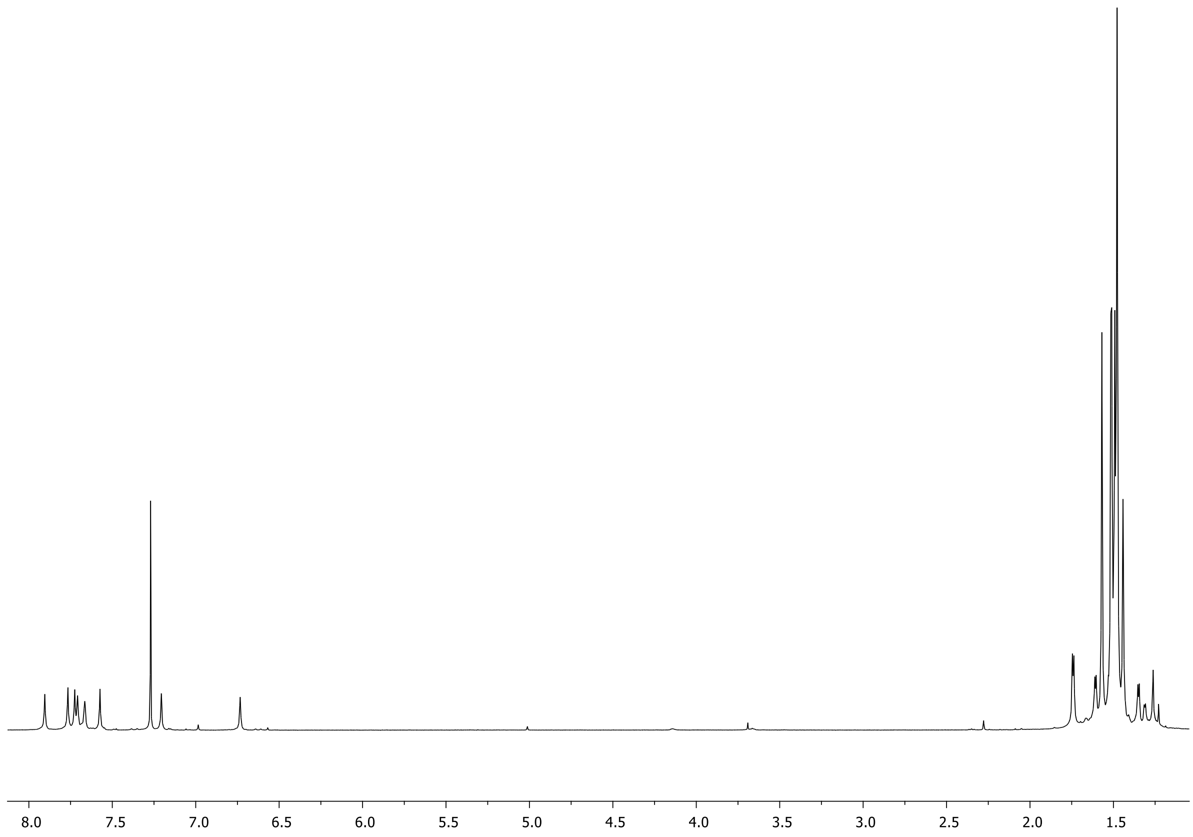

^{13}C-NMR of N_3-Aib$_2$Aib$^{25\%}$Aib$_2$Aib$^{50\%}$**Aib$_2$Aib**-OH**

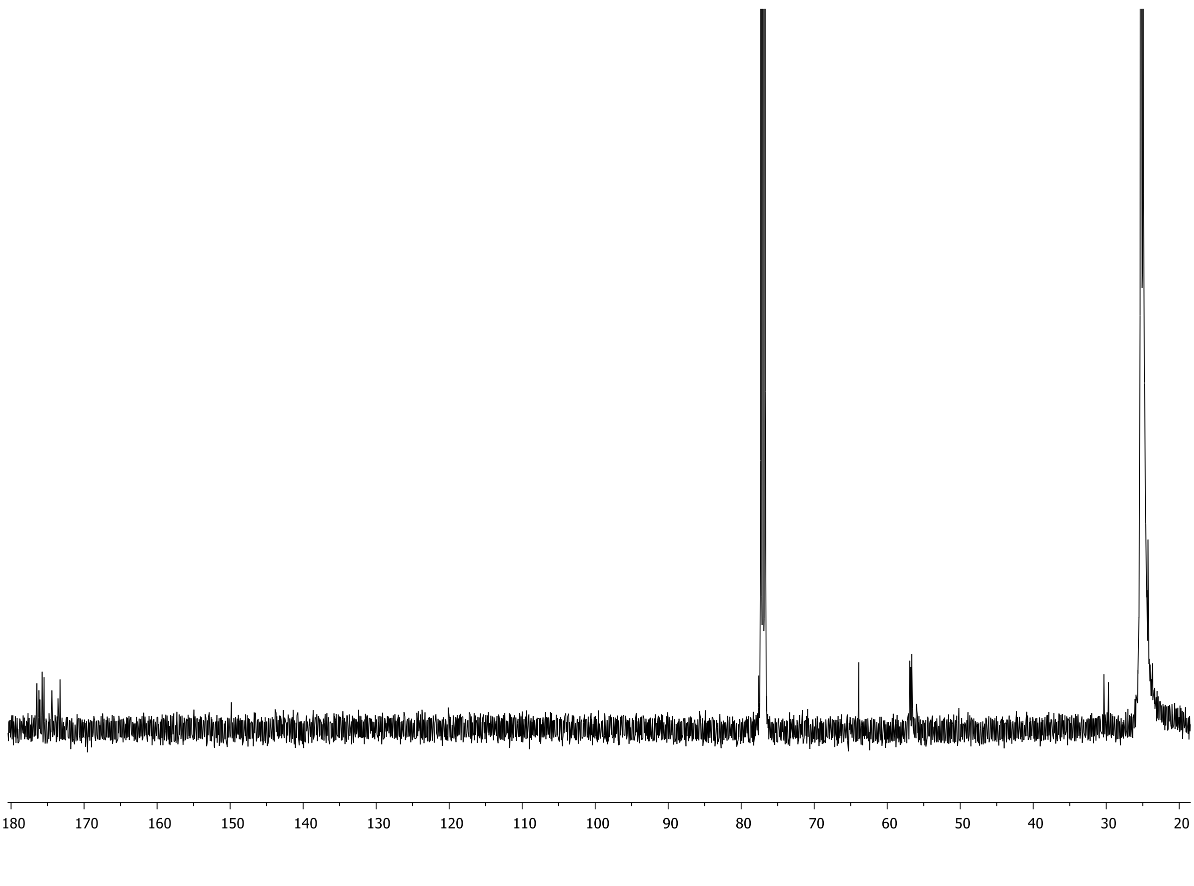

^{1}H-NMR of N_3-Aib$_2$Aib$^{25\%}$**Aib$_2$Aib$^{50\%}$**Aib$_2$Aib**Aib$_2$-O^{t}Bu 6a

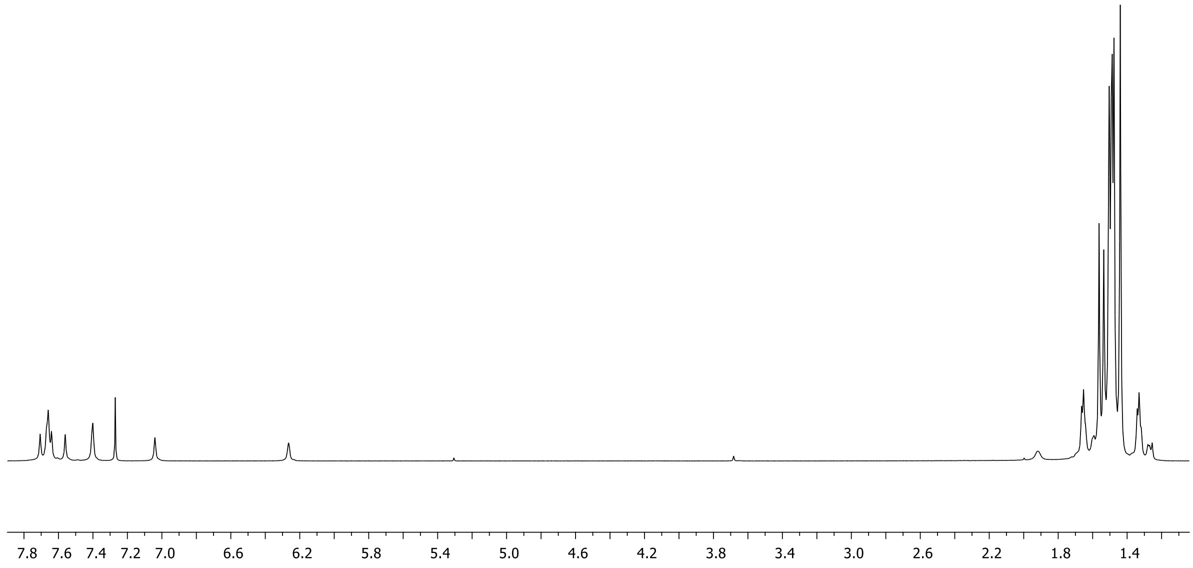

^{13}C-NMR of N_3-Aib$_2$Aib$^{25\%}$**Aib$_2$Aib$^{50\%}$**Aib$_2$Aib**Aib$_2$-O^{t}Bu 6a

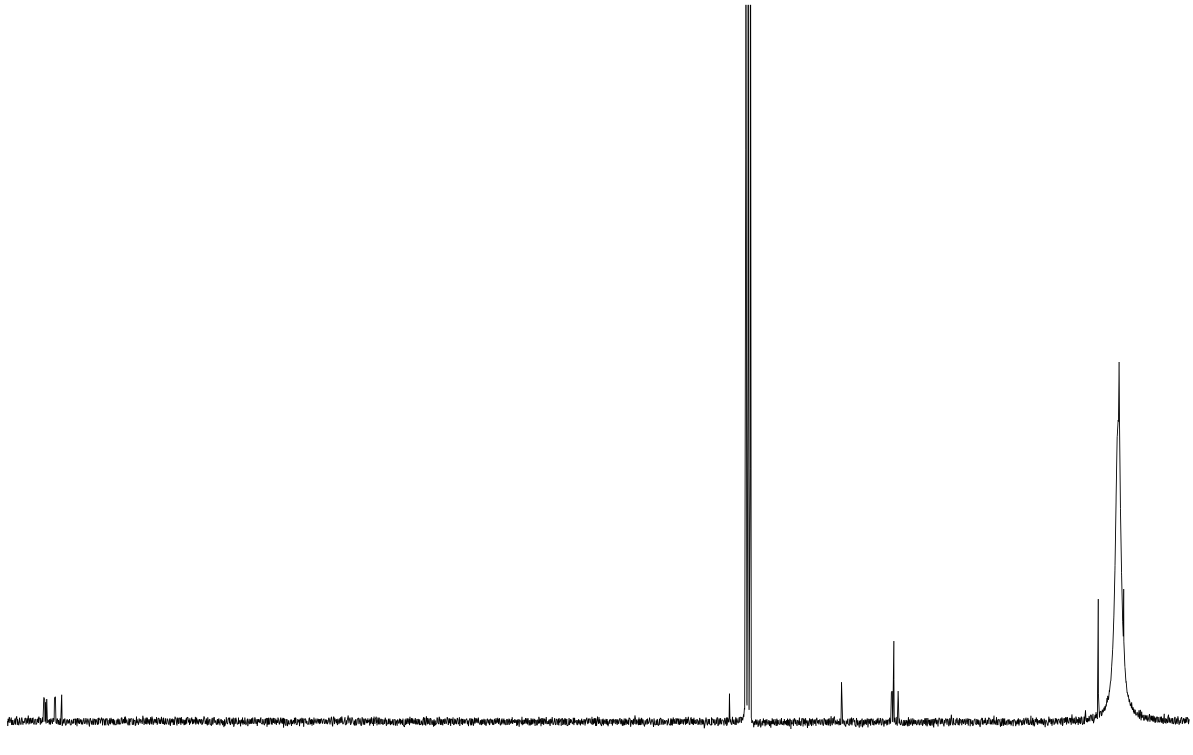

^{1}H-NMR of N_3-Aib$_2$Aib$^{25\%}$Aib$_2$Aib$^{50\%}$**Aib$_2$Aib**Aib$_3$O^{t}Bu 6b**

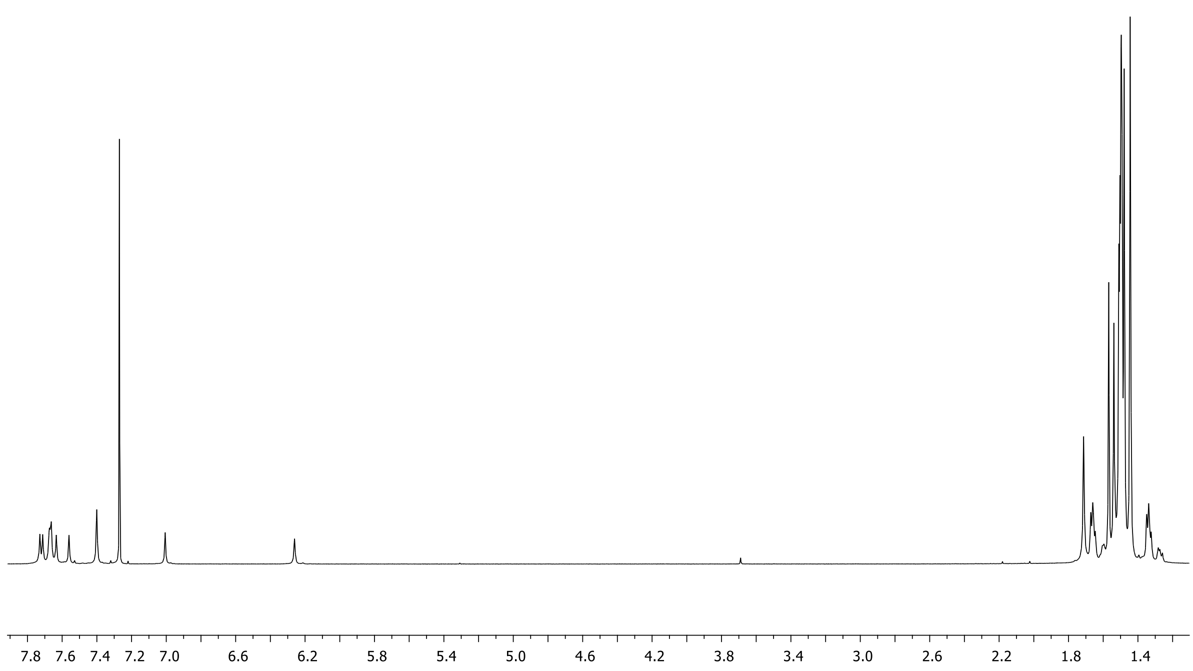

^{13}C-NMR of N_3-Aib$_2$Aib$^{25\%}$Aib$_2$Aib$^{50\%}$**Aib$_2$Aib** 6b**

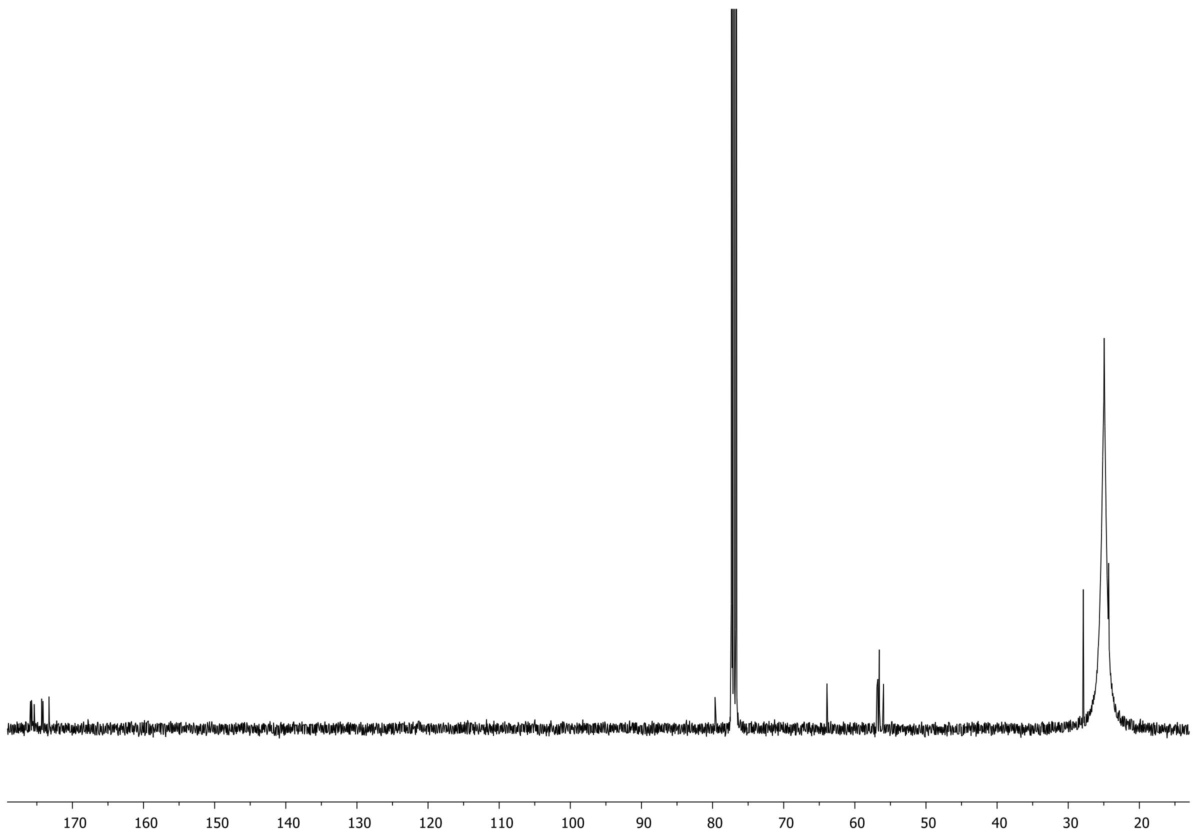

^{1}H-NMR of N_3-Aib$_2$Aib$^{25\%}$Aib$_2$Aib$^{50\%}$**Aib$_2$Aib**Aib$_4$O^{t}Bu 6c**

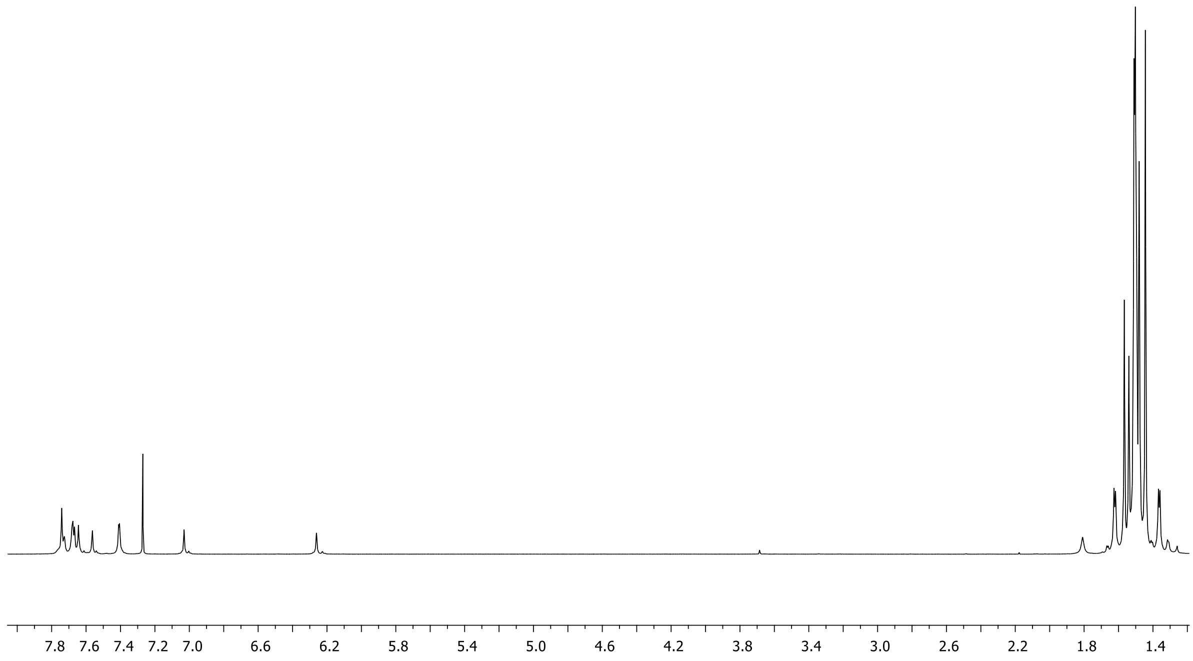

^{13}C-NMR of N_3-Aib$_2$Aib$^{25\%}$Aib$_2$Aib$^{50\%}$**Aib$_2$Aib**Aib$_4$O^{t}Bu 6c**

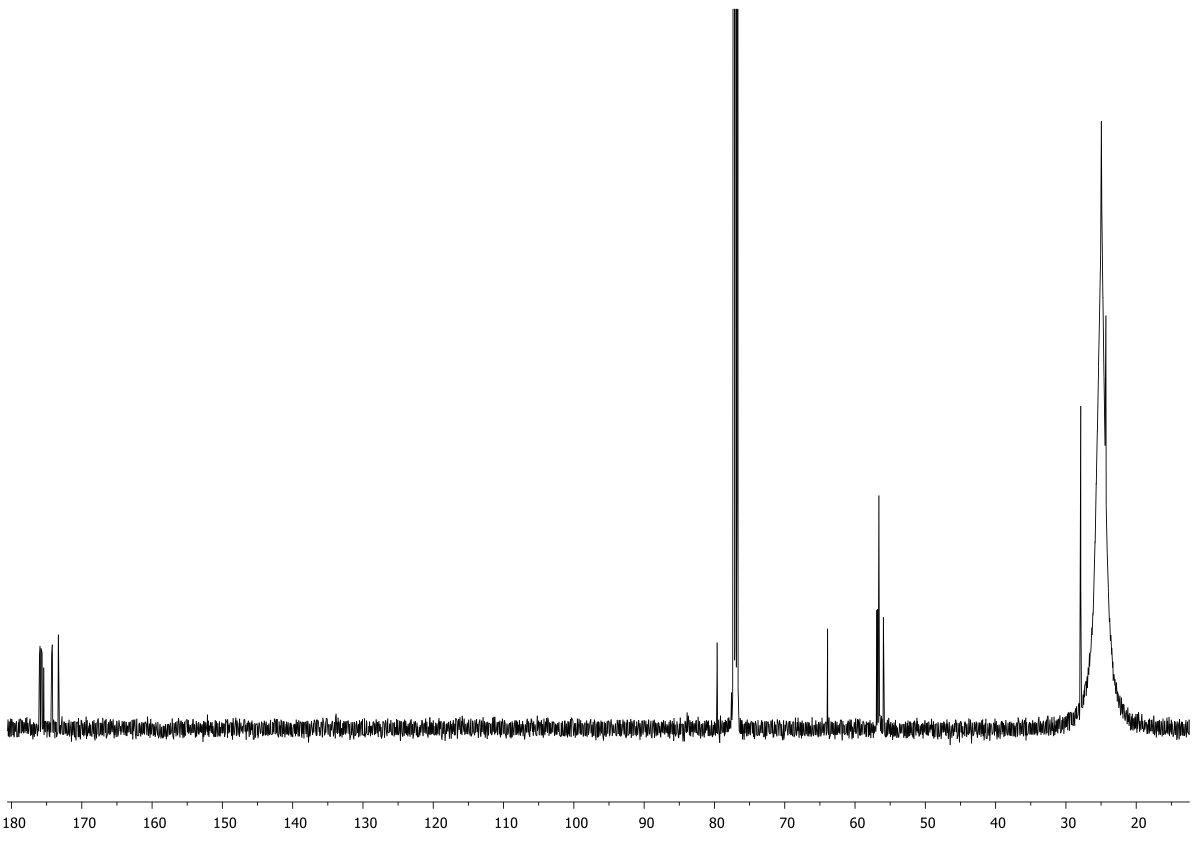

^{1}H-NMR of N_3-Aib$_2$Aib$^{25\%}$**Aib$_2$Aib$^{50\%}$**Aib$_2$Aib**Aib$_2$-OH

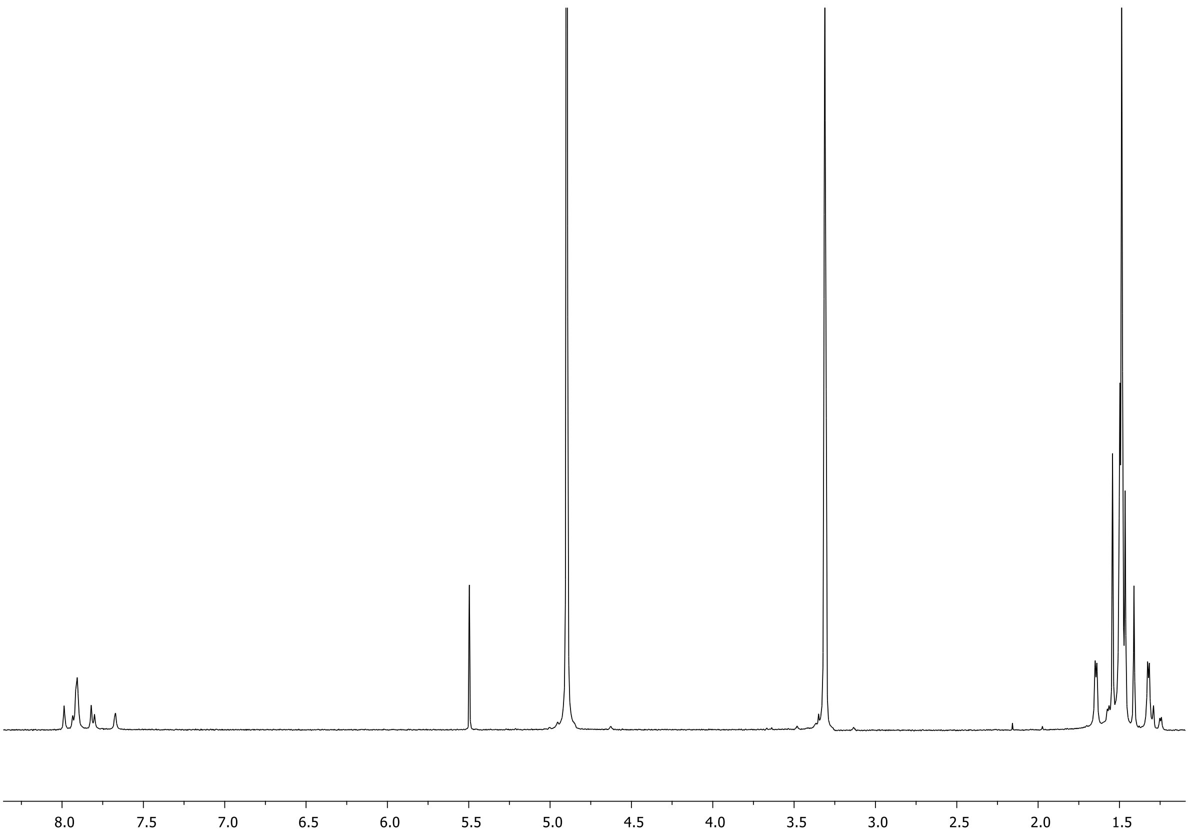

^{13}C-NMR of N_3-Aib$_2$Aib$^{25\%}$**Aib$_2$Aib$^{50\%}$**Aib$_2$Aib**Aib$_2$-OH

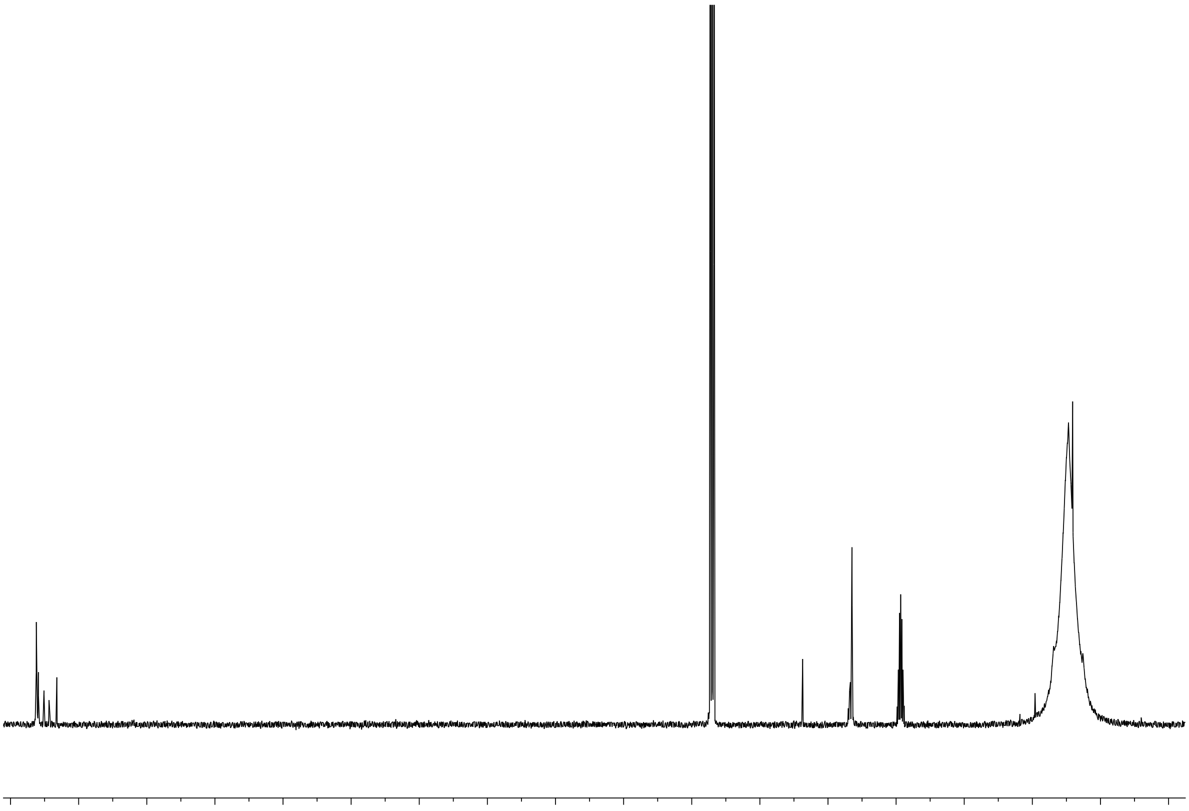

^{1}H-NMR of N_3-$Aib_2Aib^{25\%}$ $Aib_2Aib^{50\%}$**Aib_2Aib**Aib_3-OH**

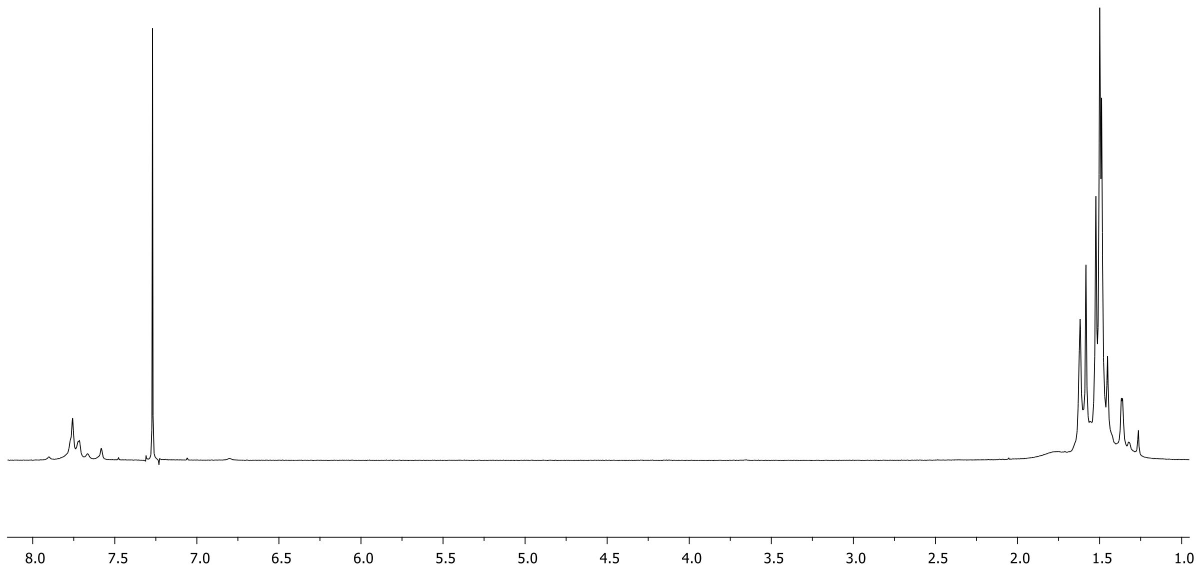

^{13}C-NMR of N_3-$Aib_2Aib^{25\%}$ $Aib_2Aib^{50\%}$**Aib_2Aib**Aib_3-OH**

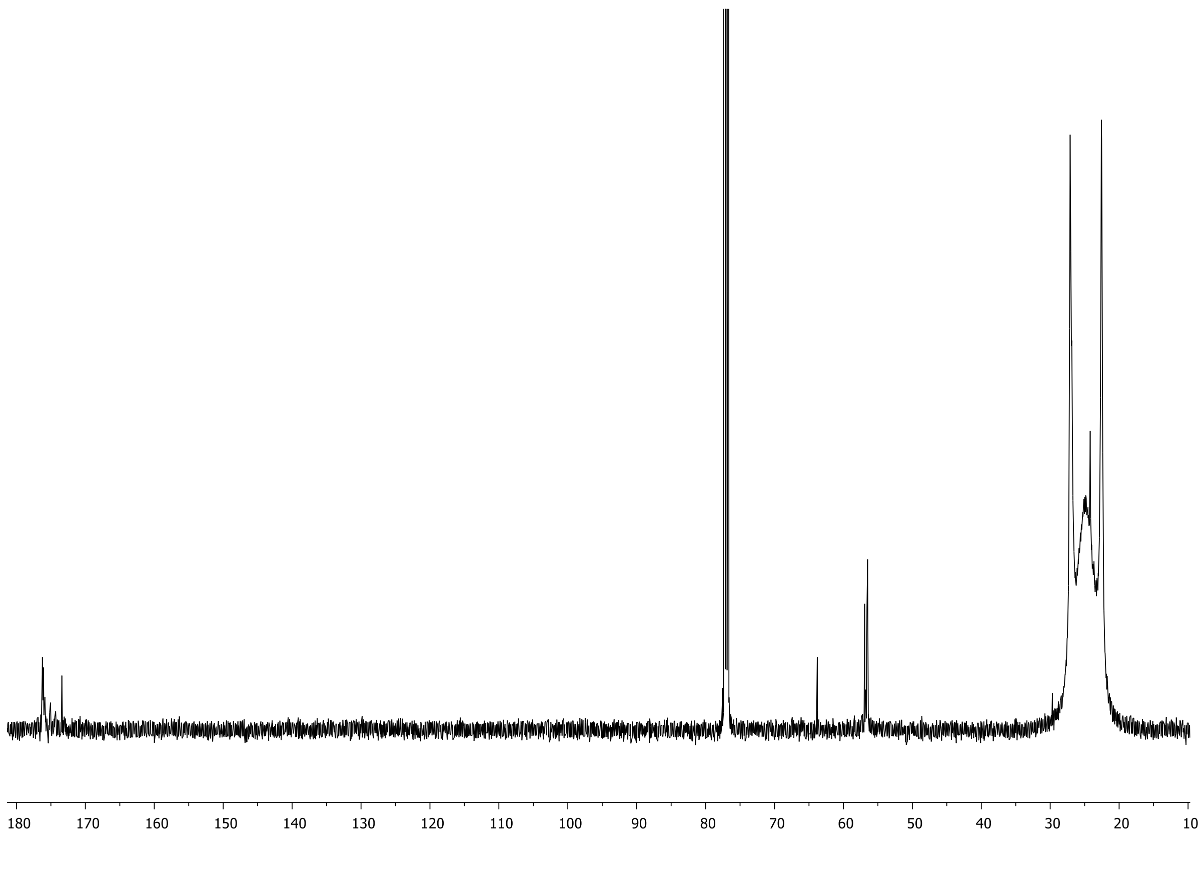

^{1}H-NMR of N_3-$Aib_2Aib^{25\%}$ $Aib_2Aib^{50\%}$**Aib_2Aib**Aib_4-OH**

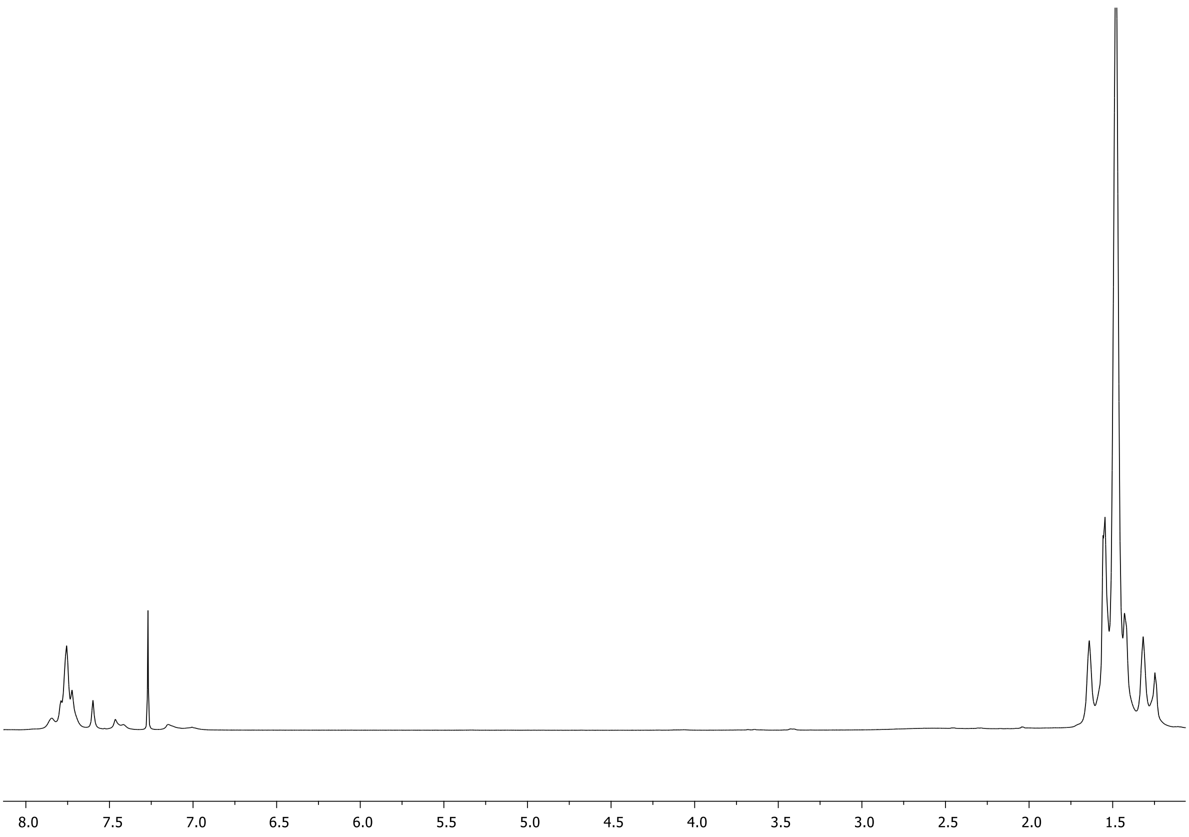

^{13}C-NMR of N_3-$Aib_2Aib^{25\%}$ $Aib_2Aib^{50\%}$**Aib_2Aib**Aib_4-OH**

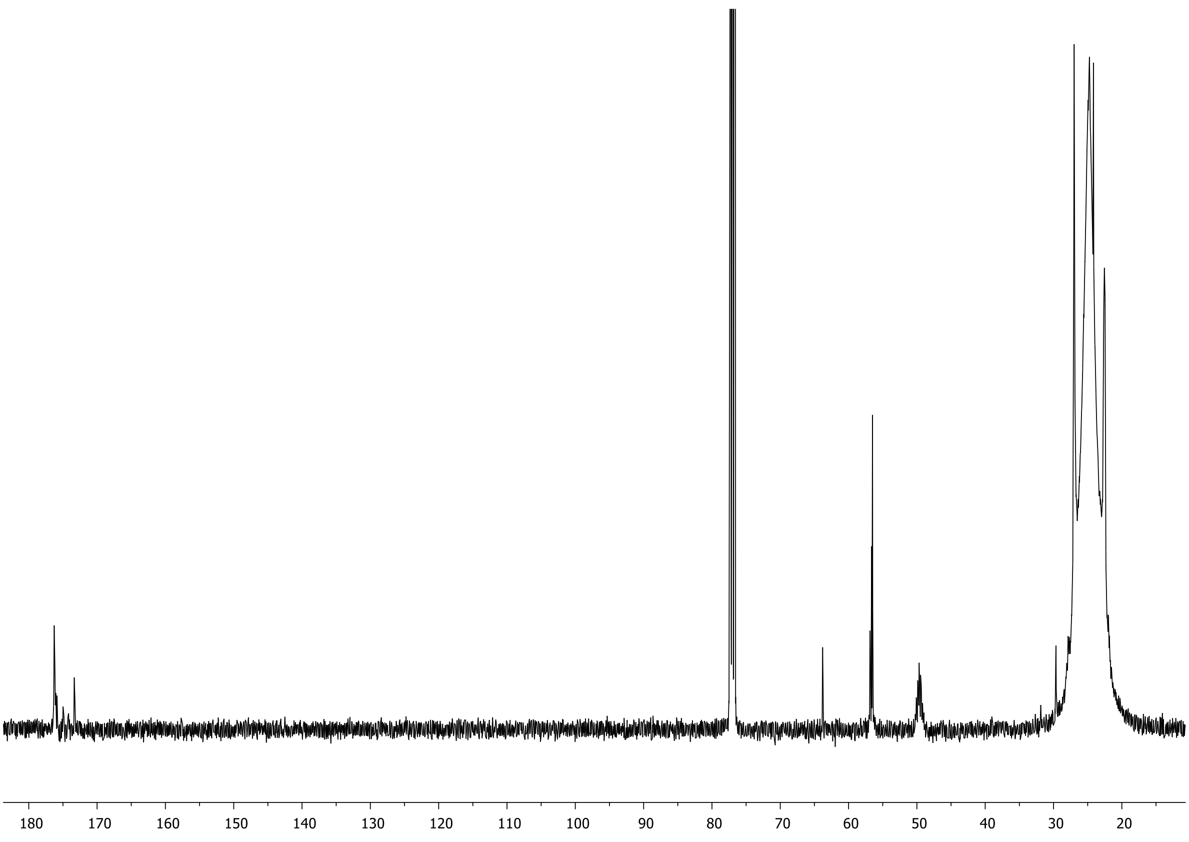

^{1}H-NMR of N_3-$Aib_2Aib^{25\%}$ $Aib_2Aib^{50\%}$**Aib_2Aib**Aib_2Ala-$NH^{t}Bu$ 7a**

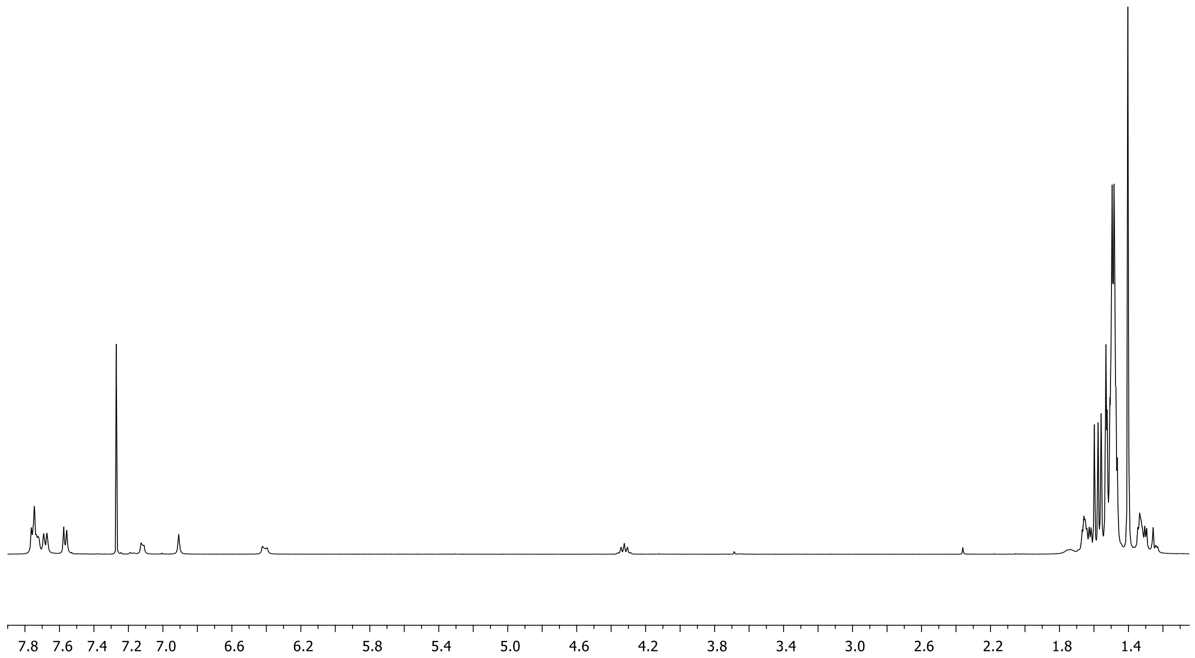

^{13}C-NMR of N_3-$Aib_2Aib^{25\%}$ $Aib_2Aib^{50\%}$**Aib_2Aib**Aib_2Ala-$NH^{t}Bu$ 7a**

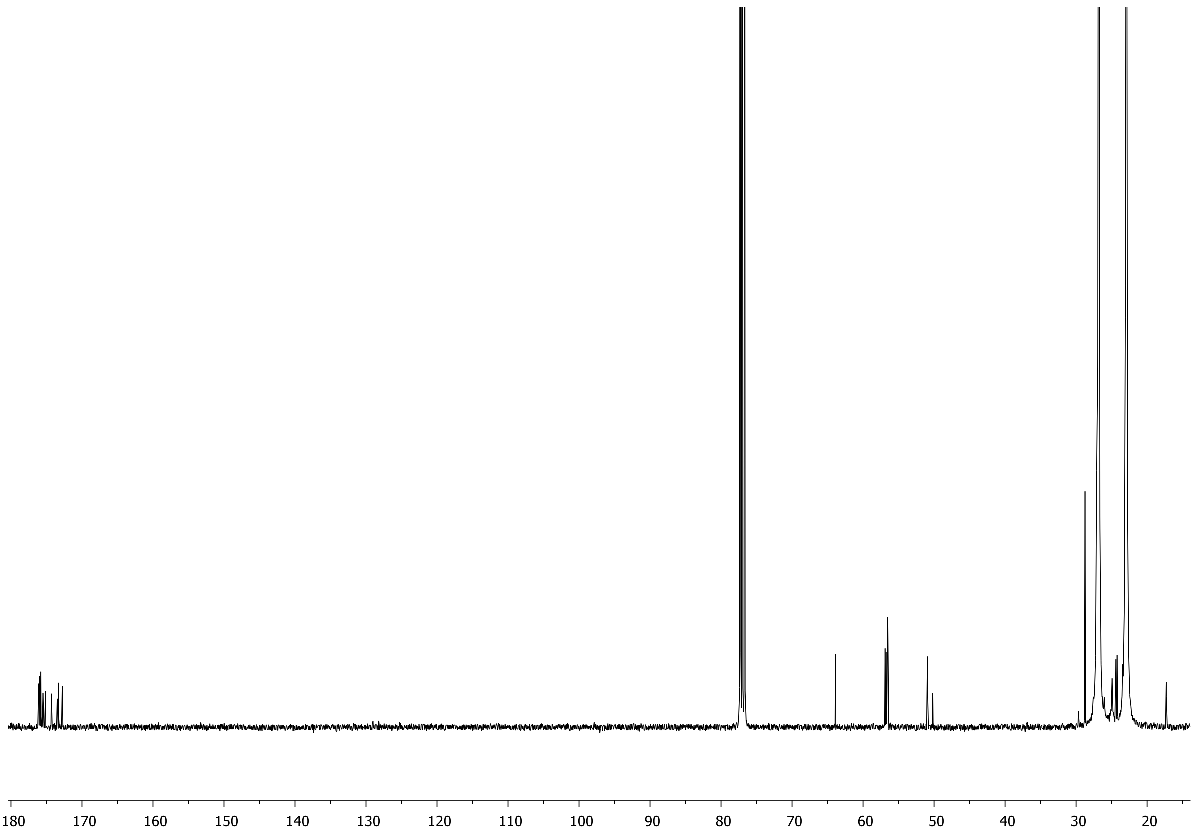

^{1}H-NMR of N_3-Aib$_2$Aib$^{25\%}$Aib$_2$Aib$^{50\%}$**Aib$_2$Aib**Aib$_3$Ala-NH^{t}Bu 7b**

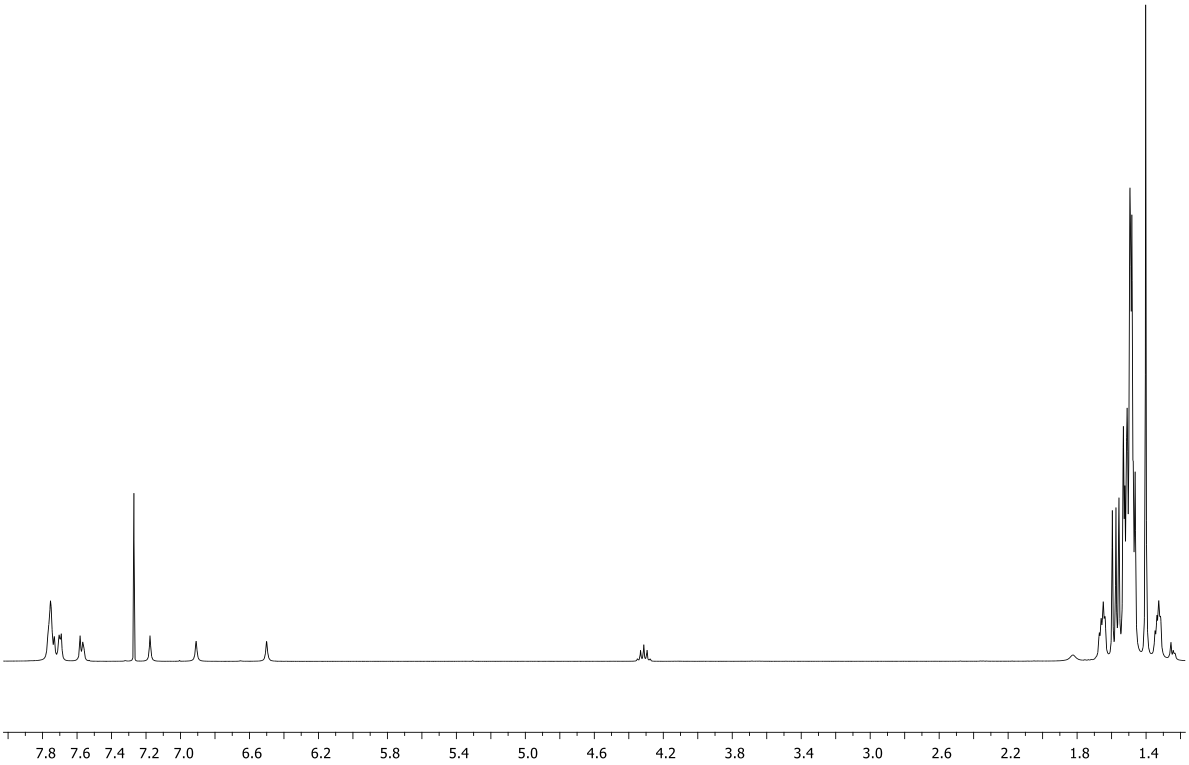

^{13}C-NMR of N_3-Aib$_2$Aib$^{25\%}$Aib$_2$Aib$^{50\%}$**Aib$_2$Aib**Aib$_3$Ala-NH^{t}Bu 7b**

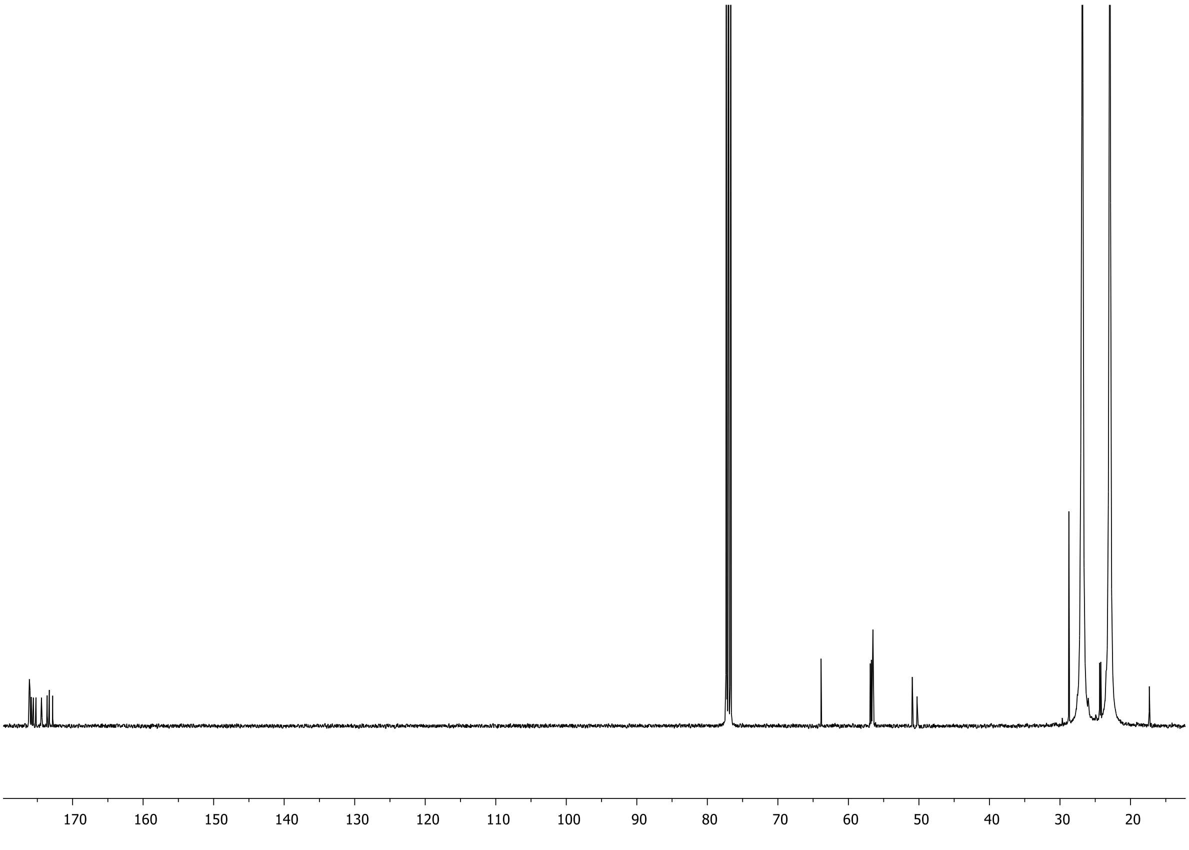

^{1}H-NMR of N_3-Aib$_2$Aib$^{25\%}$Aib$_2$Aib$^{50\%}$**Aib$_2$Aib**Aib$_4$Ala-NH^{t}Bu 7c**

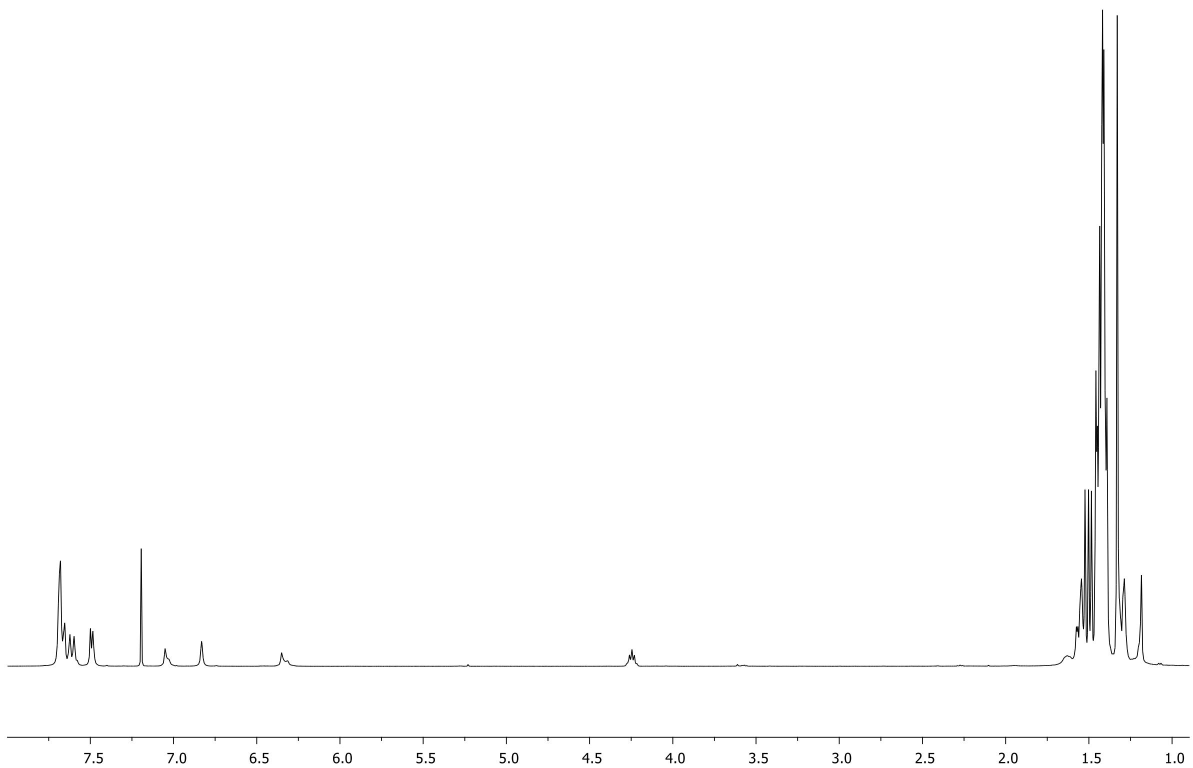

^{13}C-NMR of N_3-Aib$_2$Aib$^{25\%}$Aib$_2$Aib$^{50\%}$**Aib$_2$Aib**Aib$_4$Ala-NH^{t}Bu 7c**

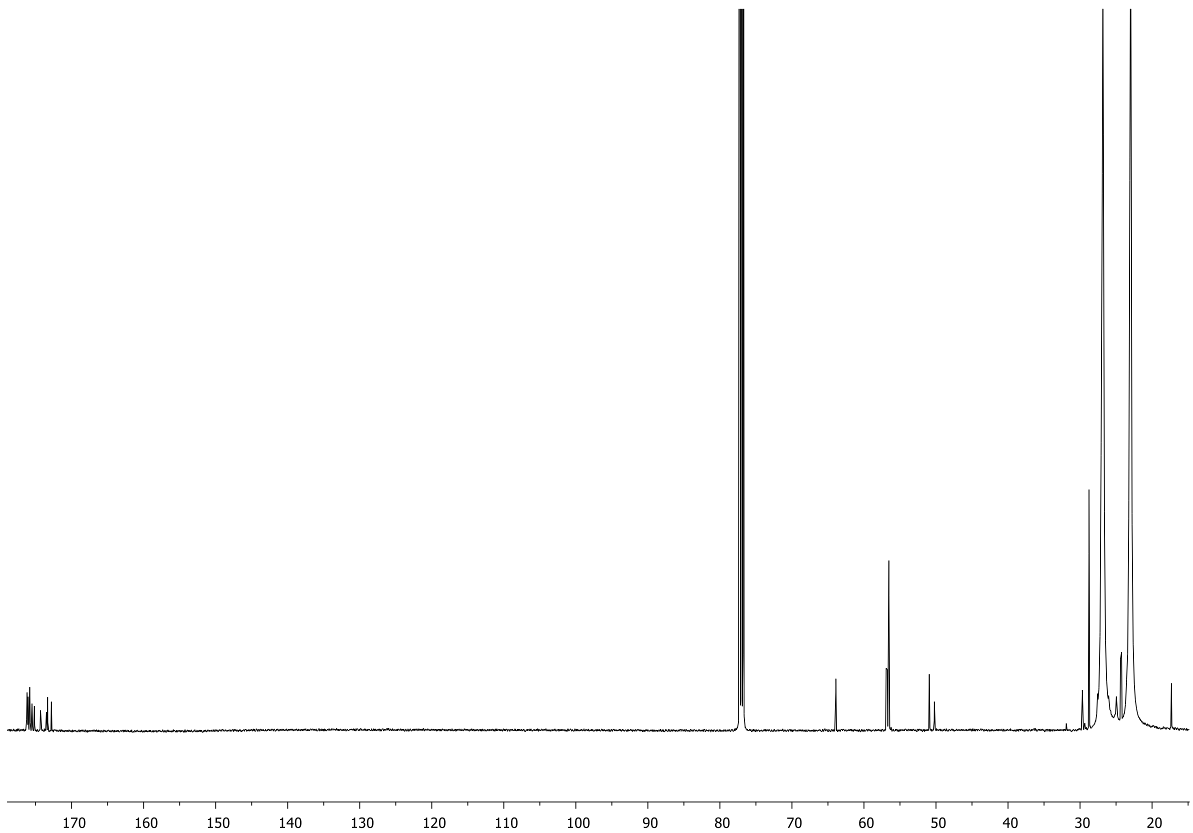

Notes and References

1. S. P. Fletcher, J. Solà, D. Holt, R. A. Brown and J. Clayden, *Beilstein J. Org. Chem.* **2011**, 7, 1304
2. J. Clayden, A. Castellanos, J. Solà and G. A. Morris, *Angew. Chemie Int. Ed.* **2009**, *48,* 5962
3. S. J. Pike, S. Webb and J. Clayden, *Org. Biomol. Chem* **2014**, *12*, 4124
4. L. Byrne, J. Solà, T. Boddaert, T. Marcelli, R. W. Adams, G. A. Morris and J. Clayden, *Angew. Chemie Int. Ed.* **2014**, *53,* 151
5. M. Foroozandeh, R. W. Adams, N. J. Meharry, D. Jeannerat, M. Nilsson and G. A. Morris, *Angew. Chemie Int. Ed.* **2014**, *53,* 6990
6. M. Foroozandeh, R. W. Adams, D. Jeannerat, M. Nilsson and G. A. Morris, *J. Am. Chem. Soc.* **2014**, *136*, 11867
7. The symmetrical signals observed around the ^{13}C-labelled *CH_3 group are from composite pulse decoupling artefacts.
